# Condensation of Diacetyl with Alkyl Amines: Synthesis and Reactivity of *p*-Iminobenzoquinones and *p*-Diiminobenzoquinones

**DOI:** 10.3390/molecules201119716

**Published:** 2015-11-20

**Authors:** Carlos Espinoza-Hicks, Rafael Bautista, Saúl Frias-Puente, Vanessa Pelayo, Eder I. Martínez-Mora, Francisco Delgado, Joaquín Tamariz

**Affiliations:** Departamento de Química Orgánica, Escuela Nacional de Ciencias Biológicas, Instituto Politécnico Nacional. Prol. Carpio y Plan de Ayala, 11340 México, Mexico; hick_1107@hotmail.com (C.E.-H.); jimbe31@hotmail.com (R.B.); eleanor_500m@hotmail.com (S.F.-P.); fiosara29@gmail.com (V.P.); eder.qfb@gmail.com (E.I.M.-M.); jfdelgador@gmail.com (F.D.)

**Keywords:** diacetyl, *p*-iminobenzoquinones, *p*-diiminobenzoquinones, *N*,*N*′-dialkyl-1,4-diaminobenzenes, diarylamines

## Abstract

Condensation reactions between diacetyl and α-branched primary alkylamines under mild and neutral conditions provided a mixture of 2,5-dimethylbenzoquinone(alkylimines), 2,5-dimethylbenzoquinone(*bis*-alkyldiimines), and *N*,*N*′-dialkyl-2,5-dimethylbenzene-1,4-diamines, which were efficiently separated as pure products by column chromatography. Both 2,5-dimethylbenzoquinone(alkylimines) and 2,5-dimethylbenzoquinone(*bis*-alkyldiimines) underwent an interchange of the alkylimino group when treated with anilines, followed by reductive aromatization, to provide diarylamines and 1,4-dianilinobenzenes, respectively. Evaluation was also made of the reactivity and selectivity of these compounds in the presence of anilines, thiophenols and alkylhalides.

## 1. Introduction

Diacetyl (**1a**), a yellow liquid with an intense buttery flavor, has been extensively used in food chemistry [1,2]. Its vicinal dicarbonyl group provides it an attractive and very particular reactive behavior. Consequently, both **1a** and α-dicarbonyl compounds have been the object of a variety of pharmacological [3], phytochemical [4], photochemical [5], and synthetic studies [6,7]. For example, a series of α-dicarbonyl derivatives displays interesting fluorescent [8], structural [9], anti-corrosion [10], anti-inflammatory [11,12], antiprotozoal [13], or polymeric photoinitiator [14] properties. These derivatives can also be versatile substrates in the synthesis of a variety of compounds, including a large number of heterocycles [15,16,17,18,19,20], some building blocks (e.g., chiral α-hydroxy ketones [21,22]), and transition-metal ligands [23].

As part of an ongoing line of research, we used **1a** and α-dicarbonyl compounds as the key precursors in the synthesis of captodative alkenes [24] and *exo*-heterocyclic dienes [25]. The behavior of these relevant conjugated π-systems was experimentally and theoretically evaluated in Diels-Alder [24,25,26,27] and 1,3-dipolar [28,29,30] cycloadditions. Their scaffold was functionalized through Pd(0)-catalyzed coupling procedures [31] and used in the transformation of ligand-containing transition-metal complexes [32].

**Scheme 1:**
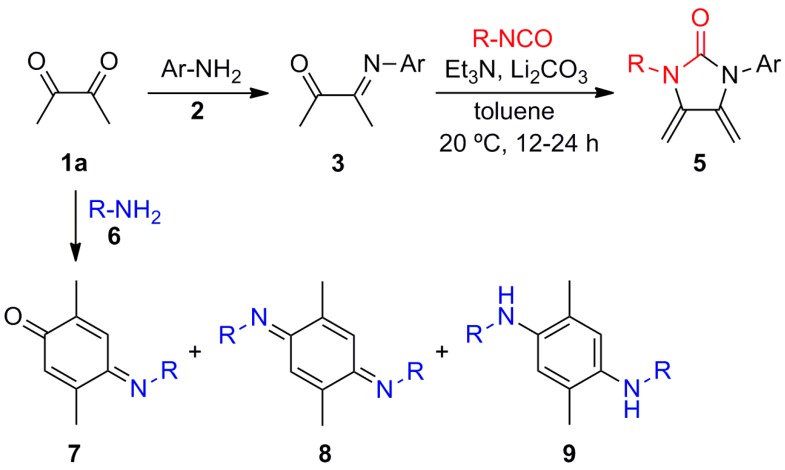
Reaction of diacetyl (**1a**) with anilines **2** and alkylamines **6**.

Recently, as a result of the synthesis of novel 2-imidazolidinone-base outer-ring *exo*-heterocyclic dienes **5** [27], we found that a base-assisted condensation/cyclization cascade reaction of monoimino diacetyl derivatives **3** and isocyanates in the presence of a dehydrating agent provided the desired dienes in high yields (Scheme 1). The monoimino diacetyl derivatives **3** were efficiently prepared by reacting **1a** with anilines **2** under neutral or Lewis acid catalysis conditions. However, derivatives **3** could not be prepared by using primary alkylamines. Only a limited number of old reports have described this kind of reaction, which exclusively yield brownish resins and oils, except for the thermochromic amber-yellow colored crystalline 2,5-dimethylbenzoquinone-*bis*-cyclohexyldiimine (**8b**) afforded by cyclohexylamine (**6b**) [33] (see below). All our attempts to purify these compounds by column chromatography over silica gel furnished decomposition resins.

Considering the limited scope of this reaction and the instability of the products, it is comprehensible that, to our best knowledge, no additional studies on this process have been reported. In spite of the drawbacks of an apparently disappointing and uninspiring reaction, we saw promise. With further investigation, in the mixture found of products **7**–**9**, the proportion depended on the reaction conditions. Therefore, we herein describe the efforts to gain insight into the limits and scope of this interesting reaction between diacetyl (**1a**) and alkylamines **6**.

## 2. Results and Discussion

### 2.1. Condensation of Diacetyl *(**1a**)* with Amines ***6***

Table 1 summarizes the reaction conditions of the process between **1a** and isopropylamine (**6a**). It appears that both the presence and proportion of two or three products depends not only on the number of mol equivalents of the amine, but also on the concentration of the reaction mixture (entries 1–4). Among the several solvents tested, methanol turned out to be the most efficient, though propanol could provide similar results albeit in lower yields (entry 5). The process yielded a larger proportion of iminoquinones **7a** and **8a** as well as their greater conversion when using twice the amount of **6a** and at high dilution (entry 4).

Interestingly, when hydroquinone was added to quench the probable formation of radical species, 1,4-diaminobenzene compound **9a** was the lone product (entry 6). This result suggests that the aromatization was readily performed under mild reductive conditions (see below). These three products could be separated as solids by flash column chromatography over 10% triethylamine pre-treated silica gel. However, if the crude mixture remained in the column for a long time, the yields decreased and many red and brown resin products were formed. Particular caution should be taken with products **7** and **8**, keeping them under refrigeration. Despite their instability, they can be handled at room temperature for further transformations.

**Table 1:** Optimization of reaction conditions for the preparation of compounds **7a**–**9a** ^a^. 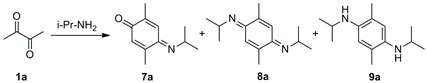

Entry	*i*-PrNH_2_ (6a) (mol equiv.)	Solvent (mmol/mL) ^b^	Additive (10% mol)	Products (%) ^c^
1	0.5	MeOH (0.46)	----------	**7a** (10), **9a** (13)
2	0.5	MeOH (1.14)	----------	**7a** (8), **8a** (2), **9a** (12)
3	1	MeOH (0.46)	----------	**7a** (12), **8a** (15), **9a** (40)
4	2	MeOH (0.23)	----------	**7a** (30), **8a** (21), **9a** (41)
5	0.5	*n*-PrOH (0.46)	----------	**7a** (5), **9a** (8)
6	2	MeOH (0.23)	hydroquinone	**9a** (68)

^a^ At room temperature for 48 h. ^b^ Concentration with respect to **1a** (11.44 mmol). ^c^ After column chromatography.

Analogous results were obtained when other α-branched primary amines were used (Table 2). Thus, cyclohexylamine (**6b**) reacted with **1a** under similar conditions to those used for **6a** to yield the expected three products **7b**/**8b**/**9b** (entry 1). Nevertheless, for (*S*)-methylbenzylamine (**6c**), the iminoquinone **7c** was not observed (entry 2).

**Table 2:** Condensation of **1a** with primary amines **6b**–**f** ^a^. 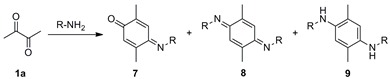

Entry	Amine	R	Products (%) ^b^
1	**6b**	cyclohexyl	**7b** (29), **8b** (23), **9b** (39)
2	**6c**	(*S*)-CH(Me)Ph	**7c** (0), **8c** (16), **9c** (30)
3	**6d**	*n*-butyl	**7d** (0), **8d** (0), **9d** (18)
4	**6e**	*n*-propyl	(c)
5	**6f**	Bn	(c)

^a^ With 2.0 mol equiv. of the amine in MeOH (0.23 mmol/mL) at room temperature for 48 h. ^b^ After column chromatography. ^c^ A complex mixture of products.

Also tested were primary *n*-alkylamines, such as *n*-propylamine (**6e**) and benzylamine (**6f**), obtaining a complex mixture of products (Table 2, entries 4–5). *n*-Butylamine (**6d**) afforded the corresponding 1,4-diaminobenzene **9d** in low yield (18%). These results could not be improved even when modifying the solvent, temperature and reaction times. Therefore, it appears that this kind of processes (leading to the formation of iminoquinones **7**–**9**) did not occur when primary amines were used, with the exception of *n*-butylamine that led to **9d** in low yield.

### 2.2. Functionalization of Iminoquinone ***7a***. Synthesis of Diarylamines and Polysubstituted Benzene Rings

**Figure 1:**
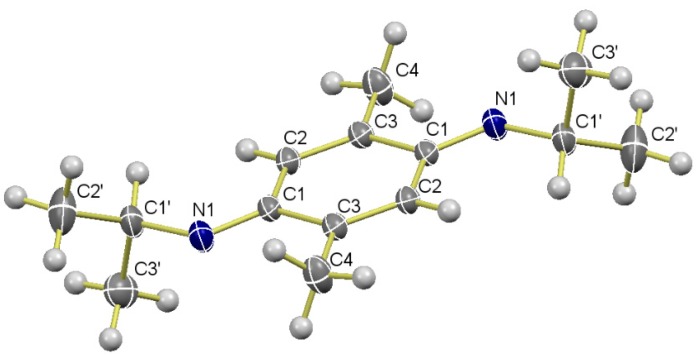
X-ray structure of **8a** (ellipsoids at the 30% probability level).

**Scheme 2:**
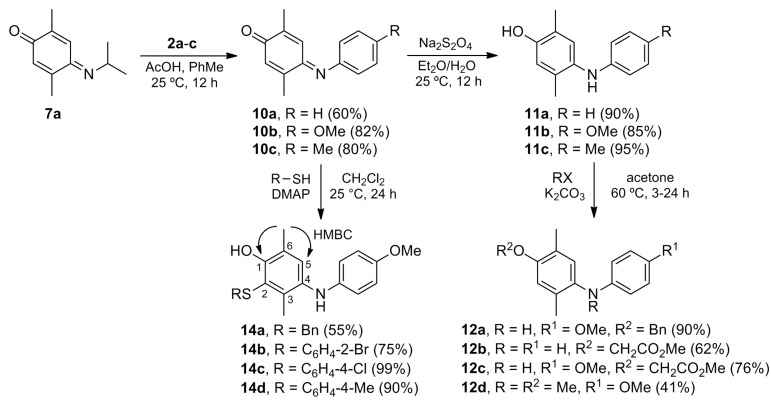
Conversion of **7a** into diarylamines **11**–**14**.

Iminoquinone **7a** underwent substitution at the isopropylimino moiety when it reacted with anilines **2a**–**c** to furnish iminoquinones **10a**–**c** in moderate to good yields (Scheme 2). Diverse studies have used the latter kind of molecules as intermediates for oxidative couplings of anilines with phenols to form dyes [34,35]. Iminoquinones have more recently resulted from oxidative bioconjugated couplings of anilines [36,37]. Interestingly, only the first of the two possible (*E*) and (*Z*) geometric isomers was obtained presently, which may be due to the destabilizing steric interactions generated in the (*Z*) isomer. The geometry was established by NOE experiments and single crystal X-ray diffraction of **8a** (Figure 1).

Diarylamines have become important synthetic targets as fine chemicals and precursors of a variety of *N*-containing pharmacological and natural products [38,39,40], such as carbazoles and ellipticines [41,42,43,44,45]. Due to the importance of diarylamines, a great number of synthetic approaches have been designed for their preparation [46,47,48]. One of the shortest and most efficient methods is through the Buchwald-Hartwig reaction, which consists of a Pd-catalyzed cross-coupling of aryl halides and anilines [49,50,51,52,53,54]. Another is the Ullmann reaction via a Cu-catalyzed coupling of similar substrates [55,56].

Consequently, we have investigated the conversion of iminoquinones **10a**–**c** into polysubstituted diarylamines **11**–**18**. Aromatization of iminoquinones **10a**–**c** under mild treatment with sodium hydrosulfite led to diarylamines **11a**–**c** in high yields (Scheme 2). The latter compounds were reacted with different alkyl halides in order to obtain the *O*- or *N*-alkyl derivatives **12a**–**d**. Accordingly, benzyl bromide (1.0 mol equiv.) afforded the *O*-benzylated diarylamine **12a** in high yield, while the reaction of **11a**–**b** with methyl bromoacetate led to phenoxyacetates **12b**–**c**, respectively, in good yields. When compound **11b** was submitted to methylation with methyl iodine (2.0 mol equiv.), the *O*,*N*-dimethyl diarylamine **12d** was yielded.

A further functionalization of iminoquinone **10b** was successfully accomplished by adding a series of thiols **13a**–**d**, furnishing the series of diarylamines **14a**–**d** in modest to good yields (Scheme 2). Thus, the aminophenolic ring became a pentasubstituted benzene ring. Although we expected that the most polarized enone-quinoid system of **10b** would be the most reactive site for the nucleophilic conjugated addition, the imino-quinoid moiety was the site at which the conjugated addition of thiols **13a**–**d** took place, followed by spontaneous aromatization. Although both alkyl- and arylthiols reacted efficiently, the latter furnished the desired products in higher yields.

This preference may be the result of favorable electronic interactions between both species. Presumably, thiophenol (a soft nucleophile) is selectively added to the conjugated imino-quinoid moiety, which should be softer than the enone moiety. The latter is highly polarized by the oxygen atom, mainly due to its electronegativity, which turns the enone system into a harder electrophile [57]. Although the results of similar studies support the importance of these electronic effects to explain this chemoselectivity [58], steric hindrance cannot be ruled out. The conjugated attack of the bulky thiophenol to the enone may be restrained by the presence of the vicinal anilino group, whose (*E*) configuration places the aryl ring on the same side of the unsubstituted enone carbon. In addition, this aryl ring adopts a slightly non-coplanar conformation with respect to the plane formed by the imino-quinoid ring [59,60], which may enhance such steric repulsion.

The structure of phenols **14a**–**d** was unambiguously established by 2D and NOE NMR experiments. The 2D HMBC showed a clear three-bond correlation between the protons of the CH_3_-C6 methyl group, both carbon atoms attached to the OH group (C-1), and the lone benzene proton (C-5). A similar correlation was observed between the protons of the CH_3_-C3 methyl group and both carbon atoms at the base of the thioether and anilino groups (C-2 and C-4). Due to the close chemical shifts of the aromatic proton signals (selectively impeding irradiation as well as the ability to observe the corresponding signal enhancements), the NOE experiments were carried out with the *O*-allyl derivative of **14c** (see compound **17c**).

**Scheme 3:**
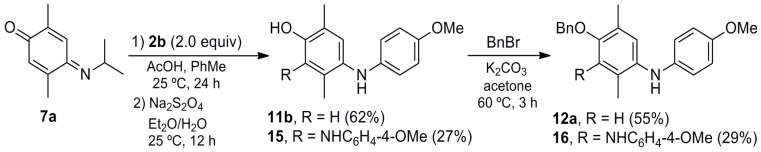
Conversion of **7a** into diarylamines **15** and **16**.

Taking into account the feasible nucleophilic conjugated addition of thiols **13** to **10b**, anilines **2** were considered as potential nucleophiles. Therefore, the most nucleophilic *p*-anisidine (**2b**) was added to **10b**, but no addition product was detected. In spite of this result, a trial was carried out starting from **7a** and in the presence of an excess (2.0 mol equiv.) of **2b**, followed by the reduction treatment, resulting in a mixture of **11b** as the major product and the desired adduct **15** as the minor one (Scheme 3). Due to the difficulty of purifying the latter, the benzyl diarylamine **16** was generated (with compound **12a** as the major product) in a one-pot procedure without the isolation of **15**. It is worth mentioning that these reactions followed the same chemoselective addition pathway to the imino quinoid system as the thiols **13a**–**d**. The low yields of products **15** and **16**, and the fact that other less activated anilines were unable to give the double addition, may be explained by the lower nucleophilicity of anilines with respect to thiols. Also unsuccessful was the conjugated addition of soft nucleophiles, such as dimethyl malonate or nitromethane carbanions to iminoquinone **7a** or anilinoquinone **10b**, that led to the recovery of the starting materials.

**Scheme 4:**
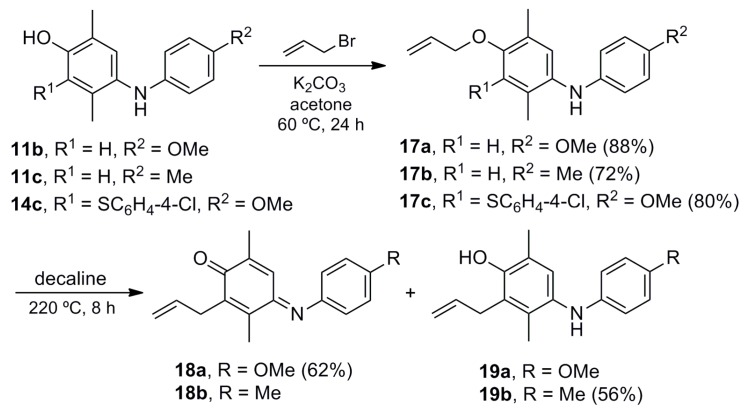
Synthesis of allyl aryl ethers **17a**–**c** and their Claisen rearrangement.

In order to increase the number of substituents with valuable functional groups on the phenol ring, we investigated the allylation of phenols **11b**–**c** and **14c** and subsequent Claisen rearrangement (Scheme 4). The first reaction proceeded efficiently to give the corresponding allyl ethers **17a**–**c** in high yields. Derivative **17b** was submitted to the thermal Claisen rearrangement to furnish the expected [61] pentasubstituted allyl phenol **19b** in a modest yield, observing the starting material and decomposition by-products. However, iminoquinone **18a** was the main product in the case of **17a**, found along with the starting material and by-products (phenol **19a** was not isolated). The electron-demand of the *para* substituent (anilino group) in precursors **17a**–**b** is presumably involved in this unexpected selectivity [62].

The thioaryl analogue **17c** was used in NOE experiments to support the HMBC assignment of the structures of derivatives **14a**–**d**. The irradiation of the signal attributed to the methylene group of the allyl moiety generated a selective enhancement of the signal assigned to the *ortho* (with respect to the sulfur atom) aromatic protons of the thioether group. This result, along with that of other NOE experiments, confirmed that the addition of the thiophenols **13a**–**d** to iminoquinone **10b** took place at the imino-quinoid moiety.

### 2.3. Functionalization of bis-Iminoquinone ***8a*** Synthesis of Amino-Diarylamines and bis-Diarylamines

**Scheme 5:**
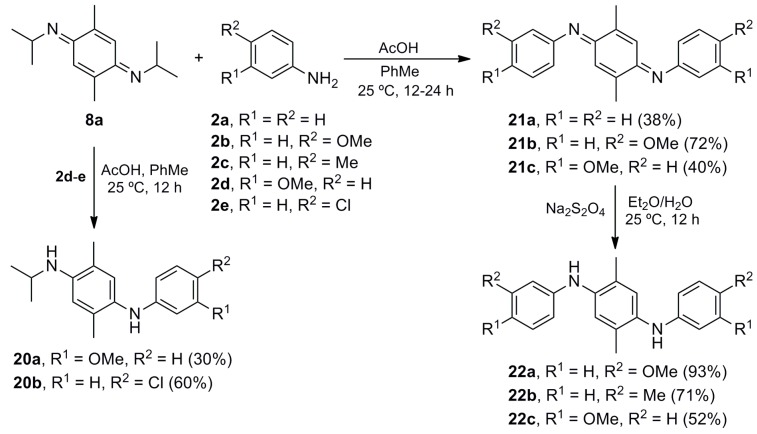
Conversion of *bis*-imine **8a** into aniline derivatives **20**–**22**.

Since the substitution of the isopropylamino group in iminoquinone **7a** by anilines **2a**–**c** proceeded to give iminoquinones **10a**–**c**, it was considered that *bis*-iminoquinone **8a** could possibly undergo a *mono*- or *bis*-substitution by anilines **2a**–**e** (Scheme 5). Indeed, the addition of 1.0 mol equiv of deactivated anilines **2d**–**e** to *bis*-iminoquinone **8a** resulted in the formation of *p*-aminodiarylamines **20a**–**b** in low to modest yields. On the other hand, the addition of an excess of anilines **2a**–**b** and **2d** produced a double substitution of the two isopropylamino groups by the aniline nucleophiles leading to corresponding *bis*-iminoquinones **21a**–**c**. The most activated *p*-anisidine (**2b**) was the most efficient aniline, while anilines **2a** and **2d** gave rise to the corresponding *bis*-iminoquinones **21a** and **21c** in lower yields, recovering the starting materials and side-products after 24 h of reaction (a longer reaction time afforded traces of the desired products and a deep red resin residue). This behavior is probably due to the lower nucleophilicity of these anilines, an idea supported by the fact that the double substitution did not take place when **8a** was submitted to the addition of the deactivated aniline **2e**.

Unlike *bis*-iminoquinone **8a**, which suffers decomposition after remaining several hours at room temperature, *bis*-iminoquinones **21** are stable red oils or solids under the same conditions. The color of these compounds become yellow in methanol, methylene chloride or toluene solutions, as previously observed for **8b** [33].

The reduction of **21b** by treatment with sodium hydrosulfite provided *bis*-diarylamine **22a** in high yield (Scheme 5). A one-pot reaction was also tried for the addition of anilines **2c** and **2d** to **8a**, followed by reduction with sodium hydrosulfite, affording *bis*-diarylamines **22b**–**c**, respectively. These *bis*-iminoquinones **21** and 1,4-phenylenediamines **22** are of significant importance as conducting polymers [63] and as efficient substrates in electron-transfer [64] and electrochemical studies [65].

The structure of these compounds was established by spectrometric analyses and X-ray diffraction. Figure 1 shows the X-ray structure of **8a**, in which both imino moieties display an (*E*) configuration. Similarly, NOE experiments showed that *bis*-iminoquinones **21** possess (*E*,*E*) configurations, which is in agreement with previous X-ray diffraction evidence of compound **21a** [59] and the *bis*-iminoquinone derived from **2c** [60].

### 2.4. Mechanism of Formation of Compounds ***7**–**9***

**Scheme 6:**
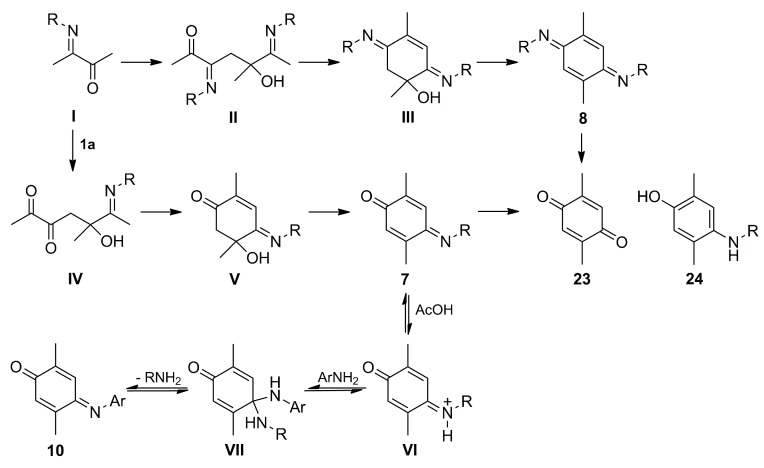
Proposed reaction mechanism for the formation of iminoquinones **7** and **8**, and transamination process from **7** to **10**.

The proposed mechanism for the formation of compounds **7**–**9** is depicted in Scheme 6. As proposed by Carlson [33], diacetyl (**1a**) reacts with the amine to give rise to imino ketone **I**, which undergoes auto condensation to generate the imino aldol intermediate **II**. This is cyclized through an internal condensation followed by a loss of water to afford **III** (an intermediate isolated by Carlson for the case of **6b**, but never observed or isolated in our trials), which leads to the isolation of *bis*-iminoquinones **8** after losing another molecule of water. These compounds undergo reduction to compounds **9** in the middle of the reaction. The latter conversion also takes place when leaving a methanolic solution of **8a** at room temperature, which rapidly changes from yellow to a dark color, observing a mixture of **9a** with a dark red resin.

Under standardized reaction conditions (Table 1 and Table 2), this process seems to be faster than that of the formation of iminoquinones **7**, as observed by *tlc* and ^1^H-NMR. Compounds **8** before compounds **7**, suggesting that the latter are formed via an independent pathway or through mono-hydrolysis of **8**. The second hypothesis is not feasible, because there was no evidence of the formation of **7a** when a solution of **8a** remained for a long time under the same reaction conditions as those used for the synthesis of both compounds, leading rather to compound **9a** and side-products. Therefore, it is presumed that iminoquinones **7** are formed via a pathway that includes the aldol condensation of **I** with another molecule of **1a** to generate aldol **IV**, which by intramolecular aldolization yields intermediate **V**. Finally, products **7** are formed by the loss of a water molecule. This hypothesis is supported by the trials displayed in Table 1 (entries 1 and 5), in which only products **7a** and **9a** were generated by the presence of an excess of **1a** in the middle of the reaction.

Interestingly, quinone **23** was only isolated in traces from the reaction mixture. It is well known that this quinone results from the condensation of two molecules of diacetyl (**1a**) under basic conditions [33]. However, we have isolated it as the main product from the reaction between **1a** and **2a** after purification of the crude mixture by column chromatography using silica gel not pre-treated with triethylamine. This suggests that **23** proceeds from imines **7** and **8** by hydrolysis during the purification process, but not from the reaction.

Another interesting case is that of phenol **24**, which was not observed or detected in the reaction mixture by NMR, suggesting that iminoquinones **7** are stable enough to undergo reduction under the reaction conditions. This is in contrast with compounds **8**, in which the reductive aromatization takes place during the reaction or the purification process to yield *p*-dianilinobenzenes **9**.

Regarding the transamination process from the iminoquinone **7a** to **10a**–**c** and from diiminoquinone **8a** to **21a**–**c**, the mechanism can be explained in terms of a series of equilibria promoted by the Brønsted acid catalyst (AcOH) in the presence of anilines **2**, as summarized in Scheme 6. It is likely that the first acid-base equilibrium is established between **7** and the amino protonated species **VI**, and that this undergoes the attack of aniline **2** (which is more nucleophilic and less basic than the alkyl amine) to generate aminal species **VII**, followed by an elimination of the most basic amine (isopropylamine) to provide the observable aryliminoquinones **10**. Additionally, this equilibrium seems to be favored by the higher stability of **10** than alkyliminoquinones **7**, resulting from a more stable conjugated imino system. These arguments can also be applied to the transamination from **8a** into *bis*-iminoquinones **21**.

## 3. Experimental Section

### 3.1. General

Melting points were determined with a capillary melting point apparatus. IR spectra were recorded on a Perkin-Elmer 2000 spectrophotometer (PerkinElmer, Waltham, MA, USA). ^1^H (300 or 500 MHz) and ^13^C (75 or 125 MHz) NMR spectra were recorded on Varian Mercury-300 (Varian, Inc., Palo Alto, CA, USA), and Varian VNMR System instruments (Varian, Inc., Palo Alto, CA, USA), with TMS as internal standard; chemical shifts (δ) are reported in ppm. Assignment of the NMR signals was made by HMQC and HMBC 2D methods (for the ^1^H- and ^13^C-NMR spectra of the new compounds, see the supplementary figures). Mass spectra (MS) and high-resolution mass spectra (HRMS) were obtained in the electron impact (EI) (70 eV) mode and recorded on Polaris Q-Trace GC Ultra (Finnigan Co., Waltham, MA, USA) and Jeol JSM-GCMateII apparatuses (JEOL, Ltd., Tokyo, Japan), respectively. Elemental analyses were performed on a CE-440 Exeter Analytical instrument (Exeter Analytical, Inc., North Chelmsford, MA, USA), X-ray crystallographic measurements were collected on an Oxford XcaliburS diffractometer (Rigaku Co., Tokyo, Japan). Analytical thin-layer chromatography was carried out using E. Merck silica gel 60 F254 coated 0.25 plates, visualized by a long- and short-wavelength UV lamp. Flash column chromatography was performed over Natland International Co. (Morrisville, NC, USA) silica gel (230–400 mesh) and silica gel (230–400 mesh) pre-treated with trimethylamine (10%). All air moisture sensitive reactions were carried out under nitrogen using oven-dried glassware. MeOH, and toluene were freshly distilled over sodium, as well as methylene chloride over calcium hydride, prior to use. Acetone was dried by distillation after treatment with 4 Å molecular sieves. K_2_CO_3_ was dried overnight at 120 °C prior to use. Triethylamine was freshly distilled from NaOH. All other reagents were used without further purification.

### 3.2. Synthesis and Characterization

*(E)-4-(Isopropylimino)-2,5-dimethylcyclohexa-2,5-dien-1-one* (**7a**), *(E,E)-N,N′-Diisopropyl-2,5-dimethylcyclohexa-2,5-diene-1,4-diimine* (**8a**) *and N,N′-Diisopropyl-2,5-dimethylbenzene-1,4-diamine* (**9a**). A mixture of 2,3-butanedione (**1a**) (1.971 g, 22.91 mmol) and isopropylamine (**6a**) (2.707 g, 45.88 mmol) in MeOH (400 mL) was stirred at room temperature for 48 h. The crude reaction mixture was concentrated under vacuum, and the residue was purified by column chromatography over silica gel impregnated with triethylamine (10%) in hexane (40 g/g of crude, hexane) to give **7a** (0.610 g, 30%) as a pale green-yellow solid, **8a** (0.524 g, 21%) as a dark red solid, and **9a** (1.04 g, 41%) as a red solid.

Data of **7a**: R*_f_* 0.53 (hexane/EtOAc, 8:2); mp 64–65 °C. IR (film): ν_max_ 2969, 2925, 1652, 1631, 1604, 1518, 1461, 1376, 1256, 1169, 894 cm^−1^. ^1^H-NMR (500 MHz, CDCl_3_): δ = 1.25 (d, *J* = 6.0 Hz, 6H, (C*H*_3_)_2_CH), 2.00 (d, *J* = 1.5 Hz, 3H, C*H*_3_-C2), 2.15 (d, *J* = 1.5 Hz, 3H, C*H*_3_-C5), 4.18 (sept, *J* = 6.5 Hz, 1H, (CH_3_)_2_C*H*), 6.41 (q, *J* = 1.5 Hz, 1H, H-6), 7.10 (dq, *J* = 1.5, 0.5 Hz, 1H, H-3). ^13^C-NMR (125 MHz, CDCl_3_): δ = 16.1 (*C*H_3_-C2), 17.7 (*C*H_3_-C5), 24.2 ((*C*H_3_)_2_CH), 51.8 ((CH_3_)_2_*C*H), 122.2 (C-3), 129.3 (C-6), 140.0 (C-2), 150.9 (C-5), 156.1 (C-4), 188.3 (C-1). MS (70 eV): *m/z* (%) 177 (M^+^, 100), 162 (73), 149 (45), 134 (58), 117 (43), 106 (35), 91 (52), 77 (19). Anal. calcd for C_11_H_15_NO: C, 74.54; H, 8.53; N, 7.90. Found: C, 74.56; H, 8.49; N, 7.94.

Data of **8a**: R*_f_* 0.64 (hexane/EtOAc, 8:2); mp 94–95 °C. IR (film): ν_max_ 2967, 2924, 1599, 1581, 1519, 1377, 1359, 1345, 1258, 1116, 875 cm^−1^. ^1^H-NMR (500 MHz, CDCl_3_): δ = 1.20 (d, *J* = 6.0 Hz, 6H, (C*H*_3_)_2_CH), 2.00 (d, *J* = 1.5 Hz, 6H, C*H*_3_-C2, C*H*_3_-C5), 4.08 (sept, *J* = 6.0 Hz, 2H, (CH_3_)_2_C*H*), 6.79 (br d, *J* = 1.5 Hz, 2H, H-3, H-6). ^13^C-NMR (125 MHz, CDCl_3_): δ = 18.5 (*C*H_3_-C2, *C*H_3_-C5), 24.2 (2(*C*H_3_)_2_CH), 50.4 (2(CH_3_)_2_*C*H), 118.4 (C-3, C-6), 143.3 (C-2, C-5), 156.9 (C-1, C-4). MS (70 eV): *m/z* (%) 218 (M^+^, 20), 203 (95), 161 (34), 146 (100), 145 (43), 132 (20), 117 (6), 91 (7). Anal. calcd for C_14_H_22_N_2_: C, 77.01; H, 10.16; N, 12.83. Found: C, 77.00; H, 10.21; N, 12.78.

Data of **9a**: R*_f_* 0.22 (hexane/EtOAc, 8:2); mp 53–54 °C. IR (film): ν_max_ 3382, 2965, 2928, 1520, 1463, 1413, 1381, 1218, 1166, 1125, 1004, 857 cm^−1^. ^1^H-NMR (500 MHz, CDCl_3_): δ = 1.19 (d, *J* = 6.5 Hz, 6H, (C*H*_3_)_2_CH), 2.10 (s, 6H, C*H*_3_-C2, C*H*_3_-C5), 2.70 (br s, 2H, NH), 3.53 (sept, *J* = 6.5 Hz, 2H, (CH_3_)_2_C*H*), 6.46 (s, 2H, H-3, H-6). ^13^C-NMR (125 MHz, CDCl_3_): δ = 17.6 (*C*H_3_-C2, *C*H_3_-C5), 23.5 (2(*C*H_3_)_2_CH), 45.4 (2(CH_3_)_2_*C*H), 115.6 (C-3, C-6), 121.7 (C-2, C-5), 137.3 (C-1, C-4). MS (70 eV): *m/z* (%) 220 (M^+^, 98), 205 (100), 177 (99), 148 (20), 135 (56), 95 (15), 75 (6). HRMS (EI): *m/z* [M^+^] calcd for C_14_H_24_N_2_: 220.1940; found: 220.1938.

*(E)-4-(Cyclohexylimino)-2,5-dimethylcyclohexa-2,5-dien-1-one* (**7b**), *(E,E)-N,N′-Dicyclohexyl-2,5-dimethylcyclohexa-2,5-diene-1,4-diimine* (**8b**) and *N,N′-Dicyclohexyl-2,5-dimethylbenzene-1,4-diamine* (**9b**). The procedure for the preparation of **7a**–**9a** was followed using a mixture of **1a** (1.971 g, 22.91 mmol) and cyclohexylamine (**6b**) (4.53 g, 45.8 mmol) in MeOH (400 mL) to give **7b** (0.721 g, 29%) as a pale green-yellow solid, **8b** (0.784 g, 23%) as a yellow solid, and **9b** (1.34 g, 39%) as a dark brown solid.

Data of **7b**: R*_f_* 0.51 (hexane/EtOAc, 9:1); mp 63–64 °C. IR (film): ν_max_ 2925, 2853, 1647, 1620, 1370, 1268, 1161, 900 cm^−1^. ^1^H-NMR (500 MHz, CDCl_3_): δ = 1.33 (qt, *J* = 12.0, 3.3 Hz, 1H, H-Cy), 1.43 (qt, *J* = 12.0, 3.3 Hz, 2H, H-Cy), 1.55–1.62 (m, 2H, H-Cy), 1.64–1.72 (m, 3H, H-Cy), 1.82–1.87 (m, 2H, H-Cy), 2.01 (d, *J* = 1.5 Hz, 3H, C*H*_3_-C2), 2.14 (d, *J* = 1.5 Hz, 3H, C*H*_3_-C5), 3.80–3.87 (m, 1H, NCH-Cy), 6.41 (br s, 1H, H-6), 7.08 (br s, *J* = 1.5 Hz, 1H, H-3). ^13^C-NMR (125 MHz, CDCl_3_): δ = 16.1 (*C*H_3_-C2), 17.7 (*C*H_3_-C5), 24.3 (2C-3′), 25.6 (C-4′), 34.3 (2C-2′), 60.3 (C-1′), 122.3 (C-3), 129.3 (C-6), 139.9 (C-2), 150.9 (C-5), 156.3 (C-4), 188.4 (C-1). HRMS (EI): *m/z* [M^+^] calcd for C_14_H_19_NO: 217.1467; found: 217.1470.

Data of **8b**: R*_f_* 0.68 (hexane/EtOAc, 9:1); mp 147–148 °C [Lit. [33] 145.6–147 °C]. IR (film): ν_max_ 2923, 2850, 1598, 1575, 1454, 1351, 1166, 962, 889, 871 cm^−1^. ^1^H-NMR (500 MHz, CDCl_3_): δ = 1.28 (qt, *J* = 13.0, 2.5 Hz, 2H, H-Cy), 1.35 (qt, *J* = 13.0, 2.5 Hz, 4H, H-Cy), 1.40–1.48 (m, 4H, H-Cy), 1.56–1.61 (m, 6H, H-Cy), 1.72–1.79 (m, 4H, H-Cy), 2.02 (s, 6H, C*H*_3_-C2, C*H*_3_-C5), 3.61–3.68 (m, 2H, NCH-Cy), 6.70 (br s, 2H, H-3, H-6). ^13^C-NMR (125 MHz, CDCl_3_): δ = 18.4 (*C*H_3_-C2, *C*H_3_-C5), 24.7 (4C-3′), 25.8 (2C-4′), 34.2 (4C-2′), 59.0 (2C-1′), 118.5 (C-3, C-6), 143.3 (C-2, C-5), 157.1 (C-1, C-4). HRMS (EI): *m/z* [M^+^] calcd for C_20_H_30_N_2_: 298.2409; found: 298.2402.

Data of **9b**: R*_f_* 0.21 (hexane/EtOAc, 9:1); mp 108–109 °C. IR (film): ν_max_ 3383, 2927, 2850, 1519, 1445, 1412, 1211, 1107, 850 cm^−1^. ^1^H-NMR (300 MHz, CDCl_3_): δ = 1.06–1.44 (m, 10H, H-Cy), 1.57–1.67 (m, 2H, H-Cy), 1.70–1.80 (m, 4H, H-Cy), 2.00–2.08 (m, 2H, H-Cy), 2.10 (s, 6H, C*H*_3_-C2, C*H*_3_-C5), 2.76 (br s, 2H, NH), 3.08–3.20 (m, 2Hz, NCH-Cy), 6.45 (s, 2H, H-3, H-6). ^13^C-NMR (75 MHz, CDCl_3_): δ = 17.7 (*C*H_3_-C2, *C*H_3_-C5), 25.1 (4C-3′), 26.0 (2C-4′), 34.0 (4C-2′), 53.0 (2C-1′), 115.4 (C-3, C-6), 121.5 (C-2, C-5), 137.0 (C-1, C-4). HRMS (EI): *m/z* [M^+^] calcd for C_20_H_32_N_2_: 300.2566; found: 300.2570.

*(E,E)-2,5-Dimethyl-N,N′-bis((S)-1-phenylethyl)cyclohexa-2,5-diene-1,4-diimine* (**8c**) and *2,5-Dimethyl-N,N′-bis((S)-1-phenylethyl)benzene-1,4-diamine* (**9c**). The procedure for the preparation of **7a**–**9a** was followed using a mixture of **1a** (0.098 g, 1.14 mmol) and (*S*)-phenylethylamine (**6c**) (0.278 g, 2.3 mmol) in MeOH (10 mL) to give **8c** (0.031 g, 16%) as a yellow solid and **9c** (0.059 g, 30%) as a dark brown oil.

Data of **8c**: R*_f_* 0.65 (hexane/EtOAc, 8:2); mp 108–109 °C; ${\left[\text{α}\right]}_{D}^{22}$ = −44.28 (*c* 0.473, CHCl_3_). IR (film): ν_max_ 1699, 1493, 1450, 1354, 1125, 760, 699 cm^−1^. ^1^H-NMR (500 MHz, CDCl_3_): δ = 1.49 (d, *J* = 6.5 Hz, 6H, C*H*_3_CH), 2.17 (d, *J* = 1.5 Hz, 6H, C*H*_3_-C2, C*H*_3_-C5), 5.08 (q, *J* = 6.5 Hz, 2H, CH_3_C*H*), 6.89 (br s, 1H, H-3, H-6), 7.19–7.24 (m, 2H, H-4′′), 7.30–7.34 (m, 4H, H-3′′), 7.43–7.46 (m, 4H, H-2′′). ^13^C-NMR (125 MHz, CDCl_3_): δ = 18.5 (*C*H_3_-C2, *C*H_3_-C5), 25.7 (2*C*H_3_CH), 58.9 (2CH_3_*C*H), 118.5 (C-3, C-6), 126.58 (2C-4′′), 126.61 (4C-2′′), 128.4 (4C-3′′), 144.1 (C-2, C-5), 146.2 (2C-1′′), 157.5 (C-1, C-4). MS (70 eV): *m/z* (%) 342 (M^+^, 22), 328 (23), 327 (100), 300 (14), 237 (12), 222 (24), 208 (26), 105 (20), 97 (24), 86 (38), 71 (43), 57 (45). HRMS (EI): *m/z* [M^+^] calcd for C_24_H_26_N_2_: 342.2096; found: 342.2090.

Data of **9c**: R*_f_* 0.32 (hexane/EtOAc, 9:1); ${\left[\text{α}\right]}_{D}^{22}$ = +10.75 (*c* 0.282, CHCl_3_). IR (film): ν_max_ 3417, 2969, 1448, 1414, 1520, 1371, 1224, 769, 698 cm^−1^. ^1^H-NMR (300 MHz, CDCl_3_): δ = 1.47 (d, *J* = 6.6 Hz, 6H, C*H*_3_CH), 2.02 (s, 6H, C*H*_3_-C2, C*H*_3_-C5), 3.30 (br s, 2H, NH), 4.39 (q, *J* = 6.6 Hz, 2H, CH_3_C*H*), 6.21 (s, 2H, H-3, H-6), 7.22–7.37 (m, 10H, PhH). ^13^C-NMR (75 MHz, CDCl_3_): δ = 17.6 (2*C*H_3_Ar), 25.1 (2*C*H_3_CH), 54.0 (2CH_3_*C*H), 114.7 (C-3, C-6), 120.6 (C-2, C-5), 125.8 (4C-2′′), 126.5 (2C-4′′), 128.4 (4C-3′′), 136.9 (C-1, C-4), 145.9 (2C-1ʹʹ). HRMS (EI): *m/z* [M^+^] calcd for C_24_H_28_N_2_: 344.2253; found: 344.2257.

*N,N′-Dibutyl-2,5-dimethylbenzene-1,4-diamine* (**9d**). The procedure for the preparation of **7a**–**9a** was followed using a mixture of **1a** (1.971 g, 22.91 mmol) and *n*-butylamine (**6d**) (3.34 g, 45.8 mmol) in MeOH (400 mL) to give **9d** (0.512 g, 18%) as a dark brown oil. R*_f_* 0.44 (hexane/EtOAc, 8:2). IR (film): ν_max_ 3369, 2955, 2925, 1518, 1469, 1413, 1222, 1214, 994, 855, 742 cm^−1^. ^1^H-NMR (500 MHz, CDCl_3_): δ = 0.96 (t, *J* = 7.5 Hz, 6H, N(CH_2_)_3_C*H*_3_), 1.44 (sext, *J* = 7.5 Hz, 4H, N(CH_2_)_2_C*H*_2_CH_3_), 1.62 (qu, *J* = 7.0 Hz, 4H, NCH_2_C*H*_2_CH_2_CH_3_), 2.12 (s, 6H, C*H*_3_-C2, C*H*_3_-C5), 2.98 (br s, 2H, NH), 3.08 (br t, *J* = 7.0 Hz, 4H, NC*H*_2_(CH_2_)_2_CH_3_), 6.44 (s, 2H, H-3, H-6). ^13^C-NMR (125 MHz, CDCl_3_): δ = 13.9 (2N(CH_2_)_3_*C*H_3_), 17.4 (*C*H_3_-C2, *C*H_3_-C5), 20.4 (2N(CH_2_)_2_*C*H_2_CH_3_), 32.0 (2NCH_2_*C*H_2_CH_2_CH_3_), 45.0 (2N*C*H_2_(CH_2_)_2_CH_3_), 114.1 (C-3, C-6), 121.1 (C-2, C-5), 138.3 (C-1, C-4). MS (70 eV): *m/z* (%) 248 (M^+^, 70), 205 (100), 191 (32), 135 (10), 120 (11), 81 (26). HRMS (EI): *m/z* [M^+^] calcd for C_16_H_28_N_2_: 248.2253; found: 248.2260.

*(E)-2,5-Dimethyl-4-(phenylimino)cyclohexa-2,5-dien-1-one* (**10a**). A mixture of **7a** (0.442 g, 2.50 mmol) and aniline (**2a**) (0.233 g, 2.50 mmol) in toluene (40 mL) was stirred at room temperature for 10 min. Then, glacial acetic acid (0.524 g, 8.74 mmol) was added dropwise and the mixture was stirred at room temperature for 12 h. The crude mixture was concentrated under vacuum and purified by column chromatography over silica gel (10 g/g crude, hexane/EtOAc, 8:2) to give **10a** (0.316 g, 60%) as a dark red solid. R*_f_* 0.66 (hexane/EtOAc, 8:2); mp 73–74 °C. IR (film): ν_max_ 1649, 1633, 1603, 1497, 1482, 1262, 1204, 1159, 900, 762, 697 cm^−1^. ^1^H-NMR (300 MHz, CDCl_3_): δ = 1.93 (d, *J* = 1.5 Hz, 3H, C*H*_3_-C2), 2.27 (d, *J* = 1.5 Hz, 3H, C*H*_3_-C5), 6.55 (q, *J* = 1.5 Hz, 1H, H-6), 6.78 (q, *J* = 1.5 Hz, 1H, H-3), 6.80–6.85 (m, 2H, H-2′), 7.17-7.22 (m, 1H, H-4′), 7.34–7.44 (m, 2H, H-3′). ^13^C-NMR (75 MHz, CDCl_3_): δ = 16.1 (*C*H_3_-C2), 18.0 (*C*H_3_-C5), 120.2 (C-2′), 125.4 (C-4′), 125.6 (C-3), 129.2 (C-3′), 131.1 (C-6), 141.5 (C-2), 149.5 (C-4), 150.2 (C-1′), 158.5 (C-5), 188.4 (C-1). HRMS (EI): *m/z* [M^+^] calcd for C_14_H_13_NO: 211.0997; found: 211.0989.

*(E)-4-((4-Methoxyphenyl)imino)-2,5-dimethylcyclohexa-2,5-dien-1*-one (**10b**). The procedure for the preparation of **10a** was followed using a mixture of **7a** (0.202 g, 1.14 mmol) and 4-methoxyaniline (**2b**) (0.140 g, 1.14 mmol) and glacial acetic acid (0.524 g, 8.74 mmol) in toluene (20 mL) to give **10b** (0.227 g, 82%) as a dark red solid. R*_f_* 0.40 (hexane/EtOAc, 1:1); mp 79–80 °C. IR (film): ν_max_ 1648, 1628, 1599, 1499, 1246, 1164, 1033, 902, 843, 762 cm^−1^. ^1^H-NMR (500 MHz, CDCl_3_): δ = 1.95 (d, *J* = 1.5 Hz, 3H, C*H*_3_-C2), 2.26 (d, *J* = 1.5 Hz, 3H, C*H*_3_-C5), 3.84 (s, 3H, C*H*_3_O), 6.52 (br d, *J* = 1.5 Hz, 1H, H-6), 6.83–6.86 (m, 2H, H-2′), 6.90 (br d, *J* = 1.5 Hz, 1H, H-3), 6.93–6.97 (m, 2H, H-3′). ^13^C-NMR (125 MHz, CDCl_3_): δ = 15.8 (*C*H_3_-C2), 17.7 (*C*H_3_-C5), 55.4 (*C*H_3_O), 114.2 (C-3′), 122.4 (C-2′), 125.3 (C-3), 130.4 (C-6), 140.7 (C-2), 143.1 (C-1′), 149.4 (C-5), 157.7 (C-4), 157.9 (C-4′), 188.1 (C-1). MS (70 eV): *m/z* (%) 241 (M^+^, 70), 226 (36), 210 (100), 198 (22), 182 (40), 167 (26), 155 (18). HRMS (EI): *m/z* [M^+^] calcd for C_15_H_15_NO_2_: 241.1103; found: 241.1090.

*(E)-2,5-Dimethyl-4-(p-tolylimino)cyclohexa-2,5-dien-1-one* (**10c**). The procedure of preparation for **10a** was followed using a mixture of **7a** (0.300 g, 1.69 mmol), 4-methylaniline (**2c**) (0.208 g, 1.69 mmol) and glacial acetic acid (0.524 g, 8.73 mmol) in toluene (20 mL) to give **10c** (0.305 g, 80%) as a dark red solid. R*_f_* 0.68 (hexane/EtOAc, 7:3); mp 88–89 °C. IR (film): ν_max_ 2922, 1628, 1508, 1446, 1259, 1110, 1091, 1006, 903, 842, 804 cm^−1^. ^1^H-NMR (300 MHz, CDCl_3_): δ = 1.93 (d, *J* = 1.4 Hz, 3H, C*H*_3_-C2), 2.27 (d, *J* = 1.4 Hz, 3H, C*H*_3_-C5), 2.38 (s, 3H, C*H*_3_Ar), 6.53 (q, *J* = 1.4 Hz, 1H, H-6), 6.72–6.78 (m, 2H, H-2′), 6.84 (q, *J* = 1.4 Hz, 1H, H-3), 7.17–7.24 (m, 2H, H-3′). ^13^C-NMR (75 MHz, CDCl_3_): δ = 15.8 (*C*H_3_-C2), 17.8 (*C*H_3_-C5), 20.8 (*C*H_3_Ar), 120.3 (C-2′), 125.4 (C-3), 129.5 (C-3′), 130.6 (C-6), 135.1 (C-4′), 140.9 (C-2), 147.4 (C-1′), 149.4 (C-5), 158.1 (C-4), 188.3 (C-1). HRMS (EI): *m/z* [M^+^] calcd for C_15_H_15_NO: 225.1154; found: 225.1155.

*2,5-Dimethyl-4-(phenylamino)phenol* (**11a**). To a solution of **10a** (0.100 g, 0.47 mmol) in Et_2_O (10 mL) a saturated aqueous solution of sodium hydrosulfite (30 mL) was added, and the mixture was stirred at room temperature for 12 h. The crude mixture was washed with CH_2_Cl_2_ (3 × 10 mL) and the organic layer was dried (Na_2_SO_4_), concentrated under vacuum and purified by column chromatography over silica gel (10 g/g crude, hexane/EtOAc, 9:1) to give **11a** (0.090 g, 90%) as a pink oil. R*_f_* 0.49 (hexane/EtOAc, 8:2). IR (film): ν_max_ 3382, 2923, 1600, 1497, 1462, 1407, 1200, 864, 745, 694 cm^−1^. ^1^H-NMR (300 MHz, CDCl_3_): δ = 2.14 (s, 3H, C*H*_3_-C5), 2.18 (s, 3H, C*H*_3_-C2), 4.50–5.30 (br, 2H, NH, OH), 6.65 (s, 1H, H-6), 6.66–6.73 (m, 2H, H-2′), 6.77 (t, *J* = 7.3 Hz, 1H, H-4′), 6.98 (s, 1H, H-3), 7.14–7.22 (m, 2H, H-3′). ^13^C-NMR (75 MHz, CDCl_3_): δ = 15.4 (*C*H_3_-C2), 17.5 (*C*H_3_-C5), 114.6 (C-2′), 117.1 (C-6), 118.6 (C-4′), 121.9 (C-2), 126.8 (C-3), 129.2 (C-3′), 131.6 (C-5), 133.1 (C-4), 146.4 (C-1′), 150.4 (C-1). HRMS (EI): *m/z* [M^+^] calcd for C_14_H_15_NO: 213.1154; found: 213.1144.

*4-((4-Methoxyphenyl)amino)-2,5-dimethylphenol* (**11b**). The procedure for the preparation of **11a** was followed using a mixture of **10b** (0.099 g, 0.41 mmol) in Et_2_O (10 mL) and a saturated aqueous solution of sodium hydrosulfite (30 mL) to give **11b** (0.085 g, 85%) as a red solid. R*_f_* 0.25 (hexane/EtOAc, 8:2); mp 86–87 °C. IR (KBr): ν_max_ 3406, 1511, 1466, 1246, 1193, 1179, 1036, 825 cm^−1^. ^1^H-NMR (500 MHz, CDCl_3_): δ = 2.13 (br s, 3H, C*H*_3_Ar), 2.16 (br s, 3H, C*H*_3_Ar), 3.76 (s, 3H, C*H*_3_O), 4.53–4.92 (br s, 2H, OH, NH), 6.61 (br s, 1H, H-6), 6.72–6.76 (m, 2H, H-2′), 6.78–6.81 (m, 2H, H-3′), 6.85 (br s, 1H, H-3). ^13^C-NMR (75 MHz, CDCl_3_): δ = 15.4 (*C*H_3_Ar), 17.4 (*C*H_3_Ar), 55.7 (*C*H_3_O), 114.8 (C-3′), 117.3 (C-6), 118.0 (C-2′), 121.9 (C-2), 123.8 (C-3), 129.0 (C-5), 135.0 (C-1′), 139.4 (C-4), 149.3 (C-1), 153.4 (C-4′). MS (70 eV): *m/z* (%) 243 (M^+^, 91), 228 (100), 200 (11), 185 (9), 168 (7), 134 (5), 77 (7). HRMS (EI): *m/z* [M^+^] calcd for C_15_H_17_NO_2_: 243.1259; found: 243.1260.

*2,5-Dimethyl-4-((4-tolyl)amino)phenol* (**11c**). The procedure for the preparation of **11a** was followed using a mixture of **10c** (0.200 g, 0.89 mmol) in Et_2_O (10 mL) and a saturated aqueous solution of sodium hydrosulfite (30 mL) to give **11c** (0.193 g, 95%) as a brown solid. R*_f_* 0.45 (hexane/EtOAc, 7:3); mp 104–105 °C. IR (film): ν_max_ 3388, 2922, 1614, 1511, 1461, 1196, 993, 811, 738 cm^−1^. ^1^H-NMR (300 MHz, CDCl_3_): δ = 2.12 (s, 3H, C*H*_3_-C5), 2.16 (s, 3H, C*H*_3_-C2), 2.25 (s, 3H, C*H*_3_Ar), 4.70–5.50 (br, 2H, OH, NH), 6.62–6.67 (m, 2H, H-2′), 6.65 (br s, 1H, H-6), 6.93 (br s, 1H, H-3), 6.96–7.02 (m, 2H, H-3′). ^13^C-NMR (75 MHz, CDCl_3_): δ = 15.5 (*C*H_3_-C2), 17.5 (*C*H_3_-C5), 20.5 (*C*H_3_Ar), 115.3 (C-2′), 117.2 (C-6), 121.8 (C-2), 125.6 (C-3), 128.1 (C-4′), 129.7 (C-3′), 130.6 (C-5), 133.6 (C-4), 143.7 (C-1′), 150.1 (C-1). HRMS (EI): *m/z* [M^+^] calcd for C_15_H_17_NO: 227.1310; found: 227.1314.

*4-(Benzyloxy)-N-(4-methoxyphenyl)-2,5-dimethylaniline* (**12a**). To a mixture of **11b** (0.137 g, 0.56 mmol) in acetone (15 mL) K_2_CO_3_ (0.116 g, 0.84 mmol) and benzyl bromide (0.115 g, 0.67 mmol) were added, and the mixture was stirred at reflux for 3 h. The crude mixture was filtered over Celite, concentrated under vacuum, and purified by column chromatography over silica gel (10 g/g crude, hexane/EtOAc, 8:2) to give **12a** (0.169 g, 90%) as a white powder. R*_f_* 0.44 (hexane/EtOAc, 8:2); mp 55–56 °C. IR (KBr): ν_max_ 3412, 2961, 2916, 1521, 1465, 1382, 1293, 1249, 1197, 1098, 1037, 1014, 825, 744, 698 cm^−1^. ^1^H-NMR (500 MHz, CDCl_3_): δ = 2.16 (s, 3H, C*H*_3_-C5), 2.19 (s, 3H, C*H*_3_-C2), 3.72 (s, 3H, C*H*_3_O), 5.00 (s, 2H, C*H*_2_Ph), 6.72–6.80 (m, 4H, H-2′, H-3′), 6.74 (s, 1H, H-6), 6.89 (s, 1H, H-3), 7.29 (t, *J* = 7.5 Hz, 1H, H-4′′), 7.36 (t, *J* = 7.5 Hz, 2H, H-3′′), 7.43 (d, *J* = 7.5 Hz, 2H, H-2′′). ^13^C-NMR (125 MHz, CDCl_3_): δ = 16.0 (*C*H_3_-C2), 17.8 (*C*H_3_-C5), 55.5 (*C*H_3_O), 70.4 (*C*H_2_Ph), 114.6 (C-6), 114.64 (2C-3′), 118.2 (2C-2′), 123.3 (C-3), 125.3 (C-2), 127.1 (2C-2′′), 127.6 (C-4′′), 127.8 (C-5), 128.4 (2C-3′′), 135.1 (C-1′), 137.7 (C-1′′), 139.1 (C-4), 152.4 (C-1), 153.5 (C-4′). MS (70 eV): *m/z* (%) 333 (M^+^, 16), 243 (87), 228 (100), 212 (61), 197 (80), 179 (28), 135 (36), 108 (53), 91 (48), 77 (21). HRMS (EI): *m/z* [M^+^] calcd for C_22_H_23_NO_2_: 333.1729; found: 333.1715.

*Methyl 2-(2,5-dimethyl-4-(phenylamino)phenoxy)acetate* (**12b**). The procedure for the preparation of **12a** was followed with a mixture of **11a** (0.104 g, 0.49 mmol), K_2_CO_3_ (0.102 g, 0.74 mmol) and methyl bromoacetate (0.083 g, 0.54 mmol) to give **12b** (0.086 g, 62%) as a brown powder. R*_f_* 0.41 (hexane/EtOAc, 8:2); mp 102–103 °C. IR (film): ν_max_ 3387, 2923, 1600, 1497, 1406, 1196, 994, 862, 747, 694 cm^−1^. ^1^H-NMR (300 MHz, CDCl_3_): δ = 2.17 (s, 3H, C*H*_3_-C5′), 2.22 (s, 3H, C*H*_3_-C2′), 3.80 (s, 3H, CO_2_C*H*_3_), 4.63 (s, 2H, C*H*_2_CO_2_Me), 5.19 (br s, 1H, NH), 6.58 (s, 1H, H-6′), 6.69–6.75 (m, 2H, H-2′′), 6.78 (t, *J* = 7.2 Hz, 1H, H-4′′), 7.02 (s, 1H, H-3′), 7.14–7.24 (m, 2H, H-3′′). ^13^C-NMR (75 MHz, CDCl_3_): δ = 15.8 (*C*H_3_-C2′), 17.9 (*C*H_3_-C5′), 52.1 (CH_2_CO_2_*C*H_3_), 66.0 (*C*H_2_CO_2_Me), 114.1 (C-6′), 115.0 (2C-2′′), 118.8 (C-4′′), 125.6 (C-2′), 125.8 (C-3′), 129.2 (2C-3′′), 130.0 (C-5′), 134.0 (C-4′), 145.8 (C-1′′), 152.5 (C-1′), 169.8 (*C*O_2_Me). HRMS (EI): *m/z* [M^+^] calcd for C_17_H_19_NO_3_: 285.1365; found: 285.1377.

*Methyl 2-(4-((4-methoxyphenyl)amino)-2,5-dimethylphenoxy)acetate* (**12c**). The procedure for the preparation of **12a** was followed using a mixture of **11b** (0.081 g, 0.33 mmol), K_2_CO_3_ (0.066 g, 0.48 mmol) and methyl bromoacetate (0.057 g, 0.37 mmol) to give **12c** (0.08 g, 76%) as a brown solid. R*_f_* 0.47 (hexane/EtOAc, 8:2); mp 124–125 °C. IR (film): ν_max_ 3340, 2922, 1755, 1508, 1438, 1233, 1198, 1116, 1034, 822 cm^−1^. ^1^H-NMR (300 MHz, CDCl_3_): δ = 2.17 (s, 3H, C*H*_3_-C5′), 2.20 (s, 3H, C*H*_3_-C2′), 3.76 (s, 3H, C*H*_3_O), 3.80 (s, 3H, CO_2_C*H*_3_), 4.61 (s, 2H, C*H*_2_CO_2_Me), 4.99 (br s, 1H, NH), 6.58 (s, 1H, H-6′), 6.79-6.82 (br s, 4H, H-2′′, H-3′′), 6.88 (s, 1H, H-3′). ^13^C-NMR (75 MHz, CDCl_3_): δ = 15.8 (*C*H_3_-C2′), 17.8 (*C*H_3_-C5′), 52.1 (CH_2_CO_2_*C*H_3_), 55.6 (*C*H_3_O), 66.4 (*C*H_2_CO_2_Me), 114.6 (2C-3′′), 114.7 (C-6′), 118.9 (2C-2′′), 122.3 (C-3′), 125.6 (C-2′), 126.9 (C-5′), 136.3 (C-4′), 138.4 (C-1′′), 151.2 (C-1′), 153.7 (C-4′′), 169.9 (*C*O_2_Me). HRMS (EI): *m/z* [M^+^] calcd for C_18_H_21_NO_4_: 315.1471; found: 315.1464.

*4-Methoxy-N-(4-methoxyphenyl)-N,2,5-trimethylaniline* (**12d**). A mixture of **11b** (0.180 g, 0.74 mmol), iodomethane (0.210 g, 1.48 mmol) and K_2_CO_3_ (0.204 g, 1.48 mmol) in acetone (2 mL) was stirred at 60 °C for 24 h. Then, the crude mixture was filtered over Celite and concentrated under vacuum, and the residue was purified by column chromatography over silica gel (10 g/g crude, hexane/EtOAc, 99:1) to give **12d** (0.082 g, 41%) as a pink oil. R*_f_* 0.59 (hexane/EtOAc, 8:2). IR (film): ν_max_ 2929, 1508, 1465, 1240, 1153, 1065, 1038, 818 cm^−1^. ^1^H-NMR (500 MHz, CDCl_3_): δ = 2.11 (s, 3H, C*H*_3_-C2), 2.15 (s, 3H, C*H*_3_-C5), 3.13 (s, 3H, NC*H*_3_), 3.72 (s, 3H, C*H*_3_O-C4′), 3.82 (s, 3H, C*H*_3_O-C4), 6.45–6.51 (m, 2H, H-2′), 6.70 (s, 1H, H-3), 6.74–6.79 (m, 2H, H-3′), 6.87 (s, 1H, H-6). ^13^C-NMR (125 MHz, CDCl_3_): δ = 15.7 (*C*H_3_-C5), 17.8 (*C*H_3_-C2), 39.5 (N*C*H_3_), 55.4 (*C*H_3_O-C4), 55.7 (*C*H_3_O-C4′), 112.3 (C-3), 113.8 (C-2′), 114.6 (C-3′), 125.4 (C-5), 130.0 (C-6), 134.7 (C-2), 139.8 (C-1), 144.4 (C-1′), 151.3 (C-4′), 155.6 (C-4). HRMS (EI): *m/z* [M^+^] calcd for C_17_H_21_NO_2_: 271.1572; found: 271.1573.

*2-(Benzylthio)-4-((4-methoxyphenyl)amino)-3,6-dimethylphenol* (**14a**). A mixture of **10b** (0.096 g, 0.40 mmol), benzylthiol (**13a**) (0.055 g, 0.044 mmol) and DMAP (0.054 g, 0.44 mmol) in CH_2_Cl_2_ (20 mL) was stirred at room temperature for 24 h. Then the mixture was concentrated under vacuum and purified by column chromatography over silica gel (10 g/g crude, hexane/EtOAc, 95:5) to give **14a** (0.080 g, 55%) as a purple oil. R*_f_* 0.75 (hexane/EtOAc, 7:3). IR (film): ν_max_ 3377, 2924, 1507, 1455, 1409, 1232, 1178, 1033, 1007, 820, 697 cm^−1^. ^1^H-NMR (500 MHz, CDCl_3_): δ = 2.15 (s, 3H, C*H*_3_-C3), 2.17 (s, 3H, C*H*_3_-C6), 3.76 (s, 3H, C*H*_3_O), 3.77 (s, 2H, C*H*_2_Ph), 4.91 (br s, 1H, NH), 6.60–6.64 (m, 2H, H-2′′), 6.76–6.80 (m, 2H, H-3′′), 6.88 (s, 1H, OH), 6.92 (s, 1H, H-5), 7.06–7.10 (m, 2H, H-2′), 7.20–7.26 (m, 3H, H-3′, H-4′). ^13^C-NMR (125 MHz, CDCl_3_): δ = 15.9 (*C*H_3_-C3), 16.3 (*C*H_3_-C6), 40.0 (*C*H_2_Ph), 55.7 (*C*H_3_O), 114.8 (C-3′′), 117.2 (C-2′′), 118.2 (C-2), 121.7 (C-6), 127.1 (C-5), 127.4 (C-4′), 128.5 (C-3′), 128.8 (C-2′), 134.0 (C-4), 134.1 (C-3), 137.3 (C-1′), 139.8 (C-1′′), 152.0 (C-1), 153.3 (C-4′′). HRMS (EI): *m/z* [M^+^] calcd for C_22_H_23_NO_2_S: 365.1449; found: 365.1448.

*2-((2-Bromophenyl)thio)-4-((4-methoxyphenyl)amino)-3,6-dimethylphenol* (**14b**). The procedure for the preparation of **14a** was followed using a mixture of **10b** (0.048 g, 0.20 mmol), *o*-bromobenzenethiol (**13b**) (0.038 g, 0.20 mmol) and DMAP (0.025 g, 0.20 mmol) to give **14b** (0.064 g, 75%) as a purple oil. R*_f_* 0.69 (hexane/EtOAc, 7:3). IR (film): ν_max_ 3402, 2924, 1507, 1461, 1444, 1232, 1176, 1035, 1016, 820, 744 cm^−1^. ^1^H-NMR (500 MHz, CDCl_3_): δ = 2.24 (s, 3H, C*H*_3_-C6), 2.26 (s, 3H, C*H*_3_-C3), 3.77 (s, 3H, C*H*_3_O), 5.03 (br s, 1H, NH), 6.51 (d, *J* = 8.0 Hz, 1H, H-6′), 6.56 (s, 1H, HO), 6.70–6.75 (m, 2H, H-2′′), 6.77–6.83 (m, 2H, H-3′′), 6.99 (dd, *J* = 8.0, 7.5 Hz, 1H, H-4′), 7.08 (s, 1H, H-5), 7.10 (dd, *J* = 8.0, 7.5 Hz, 1H, H-5′), 7.53 (d, *J* = 8.0 Hz, 1H, H-3′). ^13^C-NMR (125 MHz, CDCl_3_): δ = 15.9 (*C*H_3_-C3), 16.4 (*C*H_3_-C6), 55.7 (*C*H_3_O), 114.8 (2C-3′′), 115.5 (C-2), 117.9 (2C-2′′), 121.0 (C-2′), 122.8 (C-6), 125.9 (C-6′), 126.7 (C-4′), 127.4 (C-5), 128.0 (C-5′), 133.0 (C-3′), 133.4 (C-4), 135.1 (C-3), 136.6 (C-1′), 139.1 (C-1′′), 152.1 (C-1), 153.7 (C-4′′). HRMS (EI): *m/z* [M^+^] calcd for C_21_H_20_BrNO_2_S: 429.0398; found: 429.0386.

*2-((4-Chlorophenyl)thio)-4-((4-methoxyphenyl)amino)-3,6-dimethylphenol* (**14c**). The procedure for the preparation of **14a** was followed using a mixture of **10b** (0.146 g, 0.61 mmol), *p*-chlorobenzenethiol (**13c**) (0.088 g, 0.61 mmol) and DMAP (0.074 g, 0.61 mmol) to give **14c** (0.232 g, 99%) as a purple oil. R*_f_* 0.58 (hexane/EtOAc, 7:3). IR (film): ν_max_ 3386, 2923, 1508, 1461, 1241, 1181, 1090, 1031, 1009, 816 cm^−1^. ^1^H-NMR (300 MHz, CDCl_3_): δ = 2.23 (s, 3H, C*H*_3_-C6), 2.25 (s, 3H, C*H*_3_-C3), 3.75 (s, 3H, C*H*_3_O), 5.02 (br s, 1H, HN), 5.26 (s, 1H, HO), 6.65–6.74 (m, 2H, H-2′′), 6.75–6.82 (m, 2H, H-3′′), 6.90–6.97 (m, 2H, H-2′), 7.04 (s, 1H, H-5), 7.13–7.22 (m, 2H, H-3′). ^13^C-NMR (75 MHz, CDCl_3_): δ = 15.9 (*C*H_3_-C6), 16.4 (*C*H_3_-C3), 55.6 (*C*H_3_O), 114.7 (C-3′′), 115.4 (Ar), 117.7 (C-2′′), 122.5 (Ar), 127.2 (C-5), 127.3 (2C-2′), 129.2 (2C-3′), 131.6 (Ar), 133.2 (Ar), 134.0 (Ar), 134.9 (Ar), 139.0 (C-1′′), 151.8 (C-1), 153.5 (C-4′′). HRMS (EI): *m/z* [M^+^] calcd for C_21_H_20_ClNO_2_S: 385.0903; found: 385.0900.

*4-((4-Methoxyphenyl)amino)-3,6-dimethyl-3-((4-tolyl)thio)phenol* (**14d**)*.* The procedure for the preparation of **14a** was followed using a mixture of **10b** (0.200 g, 0.83 mmol), *p*-tolylthiol (**13d)** (0.103 g, 0.84 mmol) and DMAP (0.101 g, 0.83 mmol) to give **14d** (0.274 g, 90%) as a purple oil. R*_f_* 0.73 (hexane/EtOAc, 7:3). IR (film): ν_max_ 3394, 2919, 1508, 1473, 1233, 1179, 1090, 1031, 1010, 819 cm^−1^. ^1^H-NMR (300 MHz, CDCl_3_): δ = 2.23 (s, 3H, C*H*_3_-C6), 2.26 (s, 3H, C*H*_3_Ar), 2.27 (s, 3H, C*H*_3_-C3), 3.74 (s, 3H, C*H*_3_O), 4.99 (br s, 1H, HN), 6.66–6.71 (m, 2H, H-2′′), 6.75–6.80 (m, 2H, H-3′′), 6.92–6.96 (m, 2H, H-3′), 7.01 (s, 1H, H-5), 7.00–7.04 (m, 2H, H-2′). ^13^C-NMR (75 MHz, CDCl_3_): δ = 15.9 (*C*H_3_-C3), 16.4 (*C*H_3_-C6), 20.8 (*C*H_3_Ar), 55.6 (*C*H_3_O), 114.7 (C-3′′), 116.6 (C-2), 117.5 (C-2′′), 122.2 (C-6), 126.5 (C-3′), 127.2 (C-5), 129.9 (C-2′), 131.8 (C-4′), 133.7 (C-3), 134.6 (C-4), 135.7 (C-1′), 139.5 (C-1′′), 152.1 (C-1), 153.4 (C-4′′). HRMS (EI): *m/z* [M^+^] calcd for C_22_H_23_NO_2_S: 365.1449; found: 365.1440.

*2,4-Bis((4-Methoxyphenyl)amino)-3,6-dimethylphenol* (**15**). A mixture of **7a** (0.500 g, 2.82 mmol), **2b** (0.694 g, 5.64 mmol) and AcOH (0.051 g, 0.85 mmol) in toluene (30 mL) was stirred at room temperature for 24 h. The crude mixture was concentrated under vacuum, and then Et_2_O (30 mL) and a saturated aqueous solution of sodium hydrosulfite (30 mL) were added, followed by stirring at room temperature for 12 h. The crude mixture was washed with CH_2_Cl_2_ (3 × 10 mL) and the organic layer dried (Na_2_SO_4_), concentrated under vacuum and purified by column chromatography over silica gel (10 g/g crude, hexane/EtOAc, 9:1) to give **11b** (0.426 g, 62%) and **15** (0.278 g, 27%) as a purple solid.

Data of **15a**: R*_f_* 0.32 (hexane/EtOAc, 1:1); mp 70–71 °C. IR (KBr): ν_max_ 3344, 2932, 1629, 1510, 1239, 1033, 825 cm^−1^. ^1^H-NMR (500 MHz, CDCl_3_): δ = 1.97 (s, 3H, C*H*_3_-C3), 2.24 (s, 3H, C*H*_3_-C6), 3.73 (s, 3H, C*H*_3_O), 3.75 (s, 3H, C*H*_3_O), 4.79 (br, 1H, NH), 4.95 (br, 1H, OH), 6.35 (br, 1H, NH), 6.54–6.58 (m, 2H, 2ArH), 6.65–6.69 (m, 2H, 2ArH), 6.74–6.79 (m, 4H, 4ArH), 6.88 (s, 1H, H-5). ^13^C-NMR (125 MHz, CDCl_3_): δ = 12.6 (*C*H_3_-C2), 15.8 (*C*H_3_-C5), 55.6 (*C*H_3_O), 55.7 (*C*H_3_O), 114.7 (2ArH), 114.9 (2ArH), 115.3 (2ArH), 117.3 (2ArH), 121.5 (Ar), 123.6 (C-5), 127.0 (Ar), 127.5 (Ar), 134.1 (Ar), 139.8 (Ar), 140.1 (Ar), 148.6 (C-4), 153.3 (ArO), 153.4 (ArO). MS (70 eV): *m/z* (%) 348 (M^+^, 100), 333 (68), 273 (70), 243 (44), 228 (60), 160 (36), 146 (18), 122 (16), 77 (14). HRMS (EI): *m/z* [M^+^] calcd for C_22_H_24_N_2_O_3_: 364.1787; found: 364.1786.

*4-(Benzyloxy)-N,N′-bis(4-methoxyphenyl)-2,5-dimethylbenzene-1,2-diamine* (**16**). A mixture of **7a** (0.618 g, 3.49 mmol), **2b** (1.073 g, 8.72 mmol) and AcOH (0.063 g, 1.05 mmol) in toluene (40 mL) was stirred at room temperature for 24 h, then concentrated under vacuum and suspended in Et_2_O (30 mL). A saturated aqueous solution of sodium hydrosulfite (30 mL) was added and the mixture was stirred at room temperature for 12 h. The crude mixture was washed with CH_2_Cl_2_ (3 × 10 mL) and the organic layer dried (Na_2_SO_4_) and concentrated under vacuum. The crude mixture was suspended in acetone (30 mL) and K_2_CO_3_ (0.723 g, 5.24 mmol), followed by the addition of benzyl bromide (0.718 g, 4.20 mmol) and stirring at reflux for 3 h. The mixture was filtered over Celite and concentrated under vacuum, then purified by column chromatography over silica gel (10 g/g crude, hexane/EtOAc, 8:2) to give **12a** (0.640 g, 55%) and **16** (0.460 g, 29%) as a purple solid.

Data of **16**: R*_f_* 0.26 (hexane/EtOAc, 8:2); mp 60–61 °C. IR (film): ν_max_ 3377, 2930, 1508, 1457, 1235, 1178, 1034, 823 cm^−1^. ^1^H-NMR (300 MHz, CDCl_3_): δ = 1.99 (s, 3H, C*H*_3_-C2), 2.24 (s, 3H, C*H*_3_-C5), 3.77 (s, 3H, C*H*_3_O), 3.80 (s, 3H, C*H*_3_O), 4.61 (s, 2H, C*H*_2_Ph), 4.80–6.00 (br, 2H, NH), 6.59–6.64 (m, 2H, 2ArH), 6.70 (s, 1H, H-6), 6.77–6.80 (m, 2H, 2ArH), 6.82–6.89 (m, 2H, 2ArH), 6.93–6.99 (m, 2H, 2ArH), 7.23–7.34 (m, 5H, PhH). ^13^C-NMR (75 MHz, CDCl_3_): δ = 13.2 (*C*H_3_-C2), 16.3 (*C*H_3_-C5), 55.61 (*C*H_3_O), 55.62 (*C*H_3_O), 75.5 (*C*H_2_Ph), 114.5 (2ArH), 114.7 (C-6), 116.7 (2ArH), 120.6 (C-2), 120.8 (4ArH), 128.1 (PhH), 128.2 (2PhH), 128.5 (2PhH), 129.0 (C-5), 135.5 (C-3), 137.3 (Ar), 137.4 (Ar), 139.7 (Ar), 140.0 (Ar), 145.6 (C-4), 153.2 (ArO), 154.5 (ArO). HRMS (EI): *m/z* [M^+^] calcd for C_29_H_30_N_2_O_3_: 454.2257; found: 454.2221.

*4-(Allyloxy)-N-(4-methoxyphenyl)-2,5-dimethylaniline* (**17a**). A mixture of **11b** (0.100 g, 0.41 mmol), potassium carbonate (0.062 g, 0.45 mmol) and allyl bromide (0.075 g, 0.62 mmol) in acetone (20 mL) was stirred at 60 °C for 24 h. The crude mixture was filtered over Celite, concentrated under vacuum and purified by column chromatography over silica gel (10 g/g crude, hexane/EtOAc, 95:5) to give **17a** (0.102 g, 88%) as a pink solid. R*_f_* 0.76 (hexane/EtOAc, 7:3); mp 89–90 °C. IR (film): ν_max_ 1506, 1480, 1239, 1212, 1098, 1034, 1010, 947, 815 cm^−1^. ^1^H-NMR (300 MHz, CDCl_3_): δ = 2.17 (s, 3H, C*H*_3_-C5), 2.19 (s, 3H, C*H*_3_-C2), 3.77 (s, 3H, C*H*_3_O), 4.50 (dt, *J* = 5.0, 1.5 Hz, 2H, C*H*_2_CH=), 4.97 (br, 1H, NH), 5.27 (dq, *J* = 10.5, 1.5 Hz, 1H, C*H*_2_=), 5.43 (dq, *J* = 17.4, 1.5 Hz, 1H, C*H*_2_=), 6.01–6.15 (m, 1H, C*H*=), 6.68 (br s, 1H, H-3), 6.73–6.83 (m, 4H, Ar-H), 6.89 (br s, 1H, H-6). ^13^C-NMR (75 MHz, CDCl_3_): δ = 15.9 (*C*H_3_-C5), 17.9 (*C*H_3_-C2), 55.6 (*C*H_3_O), 60.3 (*C*H_2_CH=), 114.4 (C-3), 114.6 (C-3′), 116.8 (*C*H_2_=), 118.2 (C-2′), 123.2 (C-6), 125.2 (C-5), 127.7 (C-2), 133.8 (*C*H=), 134.9 (C-1), 139.1 (C-1′), 152.2 (C-4), 153.5 (C-4′). HRMS (EI): *m/z* [M^+^] calcd for C_18_H_21_NO_2_: 283.1572; found: 283.1570.

*4-(Allyloxy)-2,5-dimethyl-N-(p-tolyl)aniline* (**17b**). The procedure for the preparation of **17a** was followed using a mixture of **11c** (0.162 g, 0.71 mmol), potassium carbonate (0.108 g, 0.78 mmol) and allyl bromide (0.129 g, 1.07 mmol) in acetone (20 mL) to give **17b** (0.137 g. 72%) as a pink solid. R*_f_* 0.71 (hexane/EtOAc, 8:2); mp 59–60 °C. IR (film): ν_max_ 3405, 2918, 1612, 1510, 1410, 1391, 1283, 1196, 1093, 1012, 997, 917, 814 cm^−1^. ^1^H-NMR (300 MHz, CDCl_3_): δ = 2.22 (s, 6H, C*H*_3_-C2, C*H*_3_-C5), 2.29 (s, 3H, C*H*_3_Ar), 4.53–4.62 (m, 2H, C*H*_2_CH=), 5.10 (br s, 1H, NH), 5.30 (dm, *J* = 10. 5 Hz, 1H, C*H*_2_=), 5.47 (dm, *J* = 17.3 Hz, 1H, C*H*_2_=), 6.05–6.21 (m, 1H, C*H*=), 6.67–6.75 (m, 2H, H-2′), 6.72 (br s, 1H, H-3), 7.01 (br s, 1H, H-6), 7.02–7.10 (m, 2H, H-3′). ^13^C-NMR (75 MHz, CDCl_3_): δ = 15.9 (*C*H_3_-C2 or *C*H_3_-C5), 17.9 (*C*H_3_-C5 or *C*H_3_-C2), 20.5 (*C*H_3_Ar), 69.2 (*C*H_2_CH=), 114.1 (C-3), 115.4 (C-2′), 116.8 (*C*H_2_=), 125.1 (C-5), 125.2 (C-6), 128.2 (C-2), 129.5 (C-4′), 129.7 (C-3′), 133.6 (*C*H=), 133.8 (C-1), 143.5 (C-1′), 152.9 (C-4). HRMS (EI): *m*/*z* [M^+^] Calcd for C_18_H_21_NO: 267.1623; found: 267.1627.

*4-(Allyloxy)-3-((4-chlorophenyl)thio)-N-(4-methoxyphenyl)-2,5-dimethylaniline* (**17c**). The procedure for the preparation of **17a** was followed using a mixture of **14c** (0.200 g, 0.52 mmol), potassium carbonate (0.079 g, 0.57 mmol) and allyl bromide (0.094 g, 0.78 mmol) in acetone (20 mL) to give **17c** (0.179 g, 80%) as a pink oil; R*_f_* 0.79 (hexane/EtOAc, 7:3). IR (film): ν_max_ 2920, 1709, 1508, 1473, 1233, 1089, 1030, 1010, 816 cm^−1^. ^1^H-NMR (500 MHz, CDCl_3_): δ = 2.21 (s, 3H, C*H*_3_-C2), 2.26 (s, 3H, C*H*_3_-C5), 3.75 (s, 3H, C*H*_3_O), 4.33 (br d, *J* = 5.5 Hz, 2H, C*H*_2_CH=), 5.14 (br s, 1H, NH), 5.15 (br d, *J* = 11.0 Hz, 1H, C*H*_2_=), 5.28 (br d, *J* = 17.5 Hz, 1H, C*H*_2_=), 5.98–6.08 (m, 1H, C*H*=), 6.81–6.82 (m, 2H, H-3′), 6.86–6.88 (m, 2H, H-2′), 6.93–6.97 (m, 2H, H-2′′), 6.96 (br s, 1H, H-5), 7.09–7.13 (m, 2H, H-3′′). ^13^C-NMR (125 MHz, CDCl_3_): δ = 15.5 (*C*H_3_-C5), 16.7 (*C*H_3_-C2), 55.4 (*C*H_3_O), 74.3 (*C*H_2_CH=), 114.6 (2C-3′), 117.1 (*C*H_2_=), 120.8 (2C-2′), 121.5 (C-6), 125.8 (C-3), 127.3 (2C-2′′), 128.7 (2C-3′′), 130.0 (C-5), 130.3 (C-4′′), 130.4 (C-2), 133.9 (*C*H=), 136.9 (C-1′′), 137.1 (C-1′), 139.9 (C-1), 153.3 (C-4), 154.6 (C-4′). HRMS (EI): *m/z* [M^+^] calcd for C_24_H_24_ClNO_2_S: 425.1216; found: 425.1212.

*(E)-2-Allyl-4-((4-methoxyphenyl)imino)-3,6-dimethylcyclohexa-2,5-dienone* (**18a**). A solution of **17a** (0.050 g, 0.18 mmol) in decaline (1.0 mL) was stirred at 220 °C for 8 h. The crude mixture was concentrated by azeotropic distillation with toluene (3 × 20 mL) under vacuum, and purified by column chromatography over silica gel (10 g/g crude, hexane) to give **18a** (0.032 g, 62%) as a red oil. R*_f_* 0.77 (hexane/EtOAc, 7:3). IR (film): ν_max_ 2922, 1627, 1601, 1498, 1464, 1440, 1288, 1243, 1035, 840, 723 cm^−1^. ^1^H-NMR (500 MHz, CDCl_3_): δ = 1.96 (s, 3H, C*H*_3_-C6), 2.29 (s, 3H, C*H*_3_-C3), 3.35 (br d, *J* = 6.0 Hz, 2H, C*H*_2_CH=), 3.85 (s, 3H, C*H*_3_O), 5.03 (br d, *J* = 10.0 Hz, 1H, C*H*_2_=), 5.08 (br d, *J* = 17.0 Hz, 1H, C*H*_2_=), 5.78–5.88 (m, 1H, C*H*=), 6.79–6.84 (m, 2H, H-2′), 6.87 (br s, 1H, H-5), 6.93–6.97 (m, 2H, H-3′). ^13^C-NMR (125 MHz, CDCl_3_): δ = 13.5 (*C*H_3_-C3), 16.3 (*C*H_3_-C6), 30.7 (*C*H_2_CH=), 55.5 (*C*H_3_O), 114.3 (C-3′), 115.6 (*C*H_2_=), 122.3 (C-2′), 125.1 (C-5), 134.4 (*C*H=), 138.1 (C-3), 140.2 (C-6), 143.5 (C-1′), 145.0 (C-2), 157.6 (C-4), 157.7 (C-4′), 187.2 (C-1). HRMS (EI): *m/z* [M^+^] calcd for C_18_H_21_NO_2_: 283.1572; found: 283.1576.

*2-Allyl-3,6-dimethyl-4-(p-tolylamino)phenol* (**19b**). The procedure for the preparation of **18a** was followed using a mixture of **17b** (0.300 g, 1.12 mmol) in decaline (2 mL) to give **19b** (0.168 g. 56%) as a dark red oil. R*_f_* 0.67 (hexane/EtOAc, 8:2). IR (film): ν_max_ 3542, 3384, 2920, 1635, 1614, 1514, 1473, 1285, 1242, 1180, 910, 810 cm^−1^. ^1^H-NMR (300 MHz, CDCl_3_): δ = 2.13 (s, 3H, C*H*_3_-C3), 2.18 (s, 3H, C*H*_3_-C6), 2.25 (s, 3H, C*H*_3_Ar), 3.47 (dq, *J* = 5.7, 1.8 Hz, 2H, C*H*_2_CH=), 4.70 (br s, 1H, NH), 5.00–5.13 (m, 2H, C*H*_2_=), 5.98 (ddt, *J* = 17.1, 10.2, 5.7 Hz, 1H, C*H*=), 6.54–6.60 (m, 2H, H-2′), 6.88 (s, 1H, H-5), 6.95–7.01 (m, 2H, H-3′). ^13^C-NMR (75 MHz, CDCl_3_): δ = 13.8 (*C*H_3_-C3), 15.8 (*C*H_3_-C6), 20.4 (*C*H_3_Ar), 31.4 (*C*H_2_CH=), 114.8 (C-2′), 115.5 (*C*H_2_=), 121.7 (C-6), 124.3 (C-2), 125.0 (C-5), 127.7 (C-4′), 129.7 (C-3′), 130.5 (C-3), 133.5 (C-4), 135.5 (*C*H=), 144.3 (C-1′), 149.2 (C-1). HRMS (EI): *m/z* [M^+^] Calcd for C_18_H_21_NO: 267.1623; found: 267.1626.

*N-Isopropyl-N′-(3-methoxyphenyl)-2,5-dimethylbenzene-1,4-diamine* (**20a**). The procedure for the preparation of **10a** was followed using a mixture of **8a** (0.299 g, 1.37 mmol), *m*-anisidine (**2d**) (0.169 g, 1.37 mmol) and glacial acetic acid (1.419 g, 23.64 mmol) in toluene (40 mL) to give **20a** (0.117 g, 30%) as a brown solid. R*_f_* 0.54 (hexane/EtOAc, 8:2); mp 83–84 °C. IR (film): ν_max_ 3381, 2963, 1614, 1598, 1519, 1500, 1462, 1410, 1219, 1156, 1041, 839, 756, 688 cm^−1^. ^1^H-NMR (300 MHz, CDCl_3_): δ = 1.25 (d, *J* = 6.0 Hz, 6H, (C*H*_3_)_2_CH), 2.06 (s, 3H, C*H*_3_-C5), 2.17 (s, 3H, C*H*_3_-C2), 3.66 (sept, *J* = 6.3 Hz, 1H, (CH_3_)_2_C*H*), 3.73 (s, 3H, C*H*_3_O), 5.14 (br s, 1H, NH), 6.17 (t, *J* = 2.1 Hz, 1H, H-2′), 6.22–6.30 (m, 2H, H-4′, H-6′), 6.49 (s, 1H, H-3), 6.91 (s, 1H, H-6), 7.05 (t, *J* = 8.3 Hz, 1H, H-5′). ^13^C-NMR (75 MHz, CDCl_3_): δ = 17.1 (*C*H_3_-C5), 18.0 (*C*H_3_-C2), 23.3 ((*C*H_3_)_2_CH), 44.3 ((CH_3_)_2_*C*H), 55.0 (*C*H_3_O), 99.6 (C-2′), 102.9 (C-6′), 106.8 (C-4′), 112.6 (C-3), 120.3 (C-5), 128.3 (C-6), 128.8 (C-1), 129.8 (C-5′), 132.8 (C-2), 143.0 (C-4), 149.0 (C-1′), 160.8 (C-3′). HRMS (EI): *m/z* [M^+^] calcd for C_18_H_24_N_2_O: 284.1889; found: 284.1886.

*N-(4-Chlorophenyl)-N′-isopropyl-2,5-dimethylbenzene-1,4-diamine* (**20b**). The procedure for the preparation of **10a** was followed using a mixture of **8a** (0.200 g, 0.92 mmol), *p*-chloroaniline (**2e**) (0.116 g, 0.91 mmol) and glacial acetic acid (0.943 g, 15.74 mmol) in toluene (40 mL) to give **20b** (0.159 g, 60%) as a brown solid. R*_f_* 0.53 (hexane/EtOAc, 9:1); mp 93–94 °C. IR (film): ν_max_ 3401, 2965, 1596, 1519, 1492, 1303, 1250, 1215, 1171, 999, 818 cm^−1^. ^1^H-NMR (300 MHz, CDCl_3_): δ = 1.24 (d, *J* = 6.3 Hz, 6H, (C*H*_3_)_2_CH), 2.06 (s, 3H, C*H*_3_-C5), 2.14 (s, 3H, C*H*_3_-C2), 3.16 (br s, 1H, NH), 3.66 (sept, *J* = 6.3 Hz, 1H, (CH_3_)_2_C*H*), 5.10 (br s, 1H, NH), 6.49 (s, 1H, H-3), 6.48–6.54 (m, 2H, H-2′), 6.85 (s, 1H, H-6), 7.03–7.09 (m, 2H, H-3′). ^13^C-NMR (75 MHz, CDCl_3_): δ = 17.1 (*C*H_3_-C5), 17.9 (*C*H_3_-C2), 23.2 ((*C*H_3_)_2_CH), 44.2 ((CH_3_)_2_*C*H), 112.5 (C-3), 114.8 (2C-2′), 120.4 (C-5), 122.0 (C-4′), 128.3 (C-6), 128.4 (C-5), 128.9 (2C-3′), 132.9 (C-2), 143.2 (C-4), 146.2 (C-1′). HRMS (EI): *m/z* [M]^+^calcd for C_17_H_21_ClN_2_: 288.1393; found: 288.1401.

*(E,E)-2,5-Dimethyl-N,N′-(diphenyl)cyclohexa-2,5-diene-1,4-diimine* (**21a**). The procedure for the preparation of **10a** was followed with a mixture of **8a** (0.100 g, 0.46 mmol), **2a** (0.093 g, 1.00 mmol) and glacial acetic acid (0.524 g, 8.74 mmol) in toluene (20 mL) to give **21a** (0.050 g, 38%) as a red solid. R*_f_* 0.79 (hexane/EtOAc, 8:2); mp 194-195 °C. IR (film): ν_max_ 1594, 1573, 1481, 1384, 1268, 1164, 895, 821, 759, 697 cm^−1^. ^1^H-NMR (300 MHz, CDCl_3_): δ = 2.13 (d, *J* = 1.2 Hz, 6H, C*H*_3_-C2, C*H*_3_-C5), 6.64 (br d, *J* = 1.2 Hz, 2H, H-3, H-6), 6.58–6.74 (m, 4H, H-2′), 7.10–7.17 (m, 2H, H-4′), 7.33–7.42 (m, 4H, H-3′). ^13^C-NMR (75 MHz, CDCl_3_): δ = 17.9 (2*C*H_3_Ar), 120.1 (4C-2′), 122.8 (C-3, C-6), 124.2 (2C-4′), 128.9 (4C-3′), 143.8 (C-2, C-5), 150.7 (2C-1′), 158.9 (C-1, C-4). Anal. calcd for C_20_H_18_N_2_: C, 83.88; H, 6.34; N, 9.78. Found: C, 83.89; H, 6.33; N, 9.75.

*(E,E)*-*N,N′-Bis(4-methoxyphenyl)-2,5-dimethylcyclohexa-2,5-diene-1,4-diimine* (**21b**). The procedure for the preparation of **10a** was followed with a mixture of **8a** (0.100 g, 0.46 mmol), **2b** (0.123 g, 1.00 mmol) and glacial acetic acid (0.524 g, 8.74 mmol) in toluene (20 mL) to give **21b** (0.114 g, 72%) as a red solid. *R_f_* 0.42 (hexane/EtOAc, 8:2); mp 144–145 °C. IR (KBr): ν_max_ 2959, 1599, 1572, 1499, 1463, 1440, 1243, 1165, 1034, 844, 815, 764 cm^−1^. ^1^H-NMR (500 MHz, CDCl_3_): δ = 2.14 (d, *J* = 1.5 Hz, 6H, *C*H_3_-C2, *C*H_3_-C5), 3.84 (s, 6H, 2C*H*_3_O), 6.73 (br d, *J* = 1.5 Hz, 2H, H-3, H-6), 6.82–6.86 (m, 4H, H-2′), 6.92–6.96 (m, 4H, H-3′). ^13^C-NMR (125 MHz, CDCl_3_): δ = 18.1 (2*C*H_3_Ar), 55.5 (2*C*H_3_O), 114.2 (4C-3′), 122.0 (4C-2′), 122.5 (C-3, C-6), 143.5 (C-2, C-5), 144.0 (2C-1′), 157.0 (2C-4′), 159.0 (C-1, C-4). MS (70 eV): *m/z* (%) 346 (M^+^, 5), 284 (45), 194 (55), 150 (100), 123 (98), 108 (98), 77 (41). HRMS (EI): *m/z* [M^+^] calcd for C_22_H_22_N_2_O_2_: 346.1681; found: 346.1682.

*(E,E)-N,N′-Bis(3-Methoxyphenyl)-2,5-dimethylcyclohexa-2,5-diene-1,4-diimine* (**21c**). The procedure for the preparation of **10a** was followed with a mixture of **8a** (0.100 g, 0.46 mmol), **2d** (0.123 g, 1.00 mmol) and glacial acetic acid (0.477 g, 7.94 mmol) in toluene (20 mL) to give **21c** (0.064 g, 40%) as an orange oil; R*_f_* 0.40 (hexane/EtOAc, 8:2). IR (film): ν_max_ 2921, 1579, 1478, 1280, 1145, 1042, 856, 776, 697 cm^−1^. ^1^H-NMR (300 MHz, CDCl_3_): δ = 2.12 (d, *J* = 1.5 Hz, 6H, *C*H_3_-C2, *C*H_3_-C5), 3.82 (s, 6H, C*H*_3_O), 6.36–6.44 (m, 4H, H-2′, H-4′), 6.65 (br d, *J* = 1.3 Hz, 2H, H-3, H-6), 6.70 (ddd, *J* = 8.4, 2.4, 0.9 Hz, 2H, H-6′), 7.27 (t, *J* = 8.4 Hz, 2H, H-5′). ^13^C-NMR (75 MHz, CDCl_3_): δ = 17.9 (2*C*H_3_Ar), 55.3 (2*C*H_3_O), 105.5 (2C-2′), 109.9 (2C-6′), 112.2 (2C-4′), 123.0 (C-3, C-6), 129.7 (2C-5′), 143.7 (C-2, C-5), 152.1 (2C-1′), 159.0 (C-1, C-4), 160.1 (2C-3′). HRMS (EI): *m/z* [M^+^] calcd for C_22_H_22_N_2_O_2_: 346.1681; found: 346.1686.

*N,N′-Bis(4-Methoxyphenyl)-2,5-dimethylbenzene-1,4-diamine* (**22a**). The procedure for the preparation of **11a** was followed using a mixture of **21b** (0.121 g, 0.35 mmol) in Et_2_O (10 mL) to give **22a** (0.113 g, 93%) as a dark brown solid. *R_f_* 0.56 (hexane/EtOAc, 8:2); mp 145–146 °C. IR (KBr): ν_max_ 3407, 1527, 1509, 1464, 1440, 1390, 1285, 1246, 1178, 1117, 1036, 1002, 825 cm^−1^. ^1^H-NMR (500 MHz, CDCl_3_): δ = 2.13 (s, 6H, 2C*H*_3_Ar), 3.78 (s, 6H, 2C*H*_3_O), 4.88 (br s, 2H, 2NH), 6.80-6.88 (m, 8H, 2H-2′, 2H-3′), 6.90 (br s, 2H, H-3, H-6). ^13^C-NMR (125 MHz, CDCl_3_): δ = 17.5 (2*C*H_3_Ar), 55.6 (2*C*H_3_O), 114.7 (4C-3′), 119.2 (4C-2′), 121.5 (C-3, C-6), 127.0 (C-2, C-5), 136.6 (2C-1′), 138.4 (C-1, C-4), 153.9 (2C-4′). MS (70 eV): *m/z* (%) 348 (M^+^, 5), 284 (100), 269 (98), 241 (98), 210 (27), 127 (20), 122 (53). HRMS (EI): *m/z* [M^+^] calcd for C_22_H_24_N_2_O_2_: 348.1838; found: 348.1846.

*2,5-Dimethyl-N,N′-di-p-tolylbenzene-1,4-diamine* (**22b**). A mixture of **8a** (0.100 g, 0.46 mmol), *p*-toluidine (**2c**) (0.107 g, 1.00 mmol) and AcOH (0.477 g, 7.94 mmol) in toluene (20 mL) was stirred at room temperature for 24 h. The crude mixture was concentrated under vacuum and suspended in Et_2_O (30 mL). A saturated aqueous solution of sodium hydrosulfite (30 mL) was added and the mixture stirred at room temperature for 12 h. The crude mixture was washed with CH_2_Cl_2_ (3 × 10 mL), and the organic layer dried (Na_2_SO_4_) and concentrated under vacuum, before purifying by column chromatography over silica gel (10 g/g crude, hexane/EtOAc, 8:2) to give **22b** (0.11 g, 71%) as a brown solid. R*_f_* 0.83 (hexane/EtOAc, 8:2); mp 134-135 °C. IR (film): ν_max_ 3406, 3053, 1613, 1510, 1459, 1265, 1126, 1039, 896, 813, 738, 704 cm^−1^. ^1^H-NMR (300 MHz, CDCl_3_): δ = 2.19 (s, 6H, C*H*_3_-C2, C*H*_3_-C5), 2.32 (s, 6H, 2C*H*_3_Ar), 4.90–5.30 (br, 2H, NH), 6.79–6.85 (m, 4H, 2H-2′), 7.06 (s, 2H, H-3, H-6), 7.05–7.10 (m, 4H, 2H-3′). ^13^C-NMR (75 MHz, CDCl_3_): δ = 17.6 (*C*H_3_-C2, *C*H_3_-C5), 20.5 (2*C*H_3_Ar), 116.7 (4C-2′), 122.8 (C-3, C-6), 128.1 (2C-4′), 129.0 (C-2, C-5), 129.8 (4C-3′), 136.1 (C-1, C-4), 142.6 (2C-1′). Anal. calcd for C_22_H_24_N_2_: C, 83.50; H, 7.64; N, 8.85. Found: C, 83.50; H, 7.68; N, 8.81.

*N*,*N*′-Bis(3-Methoxyphenyl)-2,5-dimethylbenzene-1,4-diamine (**22c**). The procedure for the preparation of **22b** was followed using a mixture of **8a** (0.100 g, 0.46 mmol) and **2d** (0.123 g, 1.00 mmol) to give **22c** (0.083 g, 52%) as a brown solid. R*_f_* 0.66 (hexane/EtOAc, 8:2); mp 97–98 °C. IR (film): ν_max_ 3381, 2956, 2924, 1599, 1497, 1215, 1156, 1042, 841, 763, 689 cm^−1^. ^1^H-NMR (500 MHz, CDCl_3_): δ = 2.17 (s, 6H, 2C*H*_3_Ar), 3.76 (s, 6H, 2C*H*_3_O), 4.70–5.60 (br, 2H, NH), 6.38-6.42 (m, 4H, H-2′, H-4′), 6.42–6.46 (m, 2H, H-6′), 7.09 (s, 2H, H-3, H-6), 7.12 (t, *J* = 9.0 Hz, 2H, H-5′). ^13^C-NMR (125 MHz, CDCl_3_): δ = 17.6 (2*C*H_3_Ar), 55.1 (2*C*H_3_O), 101.8 (2C-2′), 104.5 (2C-4′), 108.7 (2C-6′), 124.5 (C-3, C-6), 129.3 (C-2, C-5), 130.0 (2C-5′), 135.9 (C-1, C-4), 146.8 (2C-1′), 160.8 (2C-3′). HRMS (EI): *m/z* [M^+^] calcd for C_22_H_24_N_2_O_2_: 348.1838; found: 348.1840.

### 3.3. Single-Crystal X-ray Crystallography

*bis*-Iminoquinone **8a** was obtained as yellow crystals (hexane), which were mounted on glass fibers. Crystallographic measurements were performed on an Oxford XCalibur diffractometer with Mo Kα radiation (λ= 0.71073 Å; graphite monochromator) at room temperature [66]. Two standard reflections were monitored periodically, showing no change during data collection. Unit cell parameters were obtained from least-squares refinement. Intensities were corrected for Lorentz and polarization effects. No absorption correction was applied. Anisotropic temperature factors were introduced for all non-hydrogen atoms. Hydrogen atoms were placed in idealized positions and their atomic coordinates refined. Unit weights were used in the refinement. After being solved using SHELX-97 [67], the structure was visualized and plotted with the MERCURY program package [68,69,70]. Data from **8a**: Formula: C_14_H_22_N_2_; molecular weight: 218.34; cryst. syst.: monoclinic; space group: *P 1 21/a 1*; unit cell parameters: *a*, 9.3369(19), *b*, 7.7080(4), *c*, 15.920(3) (Å); α, 90°, β, 142.07 °, γ, 90°; temp. (°K): 292(2); Z: 3; no. of reflections collected: 7685; no. of independent reflections: 2391; no. of reflections observed: 1743; data collection range: 3.36 < 2θ <32.79; *R*: 0.059; GOF: 1.05 (for complete X-ray data, see Supplementary Material Tables S1–S6).

## 4. Conclusions

A series of new 2,5-dimethylbenzoquinone(alkylimines) **7a**–**b**, 2,5-dimethylbenzoquinone(*bis*-alkyldiimines) **8a**–**c**, and *N*,*N*′-dialkyl-2,5-dimethylbenzene-1,4-diamines **9a**–**c** were synthesized via condensation of diacetyl (**1a**) and α-branched primary amines **6a**–**c**. The scope of the method is limited to using this kind of amine. Substitution of the alkylamine of iminoquinone **7a** by anilines **2a**–**b** followed by the aromatization of the iminoquinones **10** yielded a series of diarylamines **11** and **12**. The addition of benzenethiols **13** to compounds **10** led to polysubstituted diarylamines **14a**–**d**. *p*-Aminodiarylamines and *bis*-diarylamines were also available through this methodology by carrying out substitution of both isopropylamino groups in the *bis*-iminoquinone **8a** with one or two anilines. Interestingly, the Claisen rearrangement of the allyloxy analogue **17a** unexpectedly yielded the iminoquinone **18a**. In contrast, the Claisen rearrangement of **17b** led to the expected *ortho*-allyl phenol **19b**. Further studies of reactions with other α-diketones and amines and their potential use in synthesis are currently under investigation.

## Supplementary Materials

Supplementary materials can be accessed at: http://www.mdpi.com/1420-3049/20/11/19716/s1.

## References

[1] Pripis-Nicolau L., de Revel G., Bertrand A., Maujean A. (2000). Formation of flavor components by the reaction of amino acid and carbonyl compounds in mild conditions. J. Agric. Food Chem..

[2] Haahr A.-M., Bredie W.L.P., Stahnke L.H., Jensen B., Refsgaard H.H.F. (2000). Flavor release of aldehydes and diacetyl in oil/water systems. Food Chem..

[3] Yamamoto Y., Kimachi T., Kanaoka Y., Kato S., Bessho K. (1996). Synthesis and DNA binding properties of amide bond-modified analogues related to distamycin. Tetrahedron Lett..

[4] Maurya R., Singh R., Deepak M., Handa S.S., Yadav P.P., Mishra P.K. (2004). Constituents of *Pterocarpus marsupium*: An ayurvedic crude drug. Phytochemistry.

[5] Furusawa T., Kawano M., Fujita M. (2007). The confined cavity of a coordination cage suppresses the photocleavage of α-diketones to give cyclization products through kinetically unfavorable pathways. Angew. Chem. Int. Ed..

[6] Walsh C.J., Mandal B.K. (1999). Improved synthesis of unsymmetrical heteroaromatic 1,2-diketones and the synthesis of carbazole ring substituted tetraaryl cyclopentadienenones. J. Org. Chem..

[7] Xiang A.X., Watson D.A., Ling T., Theodorakis E.A. (1998). Total synthesis of clerocidin via a novel enantioselective homoallenylboration methodology. J. Org. Chem..

[8] El-Ballouli A.O., Zhang Y., Barlow S., Marder S.R., Al-Sayah M.H., Kaafarani B.R. (2012). Fluorescent detection of anions by dibenzophenazine-based sensors. Tetrahedron Lett..

[9] Wärnmark K., Thomas J.A., Heyke O., Lehn J.-M. (1996). Stereoisomerically controlled inorganic architectures: Synthesis of enantio- and diastereo-merically pure ruthenium-palladium molecular rods from enantiopure building blocks. Chem. Commun..

[10] Ita B.I., Offiong O.E. (2001). The study of the inhibitory properties of benzoin, benzil, benzoin-(4-phenylthiosemicarbazone) and benzil-(4-benzylthiosemicarbazone) on the corrosion of mild steel in hydrochloric acid. Mat. Chem. Phys..

[11] McKenna J.M., Halley F., Souness J.E., McLay I.M., Pickett S.D., Collis A.J., Page K., Ahmed I. (2002). An algorithm-directed two-component library synthesized via solid phase methodology yielding potent and orally bioavailable p38 MAP kinase inhibitors. J. Med. Chem..

[12] Slee D.H., Romano S.J., Yu J., Nguyen T.N., John J.K., Raheja N.K., Axe F.U., Jones T.K., Ripka W.C. (2001). Development of potent non-carbohydrate imidazole-based small molecule selectin inhibitors with antiinflammatory activity. J. Med. Chem..

[13] Hui X., Desrivot J., Bories C., Loiseau P.M., Franck X., Hocquemiller R., Figadère B. (2006). Synthesis and antiprotozoal activity of some new synthetic substituted quinoxalines. Bioorg. Med. Chem. Lett..

[14] Corrales T., Catalina F., Peinado C., Allen N.S. (2003). Free radical macrophotoinitiators: An overview on recent advances. J. Photochem. Photobiol. A: Chem..

[15] Krzeszewski M., Thorsted B., Brewer J., Gryko D.T. (2014). Tetraaryl-, pentaaryl-, and hexaaryl-1,4-dihydropyrrolo[3,2-*b*]pyrroles: Synthesis and optical properties. J. Org. Chem..

[16] Kaur N. (2015). Environmentally benign synthesis of five-membered 1,3-*N*,*N*-heterocycles by microwave irradiation. Synth. Commun..

[17] Deng X., Mani N.S. (2006). An efficient route to 4-aryl-5-pyrimidinylimidazoles via sequential functionalization of 2,4-dichloropyrimidine. Org. Lett..

[18] Martínez V., Burgos C., Alvarez-Builla J., Fernández G., Domingo A., García-Nieto R., Gago F., Manzanares I., Cuevas C., Vaquero J.J. (2004). Benzo[*f*]azino[2,1-*a*]phthalazinium cations: Novel DNA intercaling chromophores with antiproliferative activity. J. Med. Chem..

[19] Wolkenberg S.E., Wisnoski D.D., Leister W.H., Wang Y., Zhao A., Lindsley C.W. (2004). Efficient synthesis of imidazoles from aldehydes and 1,2-diketones using microwave irradiation. Org. Lett..

[20] Ramarao C., Nandipati R., Navakoti R., Kottamasu R. (2012). Synthesis and use of chiral substituted benzenes containing 1,2-diols protected as cyclic acetals. Tetrahedron Lett..

[21] Hoyos P., Sinisterra J.-V., Molinari F., Alcántara A.R., María P.D. (2010). Biocatalytic strategies for the asymmetric synthesis of α-hydroxy ketones. Acc. Chem. Res..

[22] Koike T., Murata K., Ikariya T. (2000). Stereoselective synthesis of optically active α-hydroxy ketones and *anti*-1,2-diols via asymmetric transfer hydrogenation of unsymmetrically substituted 1,2-diketones. Org. Lett..

[23] Wright M.W., Welker M.E. (1996). Transition metal mediated *exo* selective Diels-Alder reactions: Preparation of 2-cobalt-substituted 1,3-dienes containing *C*_2_ symmetric 2,3-dibenzobicyclo[2.2.2]octanedione dioxime equatorial ligands and their use in thermal and Lewis acid catalyzed 4+2 cycloadditions. J. Org. Chem..

[24] Zárate-Zárate D., Aguilar R., Hernández-Benitez R.I., Labarrios E.M., Delgado F., Tamariz J. (2015). Synthesis of α-ketols by functionalization of captodative alkenes and divergent preparation of heterocycles and natural products. Tetrahedron.

[25] Mandal A.B., Gómez A., Trujillo G., Méndez F., Jiménez H.A., Rosales M.J., Martínez R., Delgado F., Tamariz J. (1997). One-pot synthesis and highly regio- and stereoselective Diels-Alder cycloadditions of novel *exo*-2-oxazolidinone dienes. J. Org. Chem..

[26] Fuentes A., Martínez-Palou R., Jiménez-Vázquez H.A., Delgado F., Reyes A., Tamariz J. (2005). Diels-Alder reactions of 2-oxazolidinone dienes in polar solvents using catalysis or non-conventional energy sources. Monatsh. Chem..

[27] Bautista R., Bernal P., Herrera R., Santoyo B.M., Lazcano-Seres J.M., Delgado F., Tamariz J. (2011). Synthesis and Diels-Alder cycloadditions of *exo*-imidazolidin-2-one dienes. J. Org. Chem..

[28] Nagarajan A., Zepeda G., Tamariz J. (1996). Highly selective 1,3-dipolar cycloadditions of captodative olefins 1-acetylvinyl carboxylates to diverse dipoles. Tetrahedron Lett..

[29] Herrera R., Mendoza J.A., Morales M.A., Méndez F., Jiménez-Vázquez H.A., Delgado F., Tamariz J. (2007). Selectivity in 1,3-dipolar cycloadditions of β-substituted captodative olefins—An experimental and DFT transition state study. Eur. J. Org. Chem..

[30] Herrera R., Nagarajan A., Morales M.A., Méndez F., Jiménez-Vázquez H.A., Zepeda L.G., Tamariz J. (2001). Regio- and stereoselectivity of captodative olefins in 1,3-dipolar cycloadditions. A DFT/HSAB theory rational for the observed regiochemistry of nitrones. J. Org. Chem..

[31] Villar L., Bullock J.P., Khan M.M., Nagarajan A., Bates R.W., Bott S.G., Zepeda G., Delgado F., Tamariz J. (1996). Highly stereoselective palladium-catalyzed coupling reactions of captodative olefins acetylvinyl arenecarboxylates. J. Organomet. Chem..

[32] Ortega-Jiménez F., Benavides A., Delgado F., Jiménez-Vázquez H.A., Tamariz J. (2010). Synthesis and reactivity of η^4^-diene-Fe(CO)_3_ complexes from *exo*-2-oxazolidinone dienes. A facile generation of stable conjugates enol-enamido species. Organometallics.

[33] Carson J.F. (1953). Reaction of diacetyl and cyclohexylamine. J. Am. Chem. Soc..

[34] Corbett J.F. (1970). Benzoquinone imines. Part VII. The mechanism and kinetics of the reaction of *p*-benzoquinone di-imines with monohydric phenols and the ultraviolet, infrared, and nuclear magnetic resonance spectra of the resulting indoanilines. J. Chem. Soc. B.

[35] Corbett J.F. (1970). Benzoquinone imines. Part VIII. Mechanism and kinetics of the reaction of *p*-benzoquinone monoimines with monohydric phenols. J. Chem. Soc. B.

[36] El Muslemany K.M., Twite A.A., ElSohly A.M., Obermeyer A.C., Mathies R.A., Francis M.B. (2014). Photoactivated bioconjugation between *ortho*-azidophenols and anilines: A facile approach to biomolecular photopatterning. J. Am. Chem. Soc..

[37] ElSohly A.M., Francis M.B. (2015). Development of oxidative coupling strategies for site-selective protein modification. Acc. Chem. Res..

[38] Belfield A.J., Brown G.R., Foubister A.J. (1999). Recent synthetic advances in the nucleophilic amination of benzenes. Tetrahedron.

[39] Ricci A. (2008). Amino Group Chemistry: From Synthesis to the Life Sciences.

[40] Amer B., Juvik O.J., Dupont F., Francis G.W., Fossen T. (2012). Novel aminoalkaloids from European mistletoe (*Viscum album* L.). Phytochem. Lett..

[41] Knölker H.-J., Reddy K.R., Cordell G.A. (2008). Chemistry and Biology of Carbazole Alkaloids. The Alkaloids Chemistry and Biology.

[42] Schmidt A.W., Reddy K.R., Knölker H.-J. (2012). Occurrence, biogenesis, and synthesis of biologically active carbazole alkaloids. Chem. Rev..

[43] Bautista R., Bernal P., Montiel L.E., Delgado F., Tamariz J. (2011). Total synthesis of the natural carbazoles glycozolicine, mukoline, and mukolidine, starting from 4,5-dimethyleneoxazolidin-2-ones. Synthesis.

[44] Bautista R., Jerezano A.V., Tamariz J. (2012). Synthetic approach for constructing the 1-oxygenated carbazole core and its application to the preparation of natural alkaloids. Synthesis.

[45] Bautista R., Montoya P.A., Rebollar A., Burgueño E., Tamariz J. (2013). Palladium-catalyzed synthesis of natural and unnatural 2-, 5-, and 7-oxygenated carbazole alkaloids from *N*-arylcyclohexane enaminones. Molecules.

[46] Xie Y., Liu S., Liu Y., Wen Y., Deng G.-J. (2012). Palladium-catalyzed one-pot diarylamine formation from nitroarenes and cyclohexanones. Org. Lett..

[47] Ricci A. (2000). Modern Amination Methods.

[48] Collet F., Dodd R.H., Dauban P. (2009). Catalytic C-H amination: Recent progress and future directions. Chem. Commun..

[49] Zhu L., Ye Y.-M., Shao L.-X. (2012). Well-defined NHC-Pd(II)-Im(NHC=*N*-heterocyclic carbene; Im=1-methylimidazole) complex catalyzed C-N coupling of primary amines and aryl chlorides. Tetrahedron.

[50] Hartwig J.F. (1997). Palladium-catalyzed amination of aryl halides: Mechanism and rational catalyst design. Synlett.

[51] Hartwig J.F. (1998). Transition metal catalyzed synthesis of arylamines and aryl ethers from aryl halides and triflates: Scope and mechanism. Angew. Chem. Int. Ed..

[52] Wolfe J.P., Wagaw S., Marcoux J.-F., Buchwald S.L. (1998). Rational development of practical catalysts for aromatic carbon-nitrogen bond formation. Acc. Chem. Res..

[53] Hartwig J.F. (2008). Evolution of a fourth generation catalyst for the amination an thioetherification of aryl halides. Acc. Chem. Res..

[54] Surry D.S., Buchwald S.L. (2008). Biaryl phosphine ligands in palladium-catalyzed amination. Angew. Chem. Int. Ed..

[55] Kunz K., Scholz U., Ganzer D. (2003). Renaissance of Ullmann and Goldberg reactions: Progress in copper catalyzed C–N, C–O and C–S coupling. Synlett.

[56] Ley S.V., Thomas A.W. (2003). Modern synthetic methods for copper-mediated C(aryl)-O, C(aryl)-N and C(aryl)-S bond formation. Angew. Chem. Int. Ed..

[57] Fleming I. (2010). Molecular Orbitals and Organic Chemical Reactions, Reference Edition.

[58] Konovalova S.A., Avdeenko A.P., Santalove A.A., D′yakonenko V.V., Palamarchuk G.V., Shishkin O.V. (2014). Reaction of *N*-aryl-1,4-benzoquinone imines with sodium arenesulfinates. Russian J. Org. Chem..

[59] Boone H.W., Bryce J., Lindgren T., Padias A.B., Hall H.K. (1997). Stereoregular poly(benzoquinone imines) from methyl-substituted benzoquinones. Macromolecules.

[60] Jian F.-F., Zhuang R.-R., Wang K.-F., Wang J. (2008). (6*E*)-*N*-[(4*Z*)-2,5-Dimethyl-4-(*p*-tolylimino)cyclohexa-2,5-diphenylidene]-4-methylaniline. Acta Cryst..

[61] Martín Castro A.M. (2004). Claisen rearrangement over the past nine decades. Chem. Rev..

[62] Ganem B. (1996). The mechanism of the Claisen rearrangement: Déjà vu all over again. Angew. Chem. Int. Ed. Engl..

[63] Li X.-G., Huang M.-R., Duan W., Yang Y.-L. (2002). Novel multifunctional polymers from aromatic diamines by oxidative polymerizations. Chem. Rev..

[64] Nishiumi T., Nomura Y., Chimoto Y., Higuchi M., Yamamoto K. (2004). The class II/III transition electron transfer on an infrared vibrational time scale for *N*,*N*′-diphenyl-1,4-phenylenediamine structures. J. Phys. Chem. B.

[65] Nishiumi T., Chimoto Y., Hagiwara Y., Higuchi M., Yamamoto K. (2004). First redox polymer bearing one-step successive two-electron transfer process based on redox potential inversion. Macromolecules.

[B66-molecules-20-19716] 66.CCDC 1429959 contains the supplementary crystallographic data for this paper. These data can be obtained free of charge via http://www.ccdc.cam.ac.uk/conts/retrieving.html (or from the CCDC, 12 Union Road, Cambridge CB2 1EZ, UK; Fax: +44 1223 336033; E-mail: deposit@ccdc.cam.ac.uk).

[67] Sheldrick G.M. (1997). SHELXL-97, Program for Crystal Structure Refinement.

[68] Bruno I.J., Cole J.C., Edgington P.R., Kessler M., Macrae C.F., McCabe P., Pearson J., Taylor R. (2002). New software for searching the Cambridge Structural Database and visualizing crystal structures. Acta Cryst..

[69] Macrae C.F., Edgington P.R., McCabe P., Pidcock E., Shields G.P., Taylor R., Towler M., van de Streek J. (2006). Mercury: Visualization and analysis of crystal structures. J. Appl. Cryst..

[70] Macrae C.F., Bruno I.J., Chisholm J.A., Edgington P.R., McCabe P., Pidcock E., Rodriguez-Monge L., Taylor R., van de Streek J., Wood P.A. (2008). Mercury CSD 2.0—New features for the visualization and investigation of crystal structures. J. Appl. Cryst..

