# Supplementary material for: Condensation of Diacetyl with Alkyl Amines: Synthesis and Reactivity of p-Iminobenzoquinones and p-Diiminobenzoquinones
Source: Molecules. 2015 Nov 20;20(11):20719–40. doi: 10.3390/molecules201119716 (PMC6332260; doi:10.3390/molecules201119716)
Supplement: Supplementary file 1 [file molecules-20-19716-s001.pdf]

Supplementary Materials: Condensation of Diacetyl with Alkyl Amines: Synthesis and Reactivity of *p*-Iminobenzoquinones and *p*-Diiminobenzoquinones

Carlos Espinoza-Hicks, Rafael Bautista, Saúl Frias-Puente, Vanessa Pelayo, Eder I. Martínez-Mora, Francisco Delgado and Joaquín Tamariz

1. ^{1}H-NMR and ^{13}C-NMR of the New Compounds

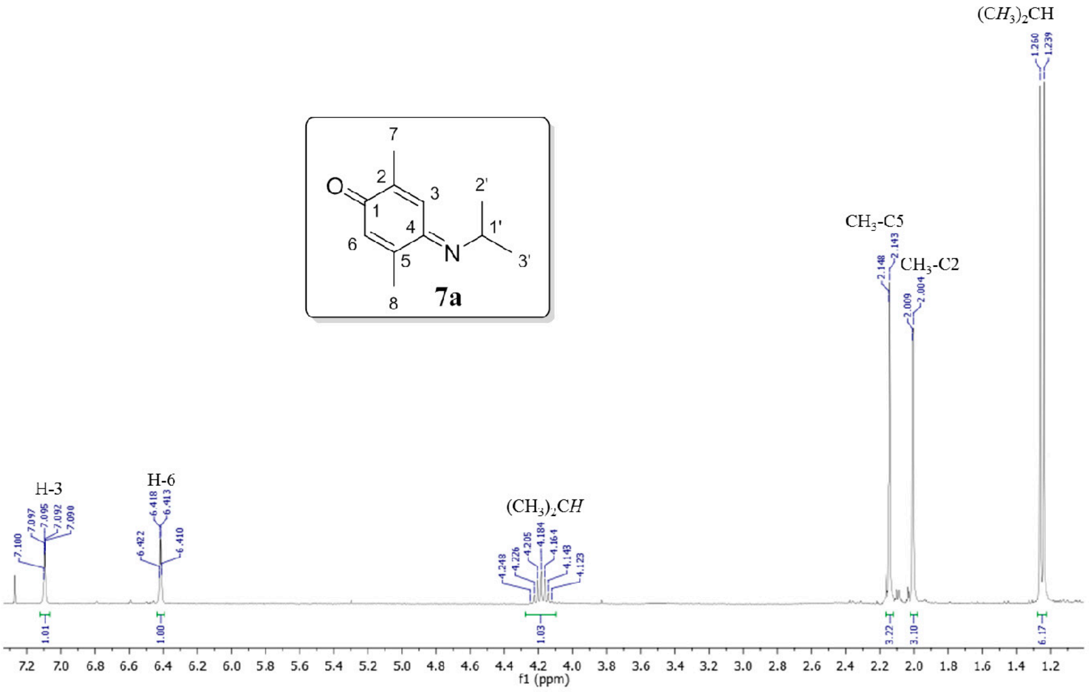

^{1}H-NMR ($CDCl_3$, 500 MHz) spectrum of **7a**.

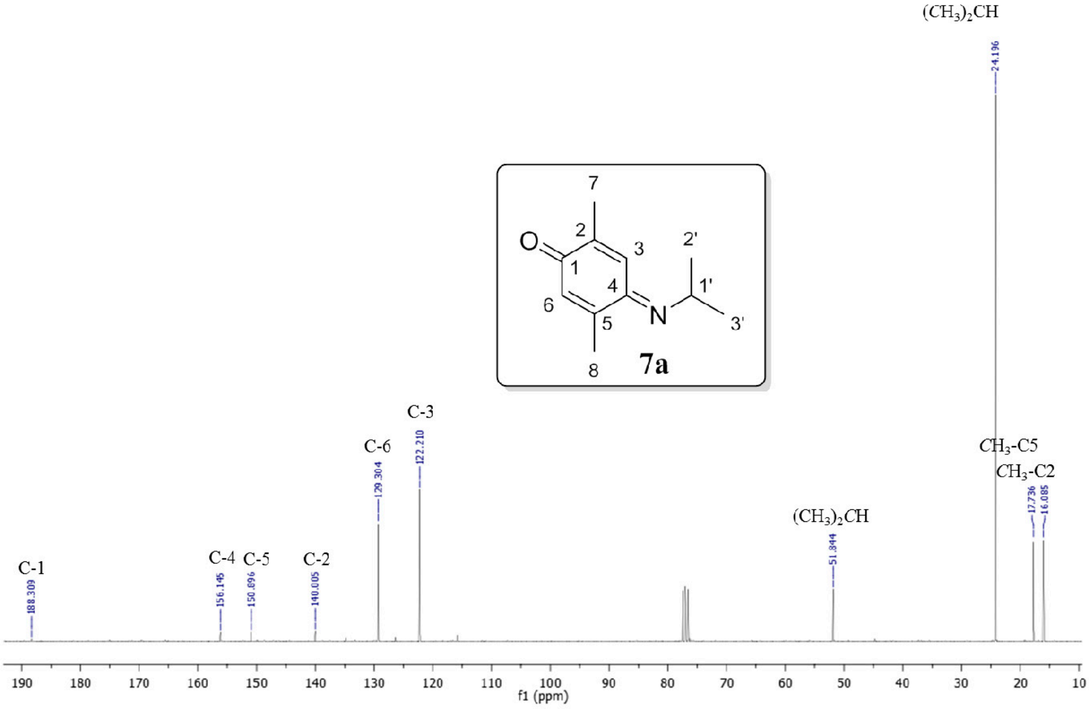

^{13}C-NMR ($CDCl_3$, 125 MHz) spectrum of **7a**.

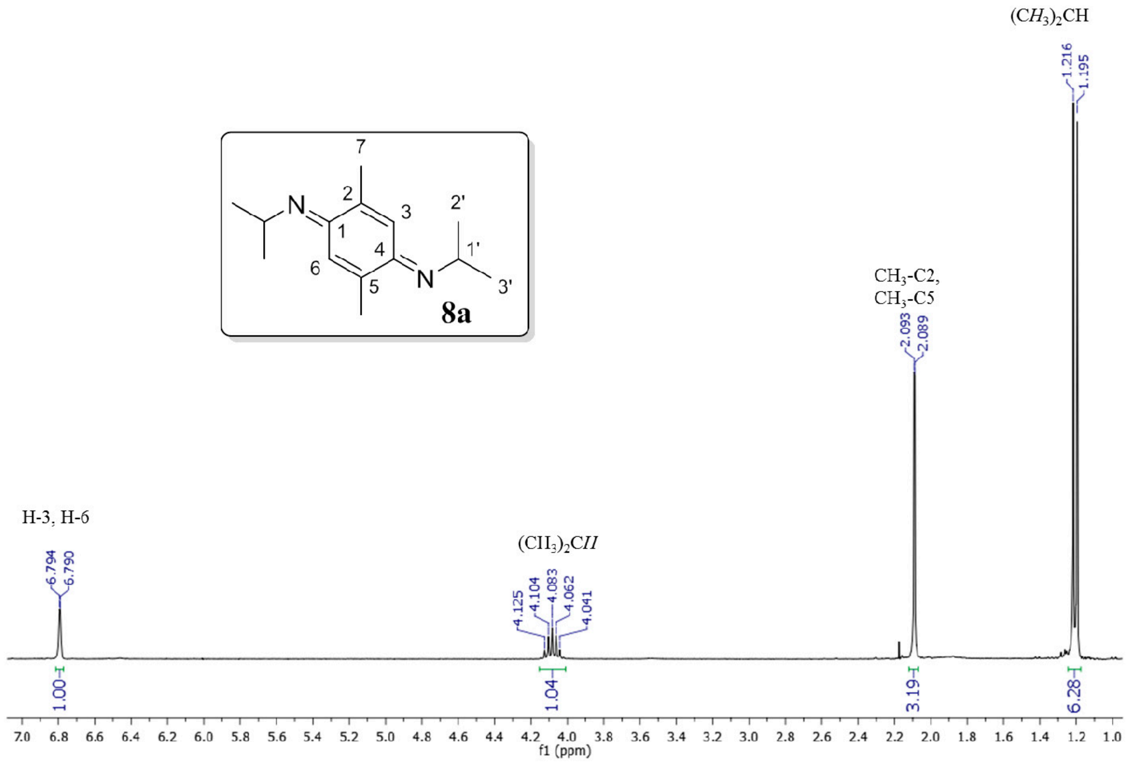

^{1}H-NMR ($CDCl_3$, 500 MHz) spectrum of **8a**.

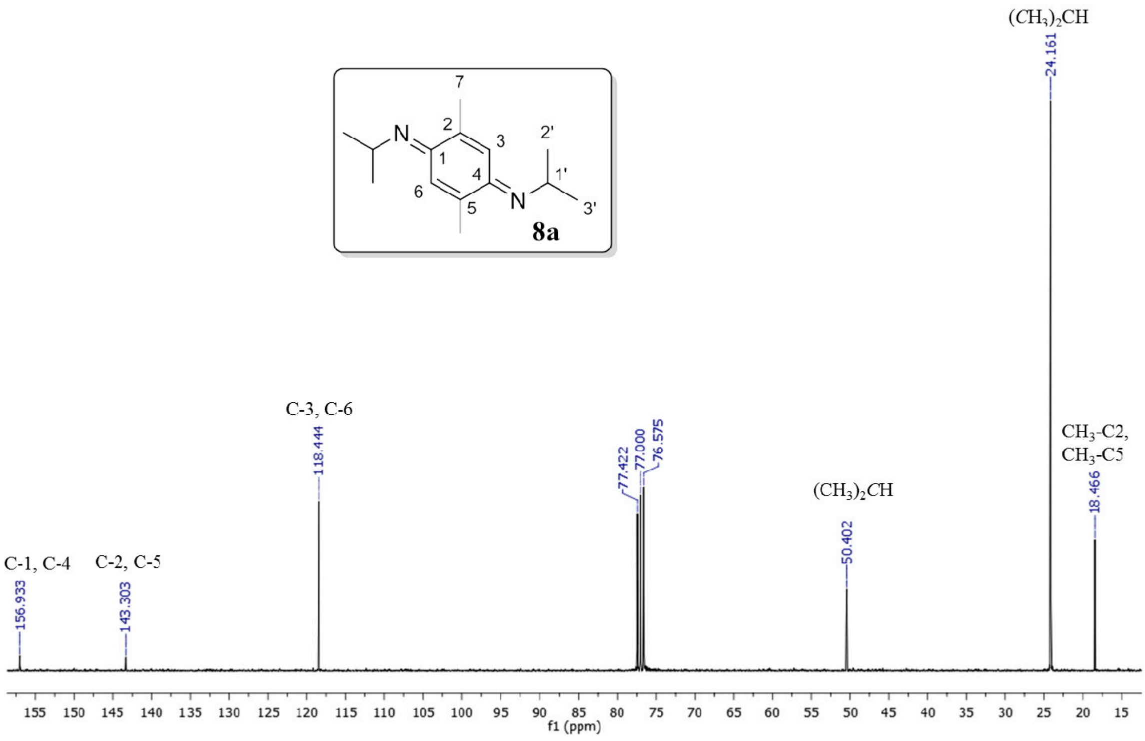

^{13}C-NMR ($CDCl_3$, 125 MHz) spectrum of **8a**.

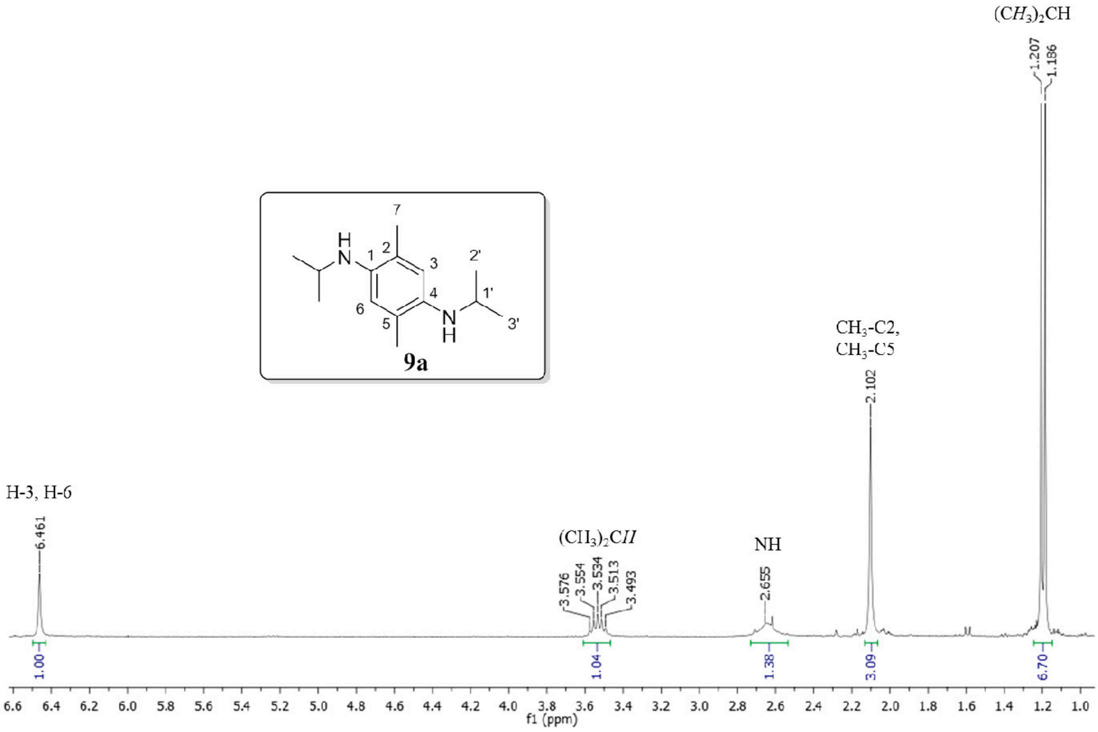

^{1}H-NMR ($CDCl_3$, 500 MHz) spectrum of **9a**.

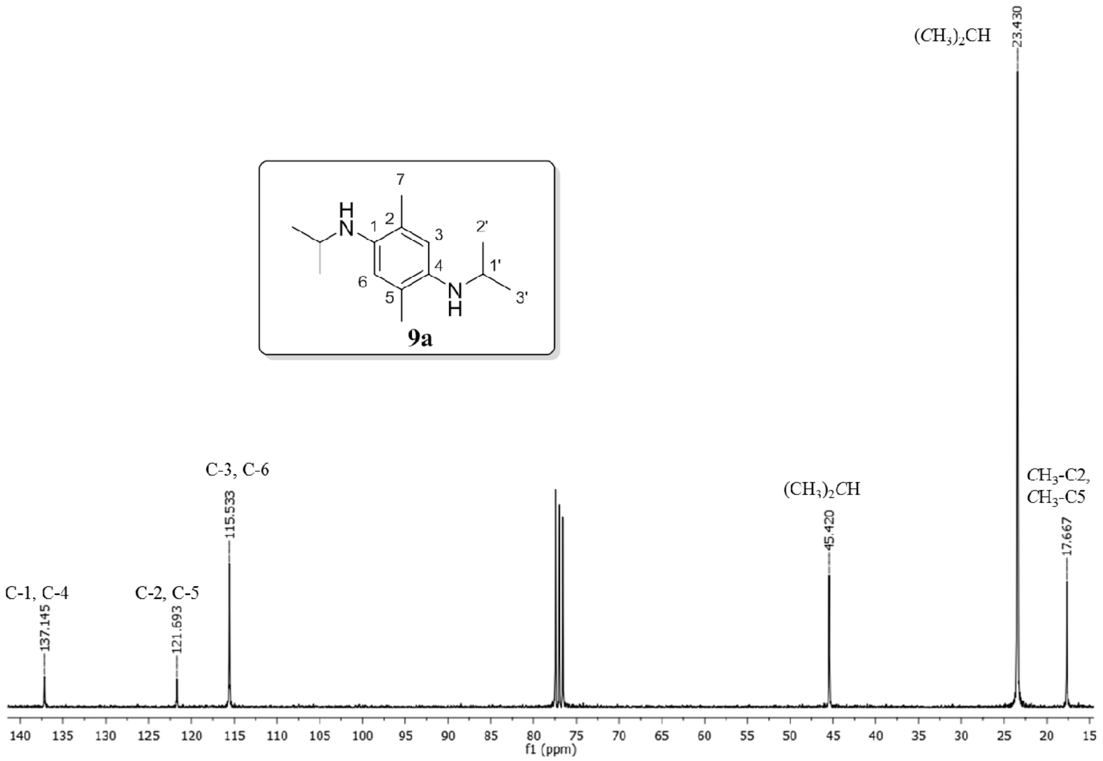

^{13}C-NMR ($CDCl_3$, 125 MHz) spectrum of **9a**.

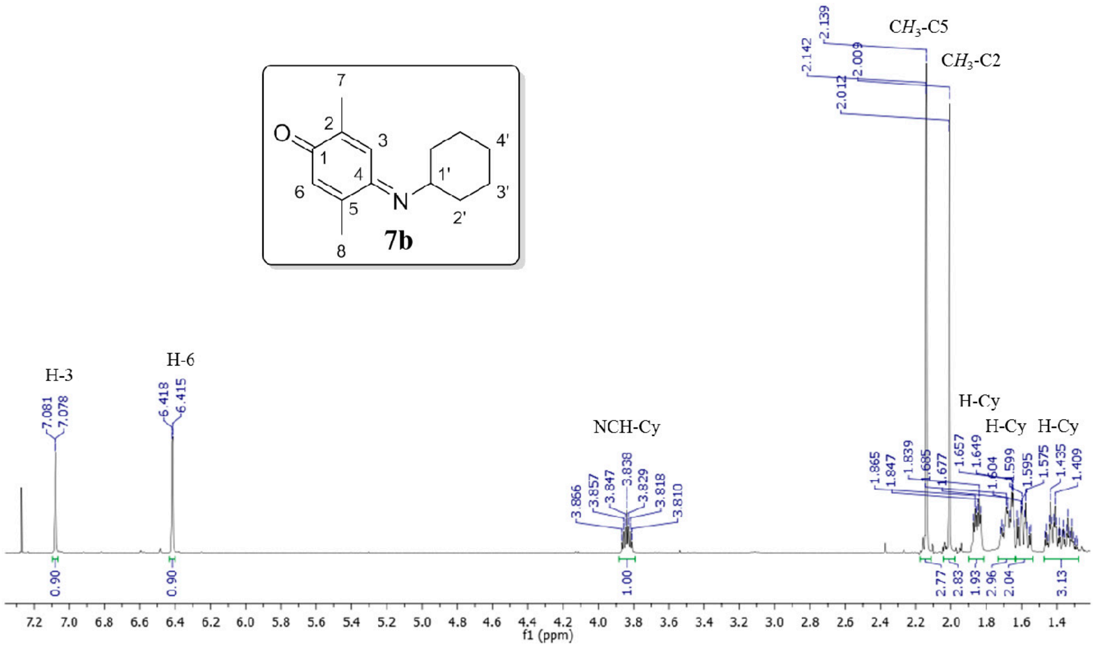

^{1}H-NMR ($CDCl_3$, 500 MHz) spectrum of **7b**.

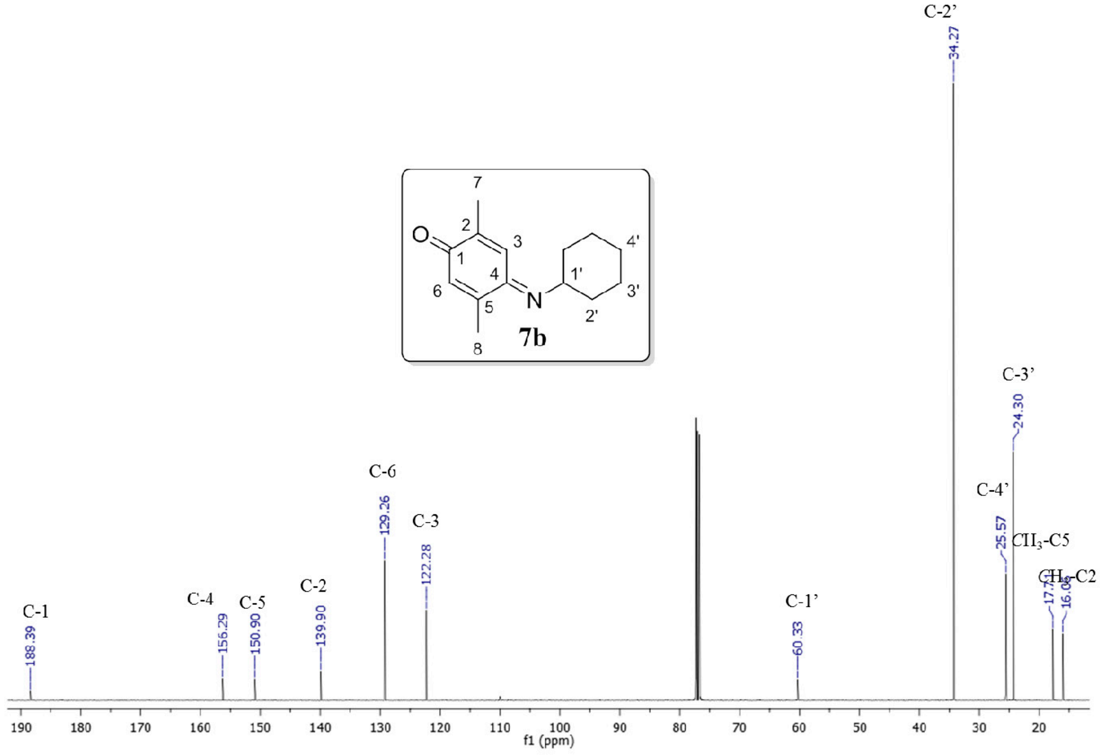

^{13}C-NMR ($CDCl_3$, 125 MHz) spectrum of **7b**.

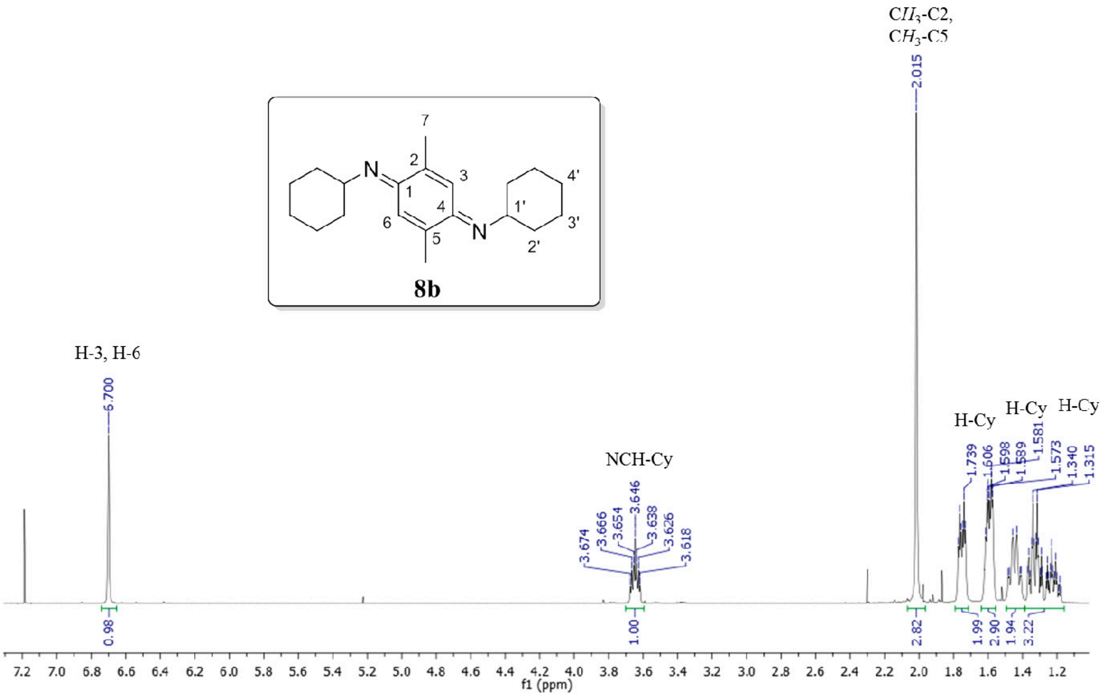

^{1}H-NMR (CDCl$_3$, 500 MHz) spectrum of **8b**.

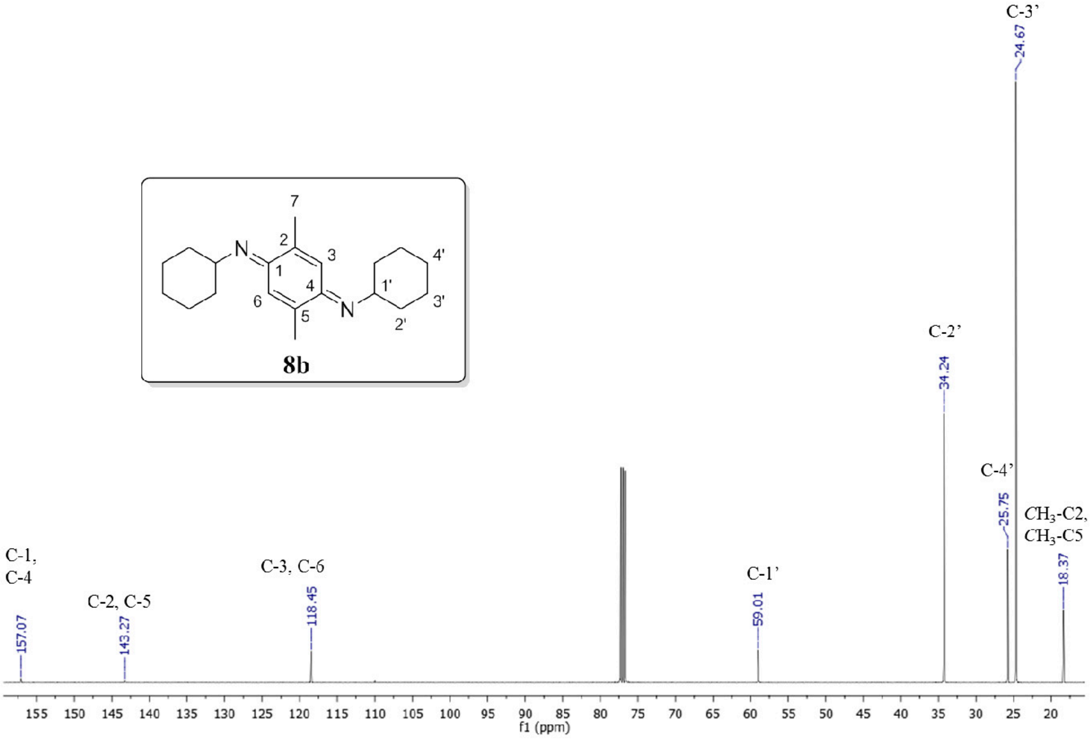

^{1}H-NMR (CDCl$_3$, 125 MHz) spectrum of **8b**.

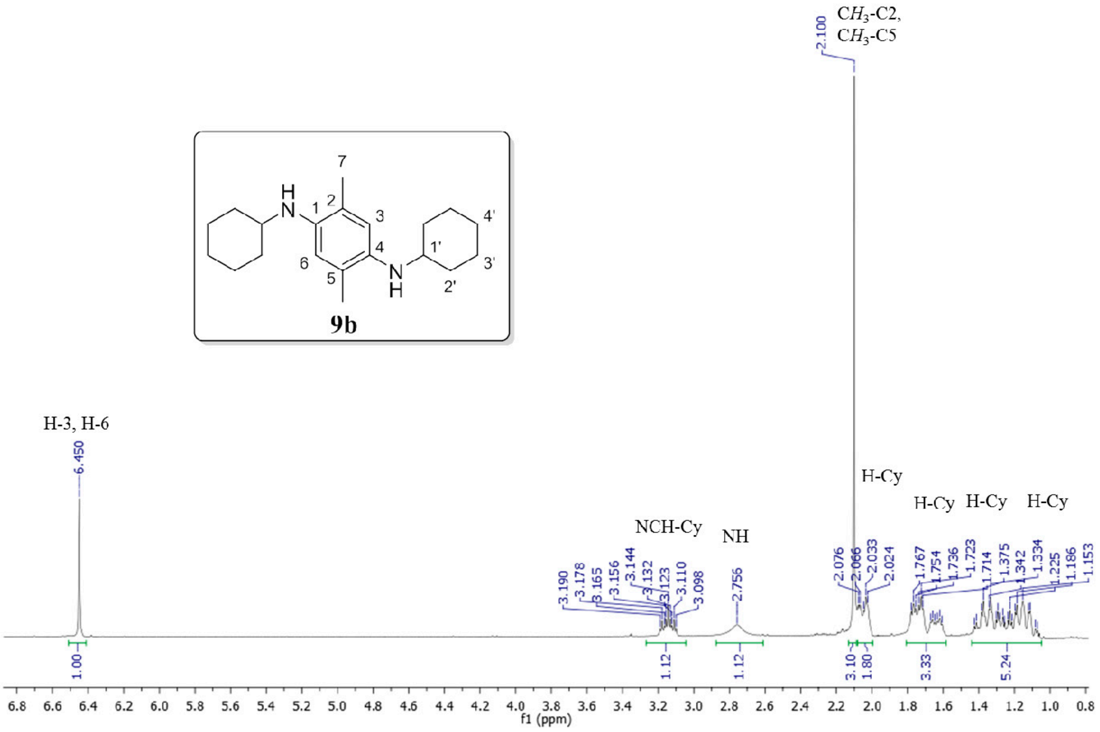

^{1}H-NMR ($CDCl_3$, 300 MHz) spectrum of **9b**.

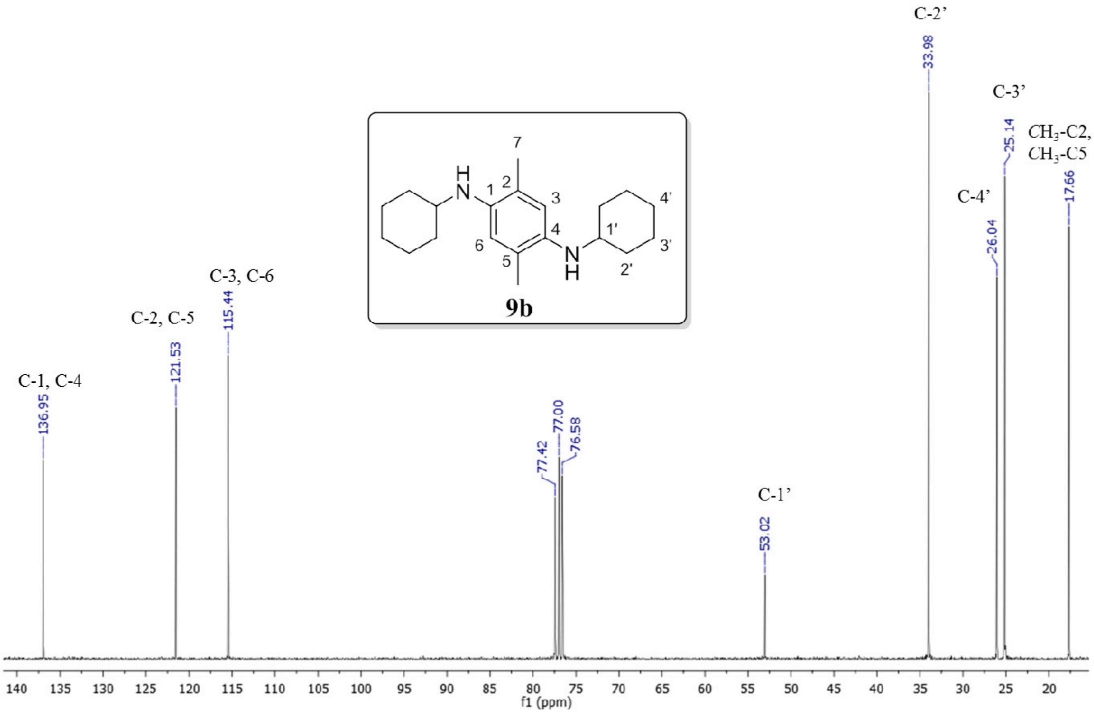

^{13}C-NMR ($CDCl_3$, 75 MHz) spectrum of **9b**.

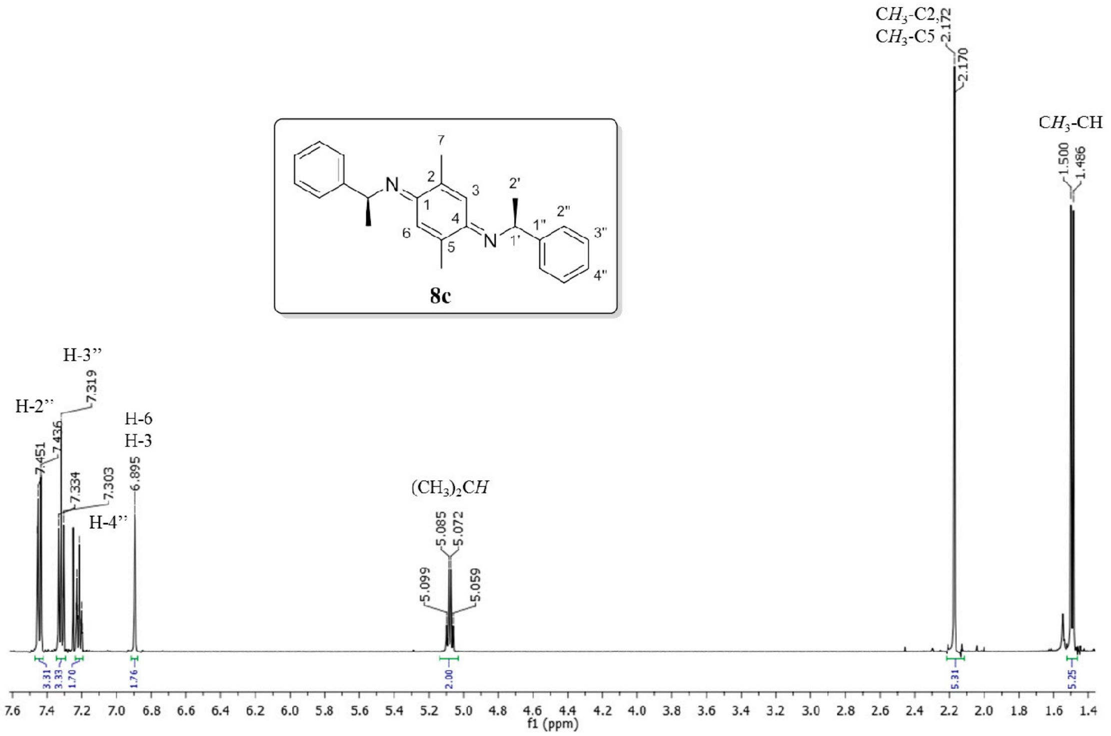

^{1}H-NMR ($CDCl_3$, 500 MHz) spectrum of **8c**.

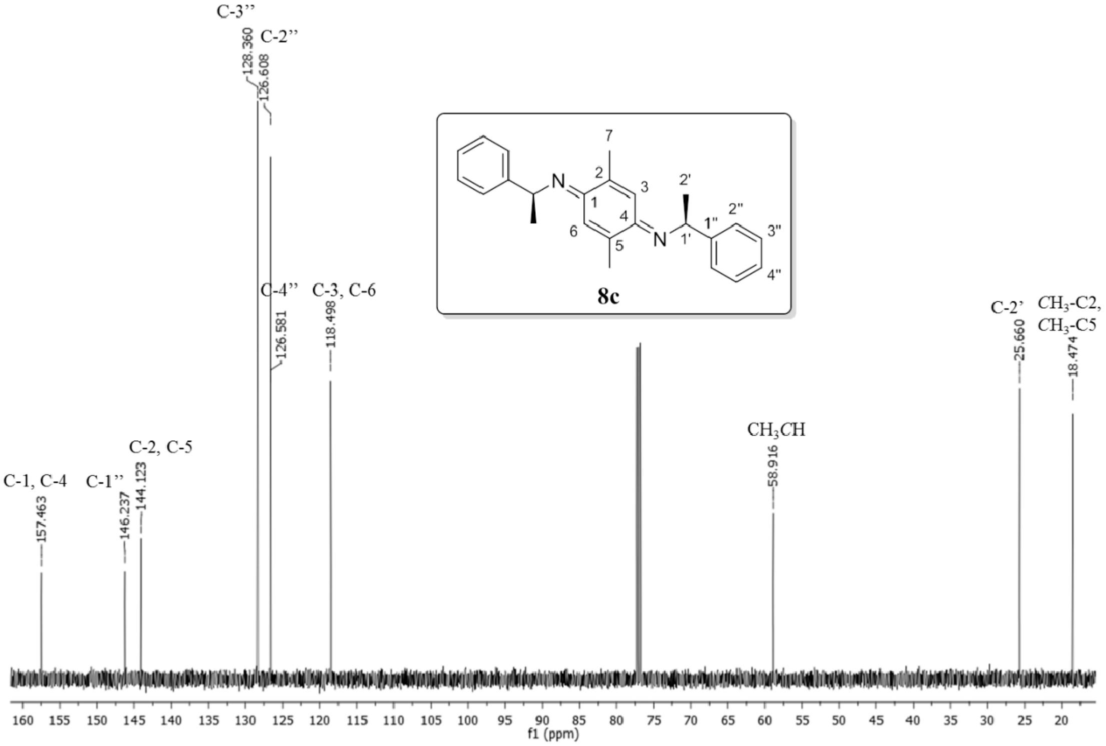

^{13}C-NMR ($CDCl_3$, 125 MHz) spectrum of **8c**.

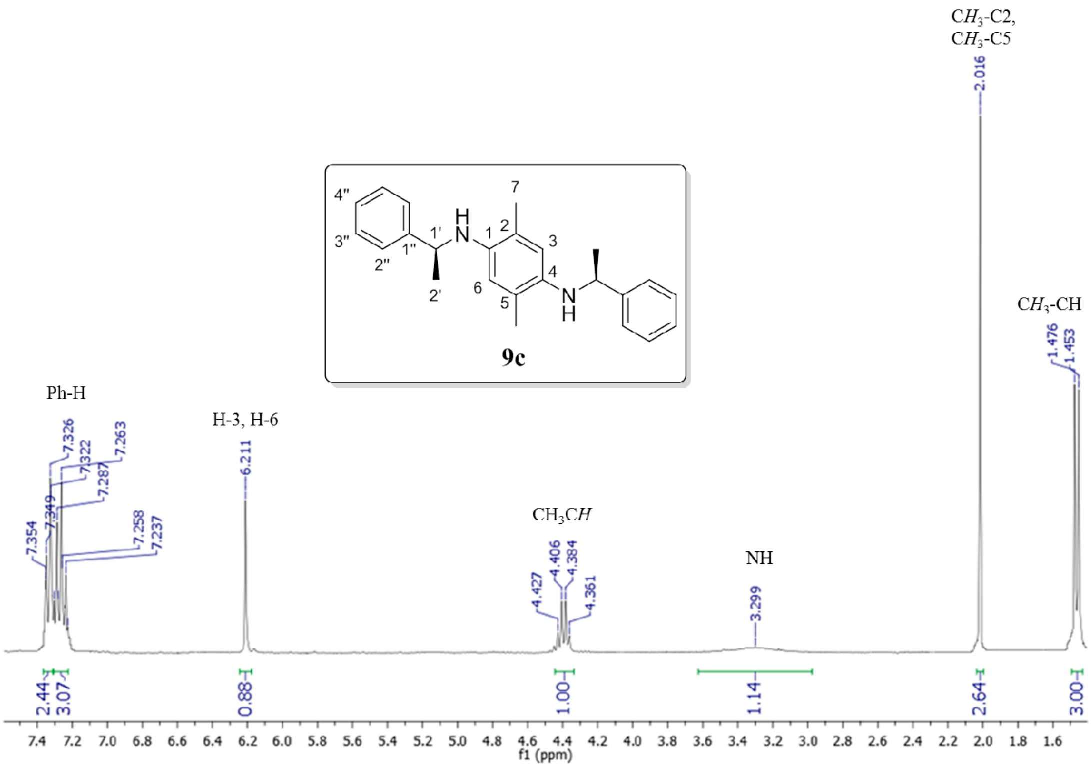

^{1}H-NMR ($CDCl_3$, 300 MHz) spectrum of **9c**.

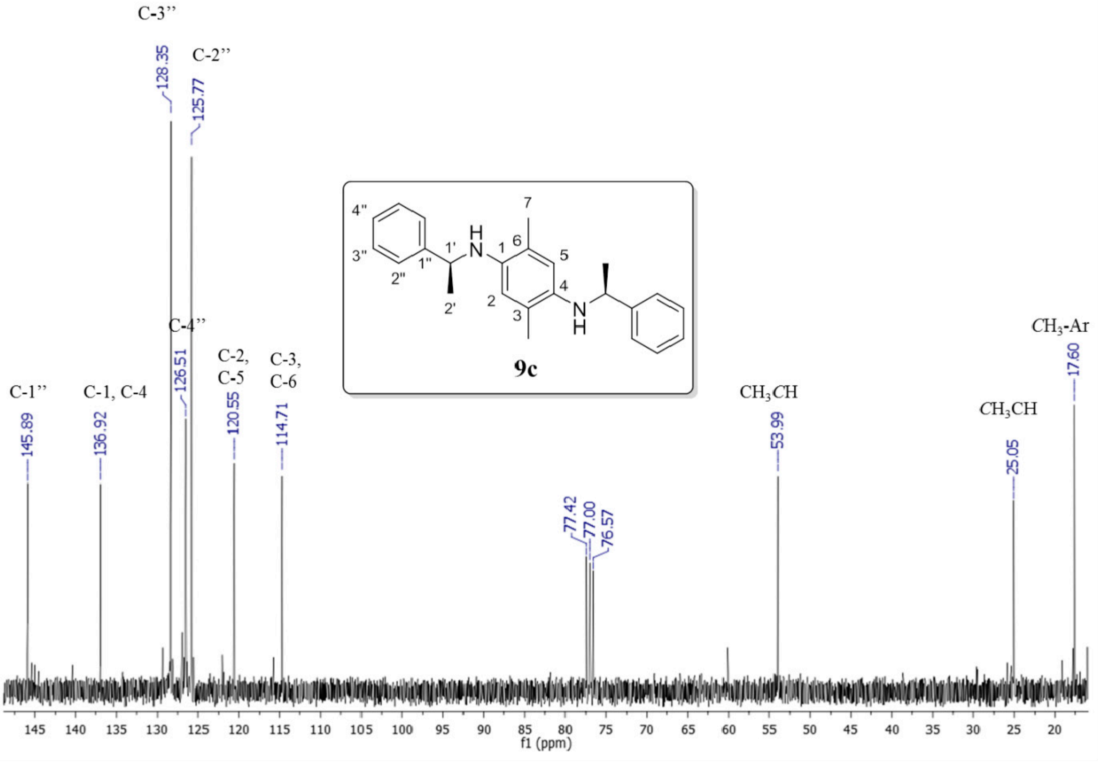

^{13}C-NMR ($CDCl_3$, 75 MHz) spectrum of **9c**.

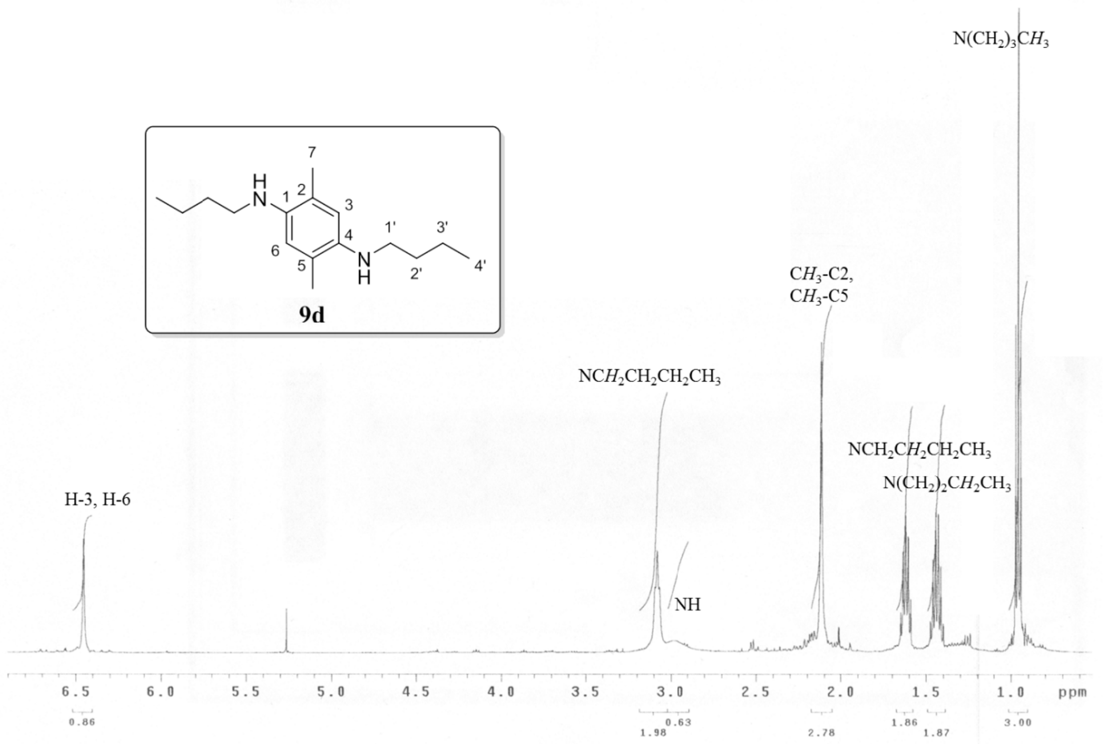

^{1}H-NMR ($CDCl_3$, 500 MHz) spectrum of **9d**.

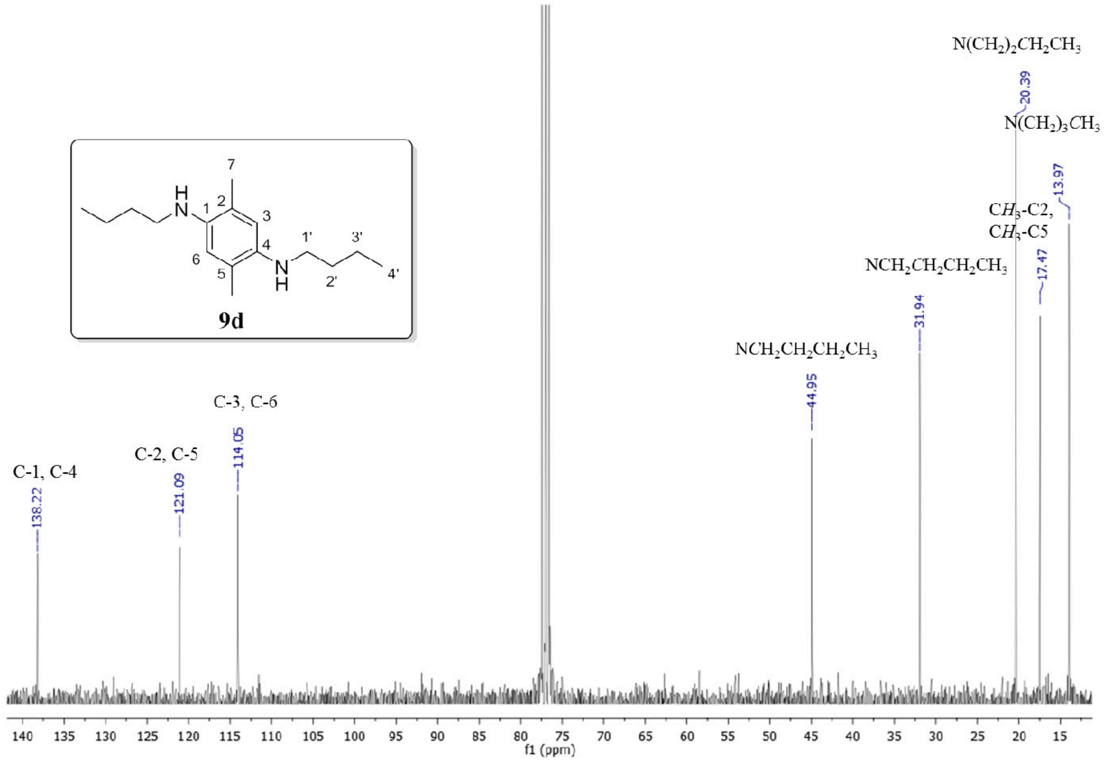

^{13}C-NMR ($CDCl_3$, 125 MHz) spectrum of **9d**.

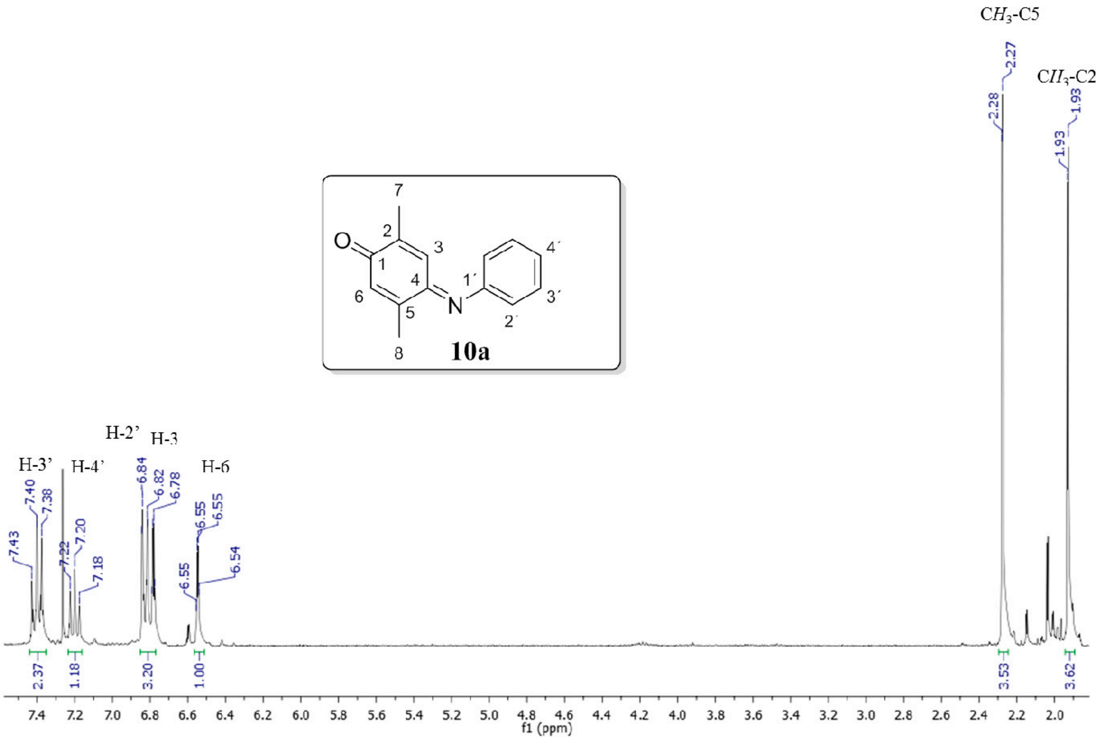

^{1}H-NMR ($CDCl_3$, 300 MHz) spectrum of **10a**

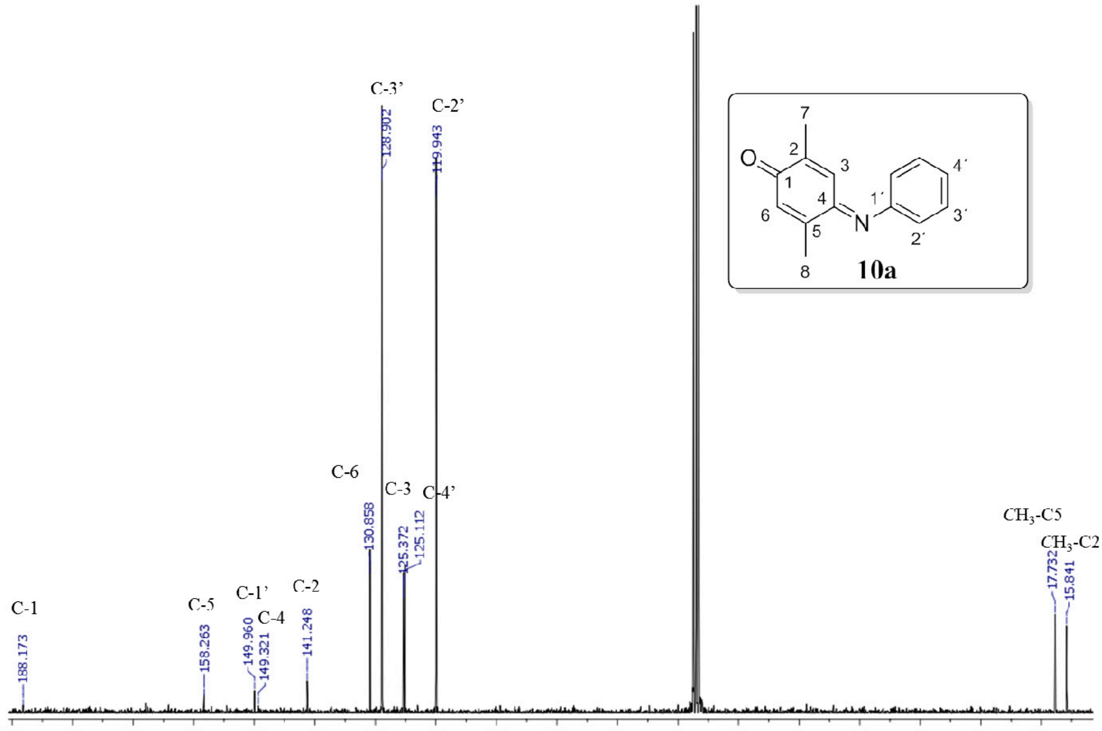

^{13}C-NMR ($CDCl_3$, 75 MHz) spectrum of **10a**

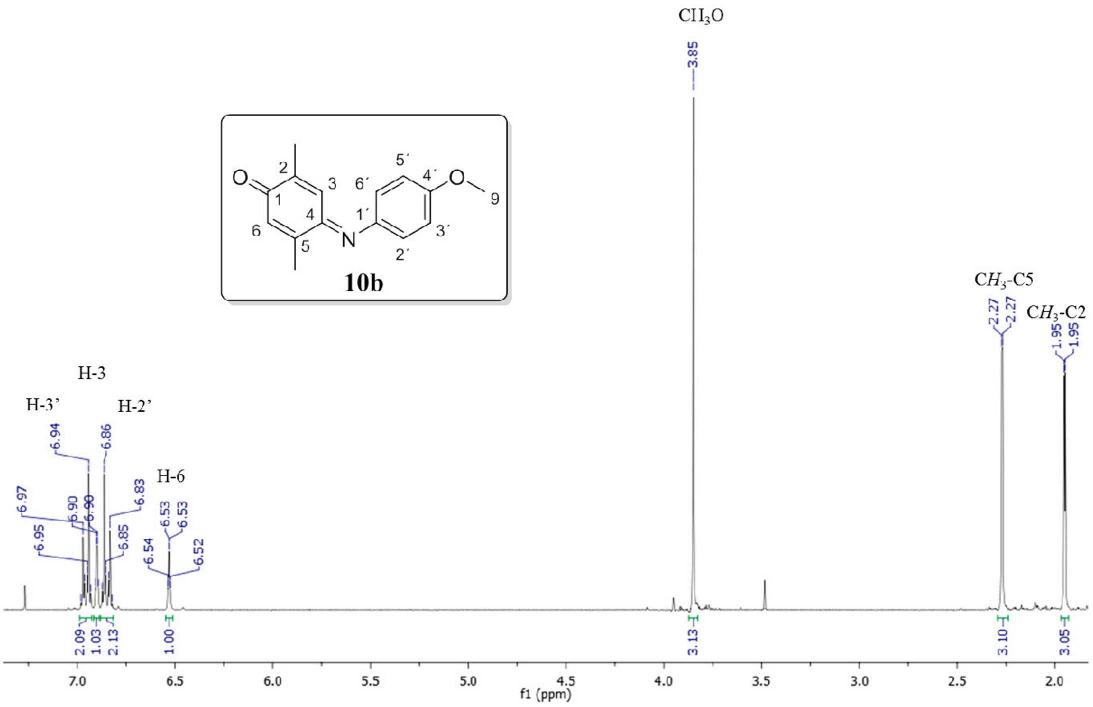

^{1}H-NMR ($CDCl_3$, 500 MHz) spectrum of **10b**.

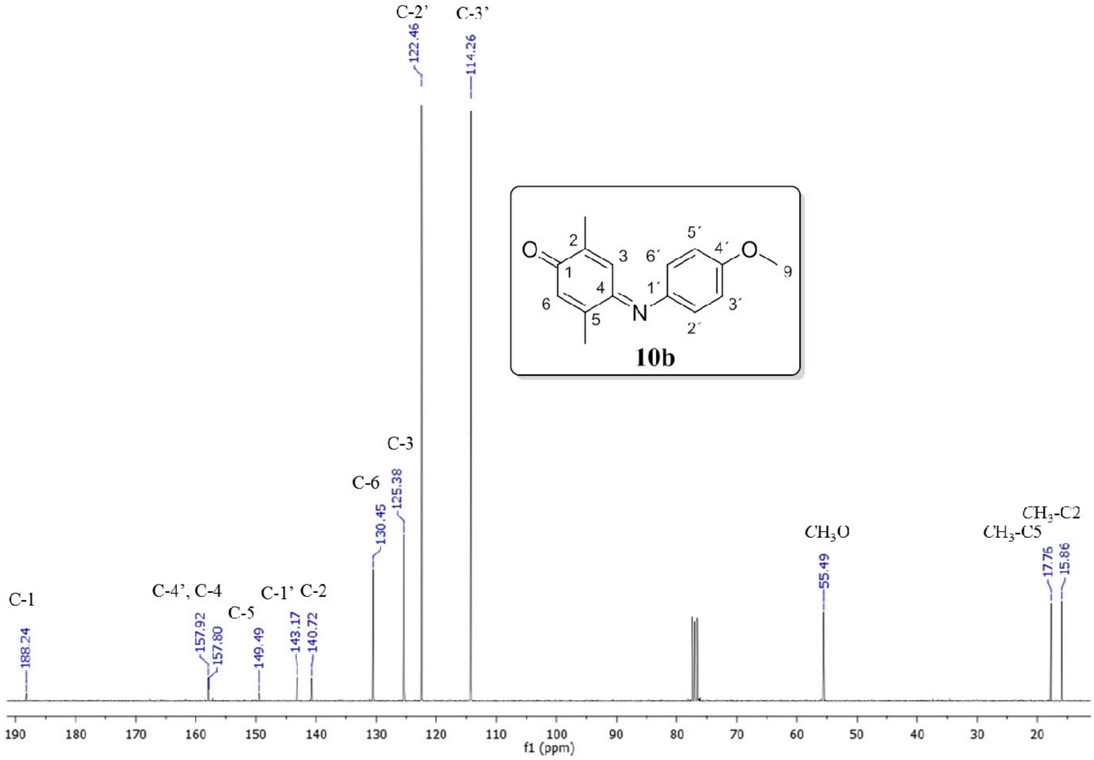

^{13}C-NMR ($CDCl_3$, 125 MHz) spectrum of **10b**.

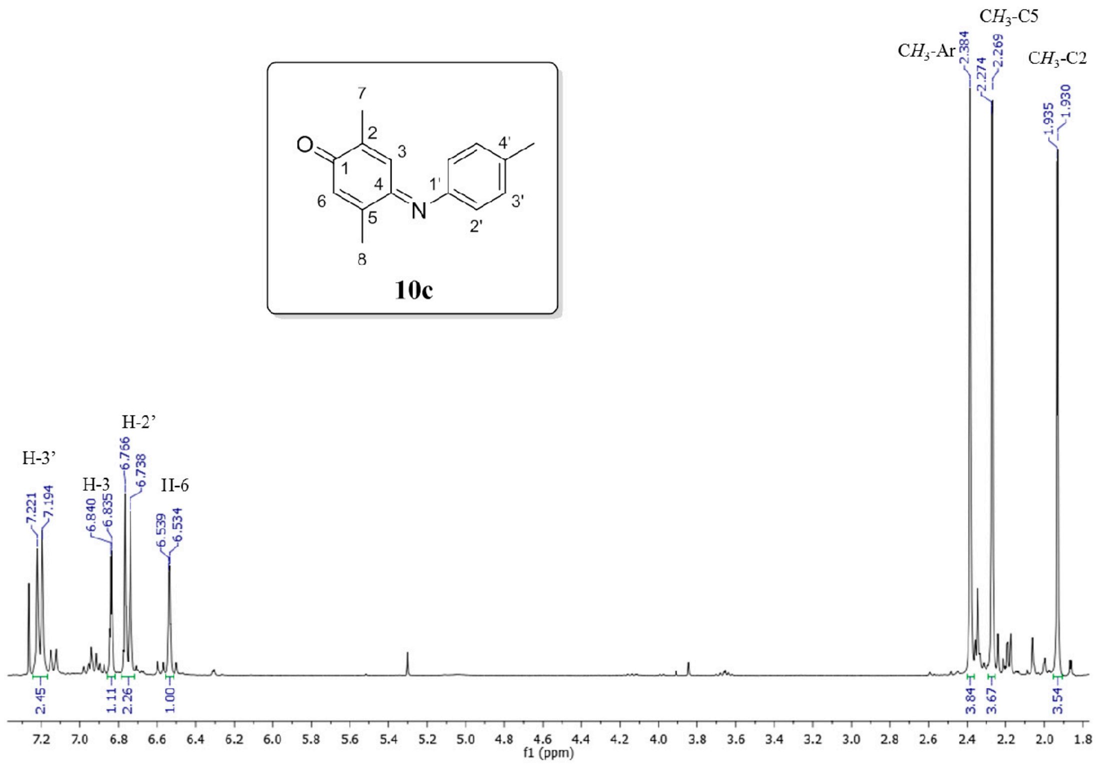

^{1}H-NMR ($CDCl_3$, 300 MHz) spectrum of **10c**.

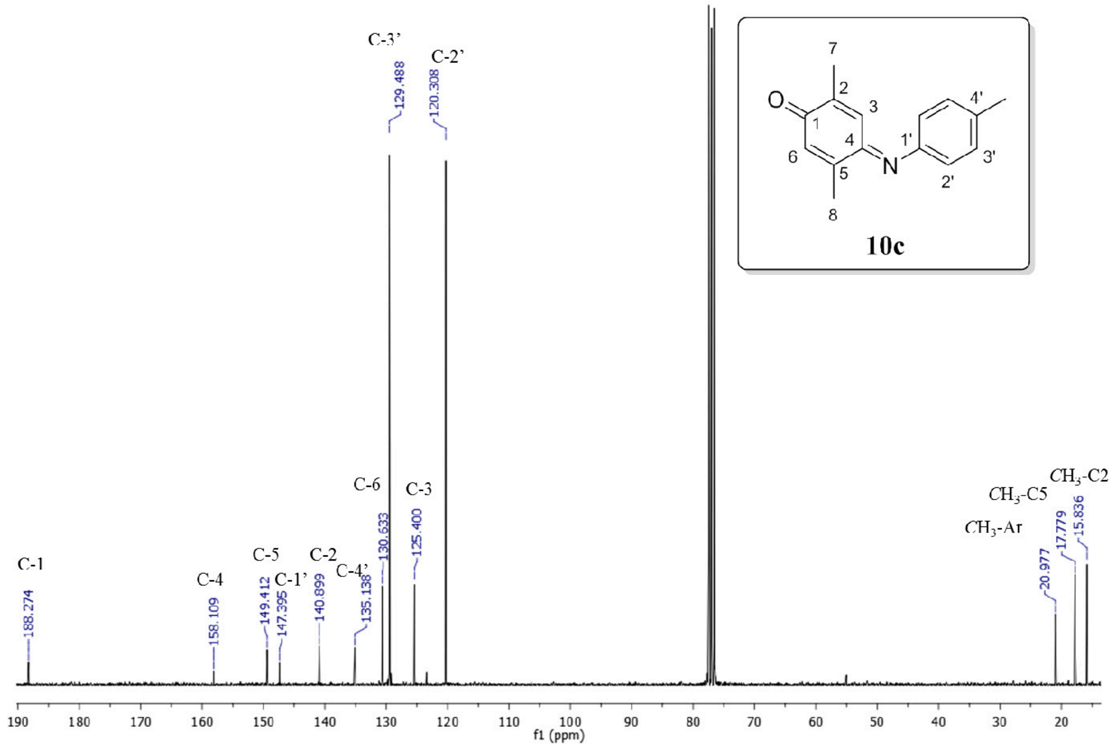

^{13}C-NMR ($CDCl_3$, 75 MHz) spectrum of **10c**.

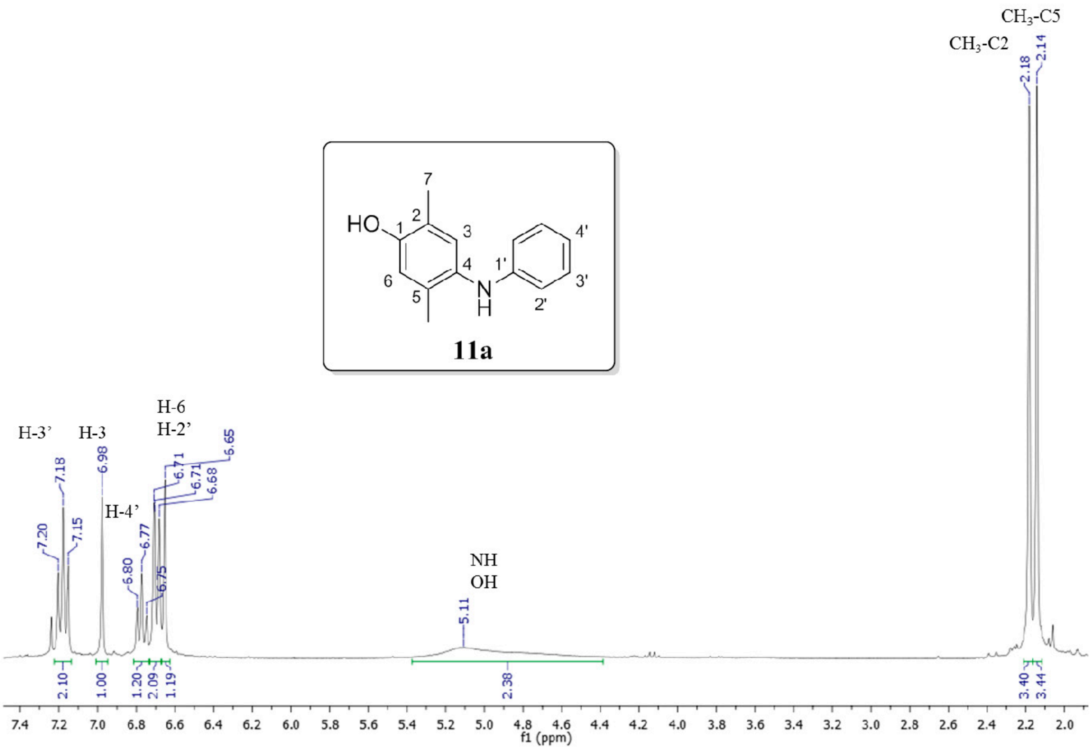

^{1}H-NMR ($CDCl_3$, 300 MHz) spectrum of **11a**

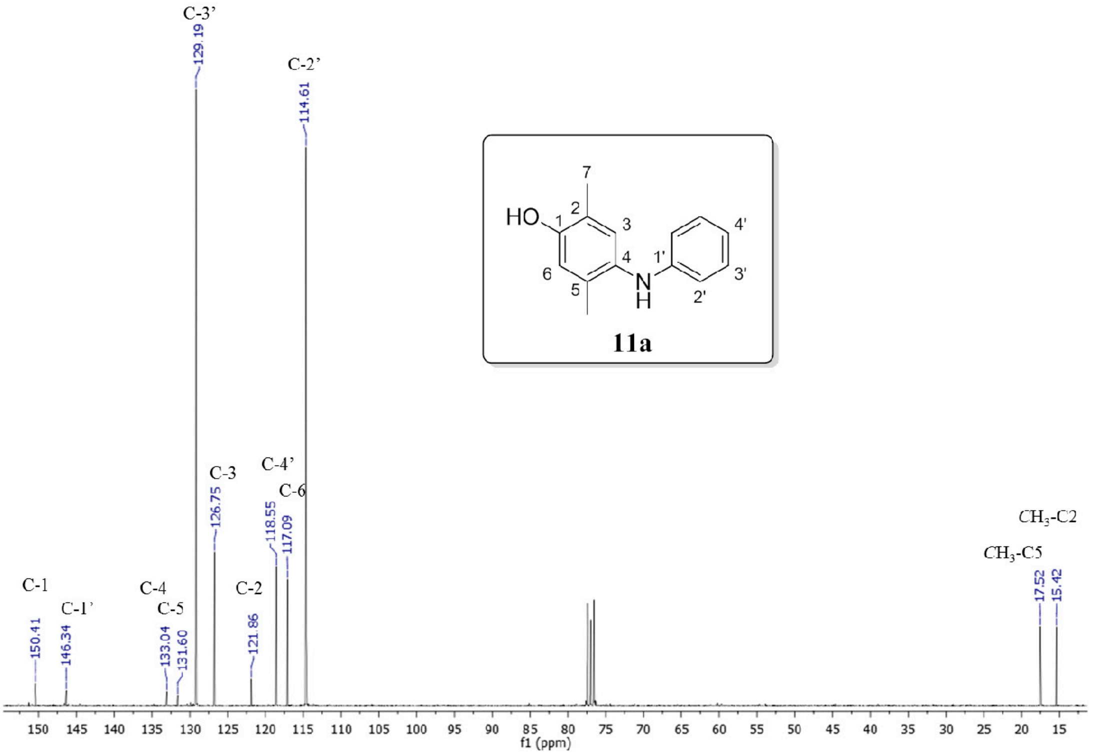

^{13}C-NMR ($CDCl_3$, 75 MHz) spectrum of **11a**

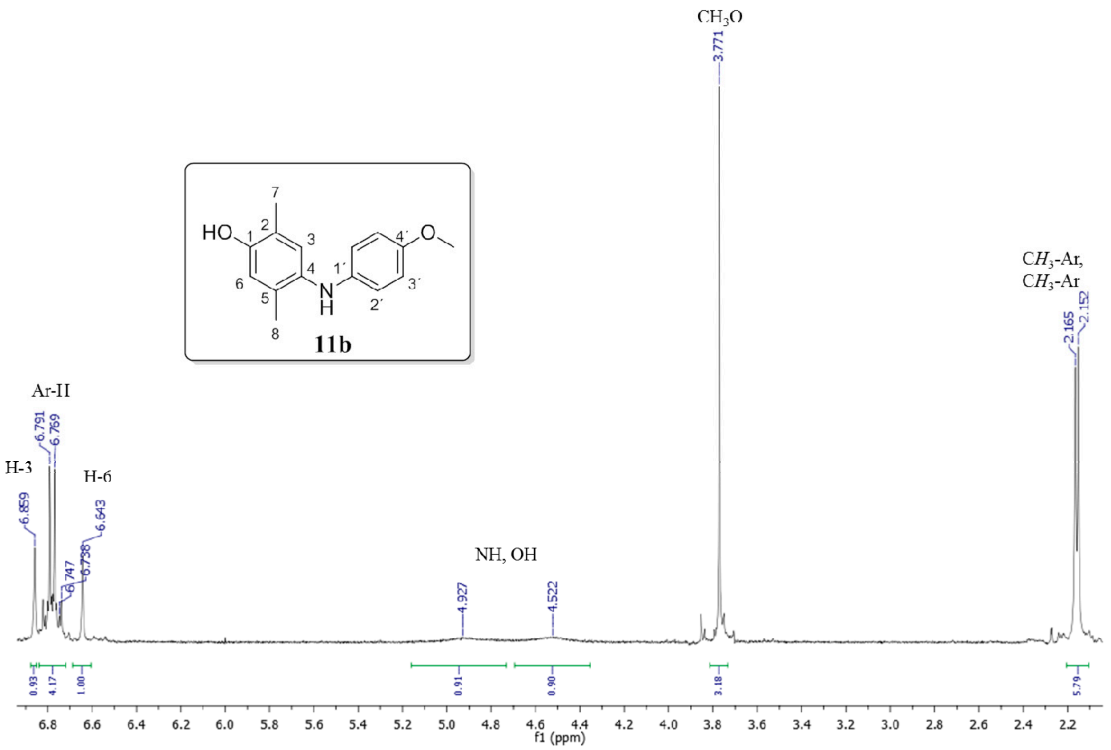

^{1}H-NMR ($CDCl_3$, 300 MHz) spectrum of **11b**

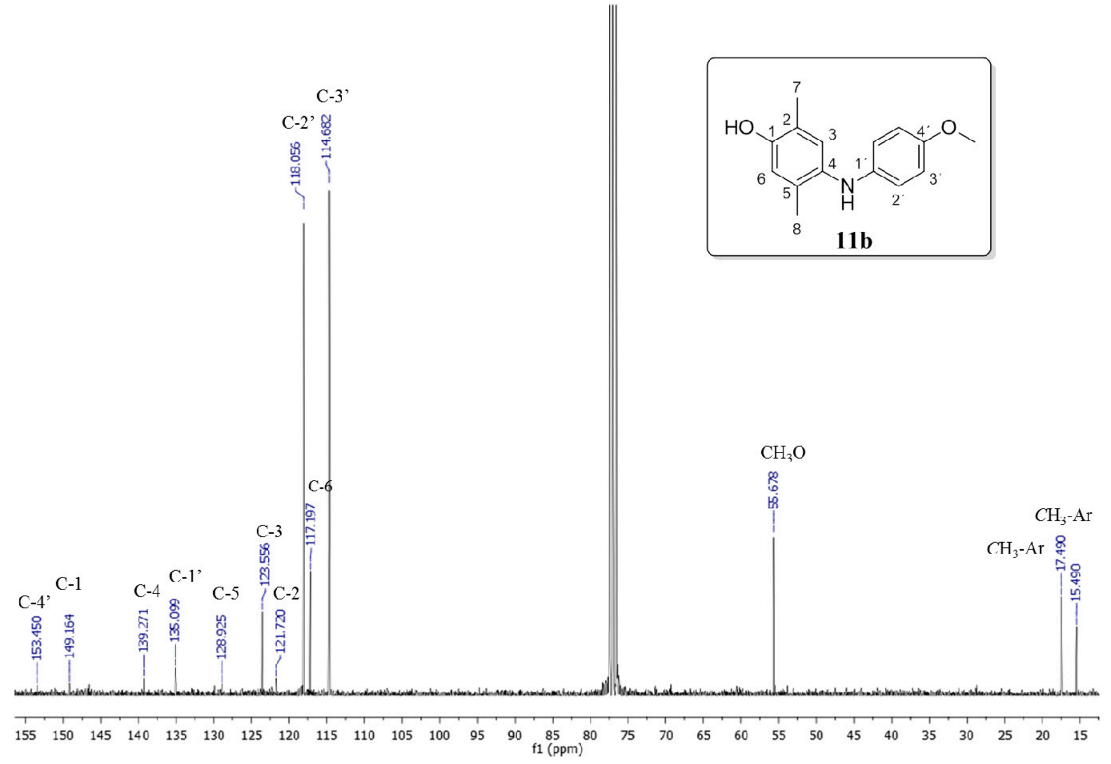

^{13}C-NMR ($CDCl_3$, 75 MHz) spectrum of **11b**

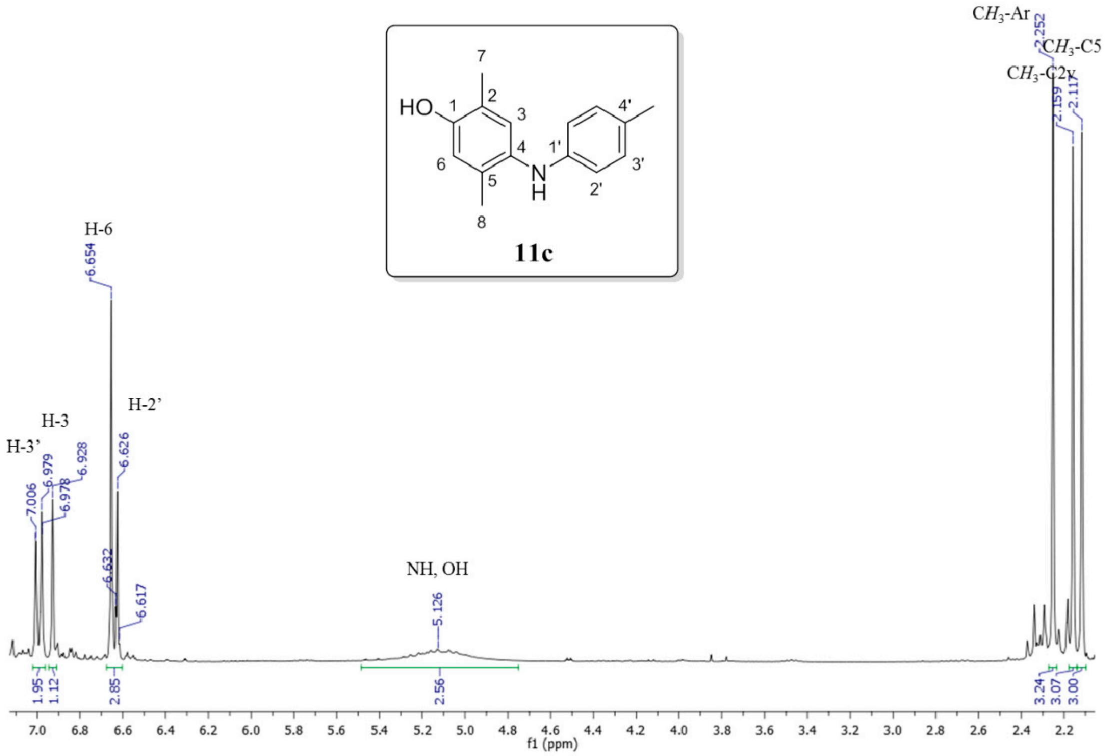

^{1}H-NMR ($CDCl_3$, 300 MHz) spectrum of **11c**.

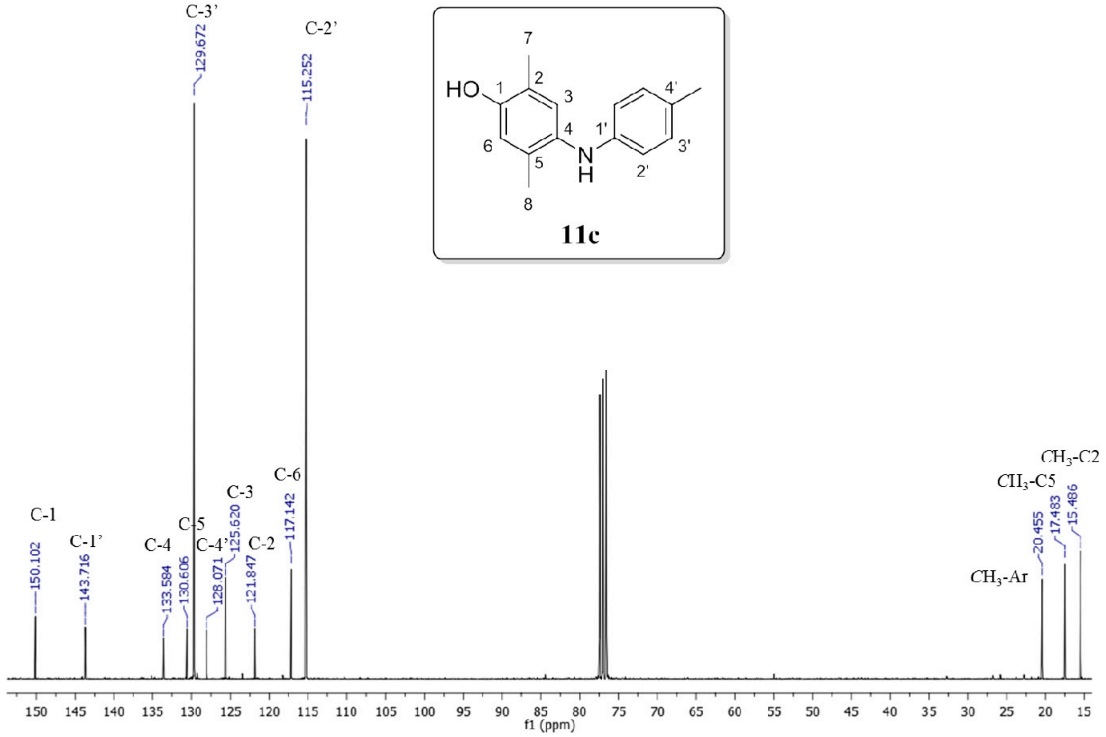

^{13}C-NMR ($CDCl_3$, 75 MHz) spectrum of **11c**.

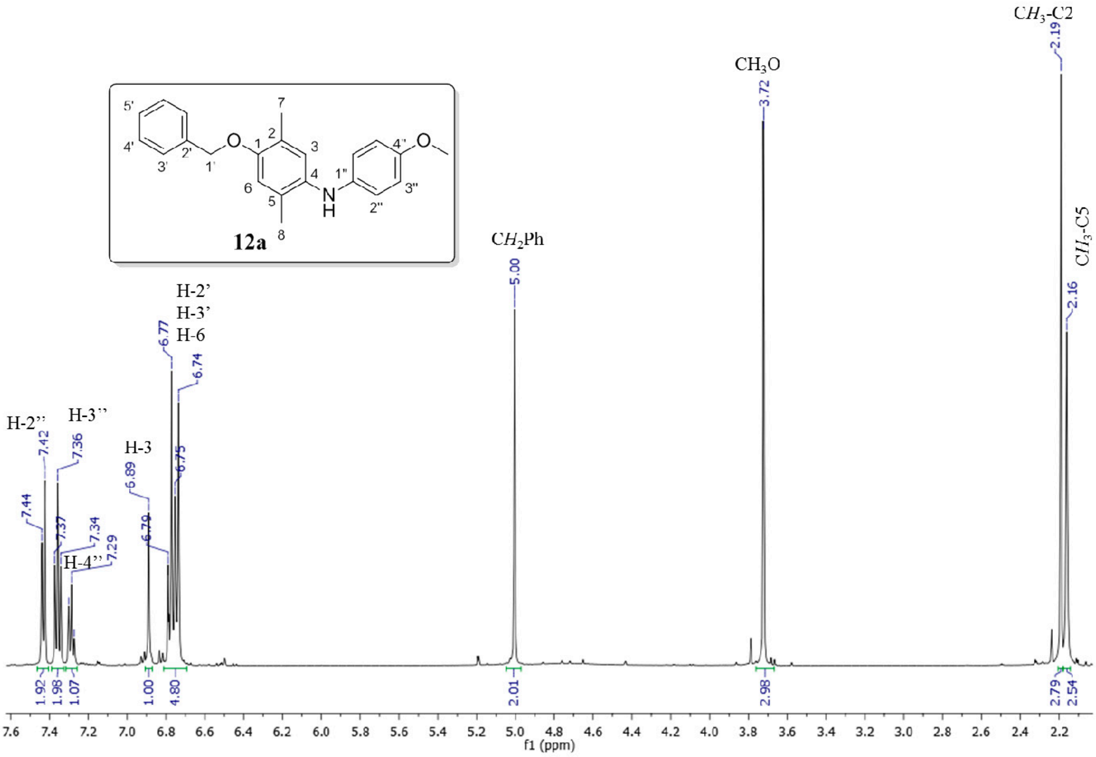

^{1}H-NMR ($CDCl_3$, 500 MHz) spectrum of **12a**

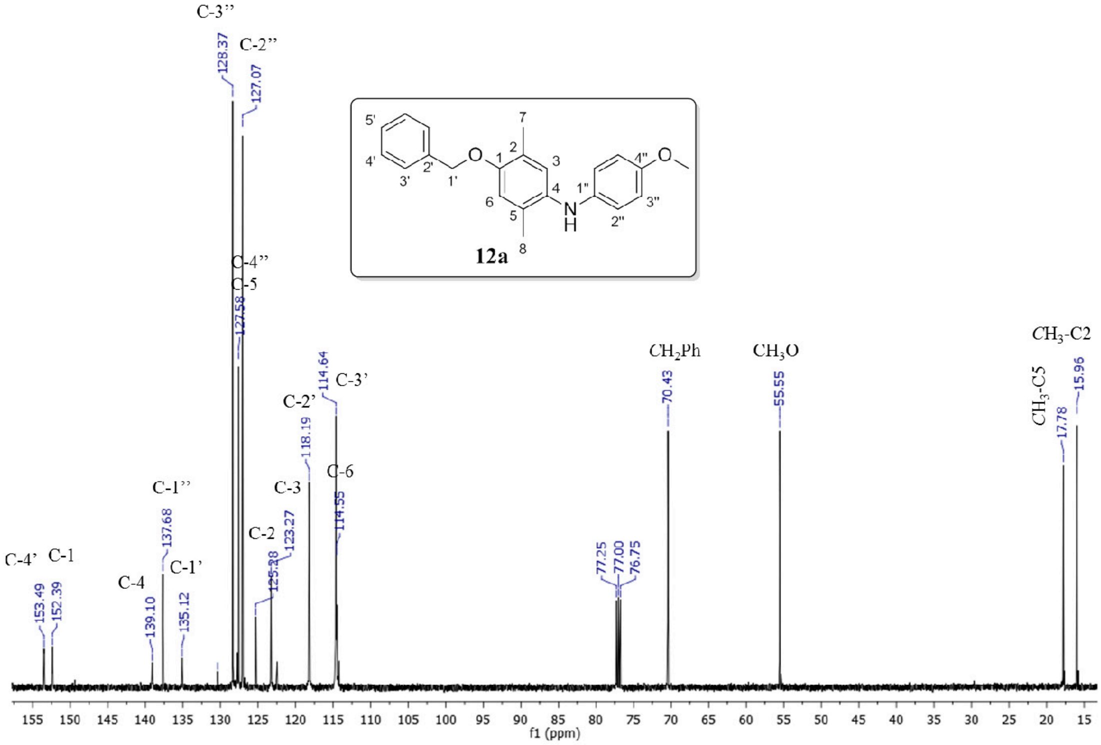

^{13}C-NMR ($CDCl_3$, 125 MHz) spectrum of **12a**

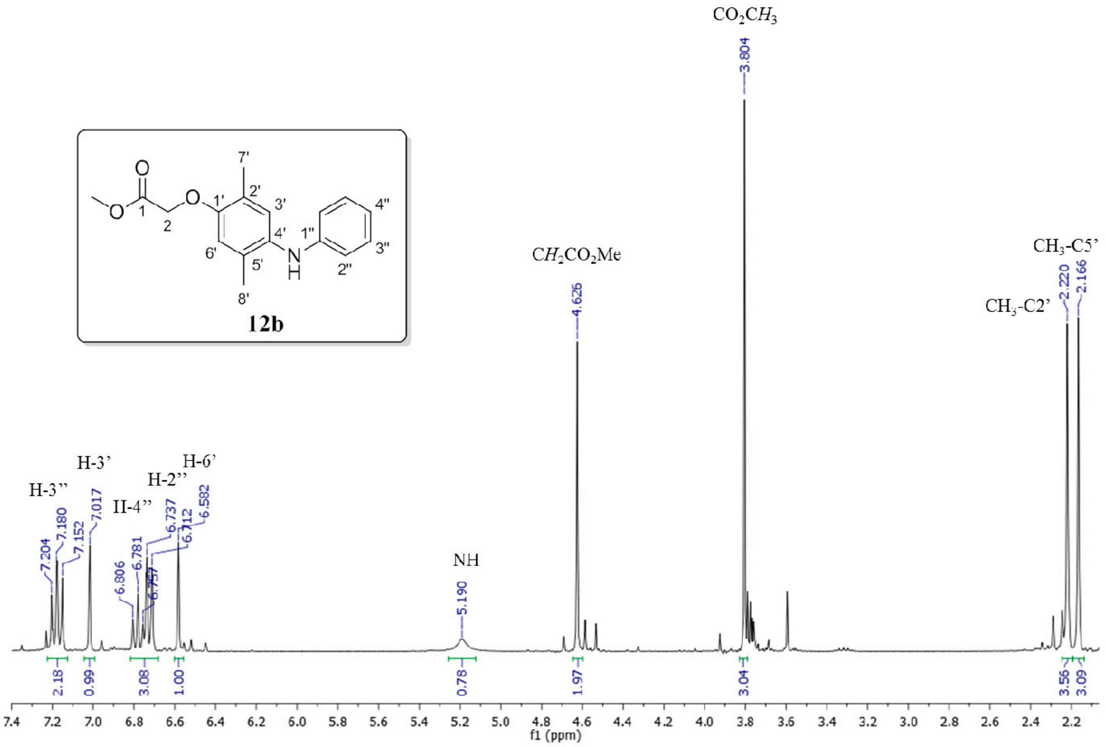

^{1}H-NMR ($CDCl_3$, 300 MHz) spectrum of **12b**

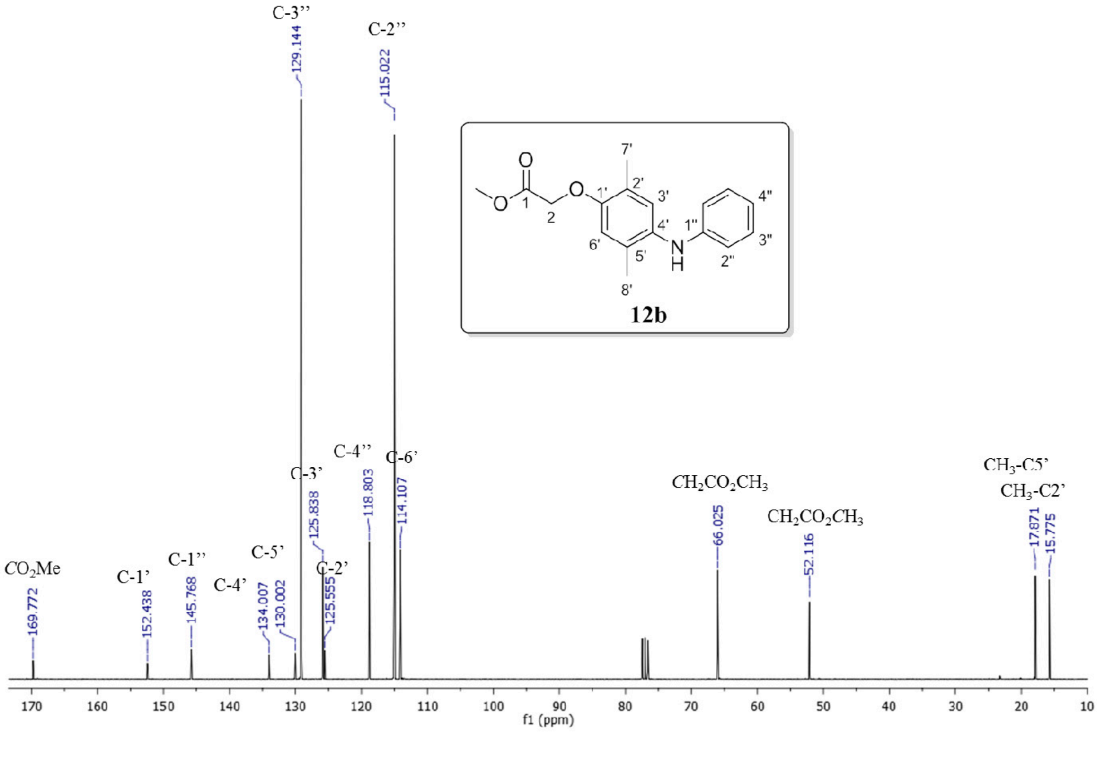

^{13}C-NMR ($CDCl_3$, 75 MHz) spectrum of **12b**

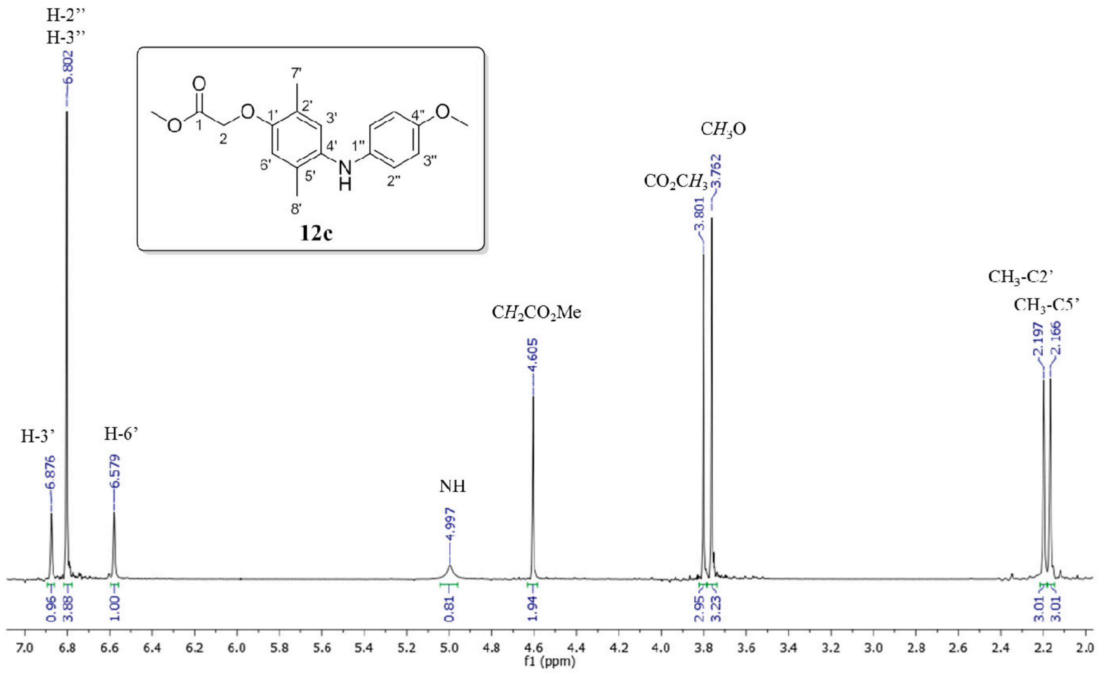

^{1}H-NMR ($CDCl_3$, 300 MHz) spectrum of **12c**

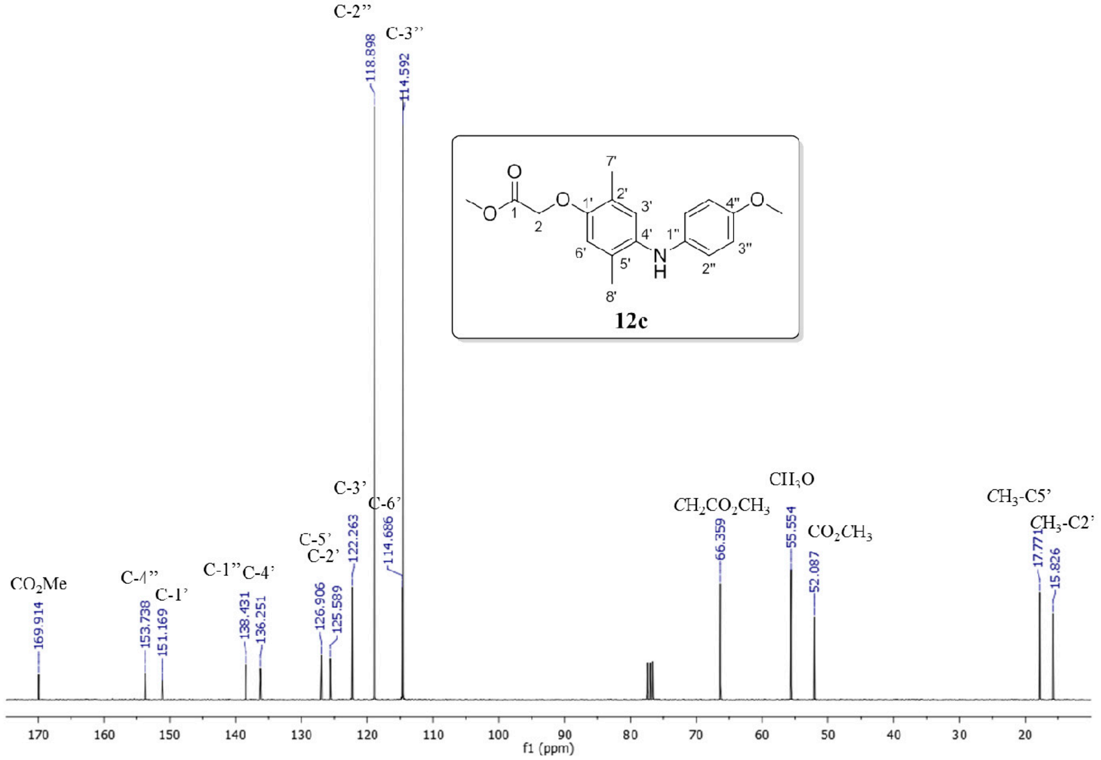

^{13}C-NMR ($CDCl_3$, 75 MHz) spectrum of **12c**

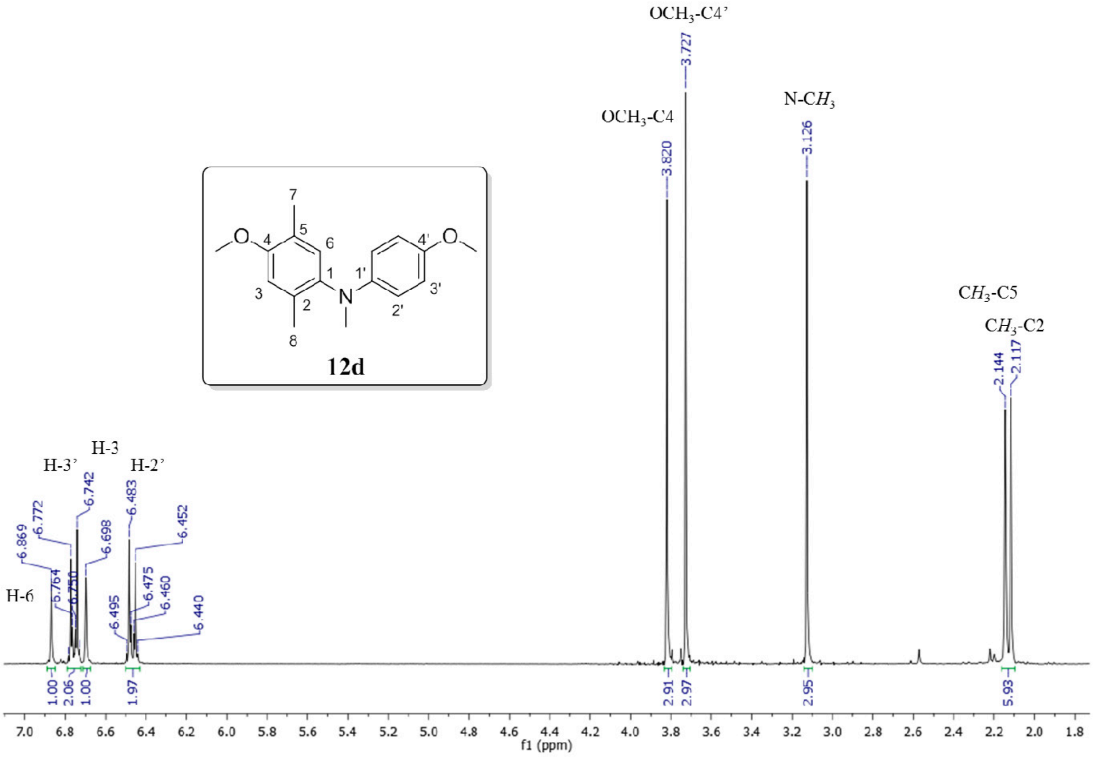

^{1}H-NMR ($CDCl_3$, 500 MHz) spectrum of **12d**

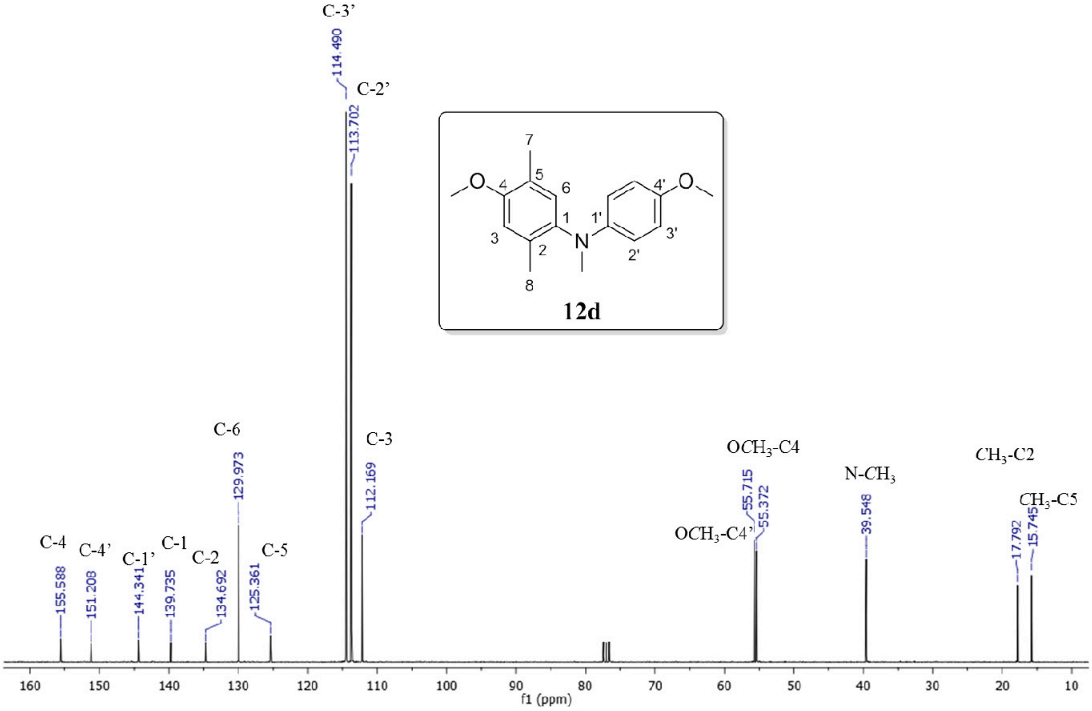

^{13}C-NMR ($CDCl_3$, 125 MHz) spectrum of **12d**

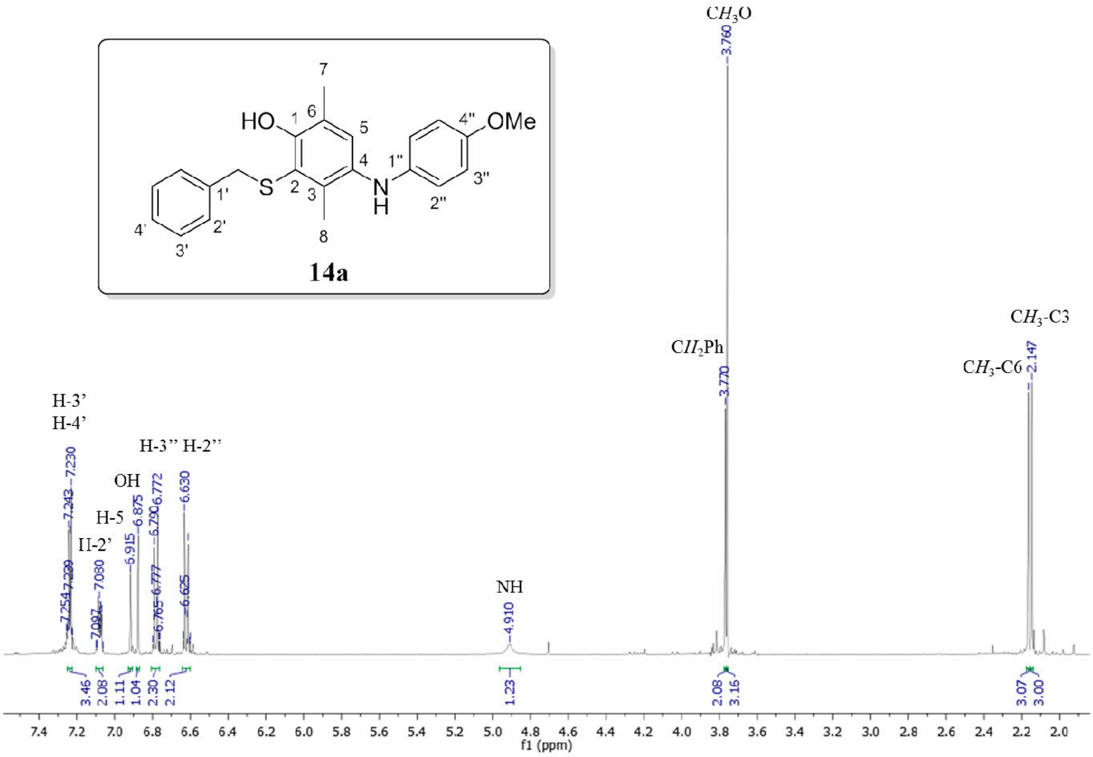

^{1}H-NMR ($CDCl_3$, 500 MHz) spectrum of **14a**

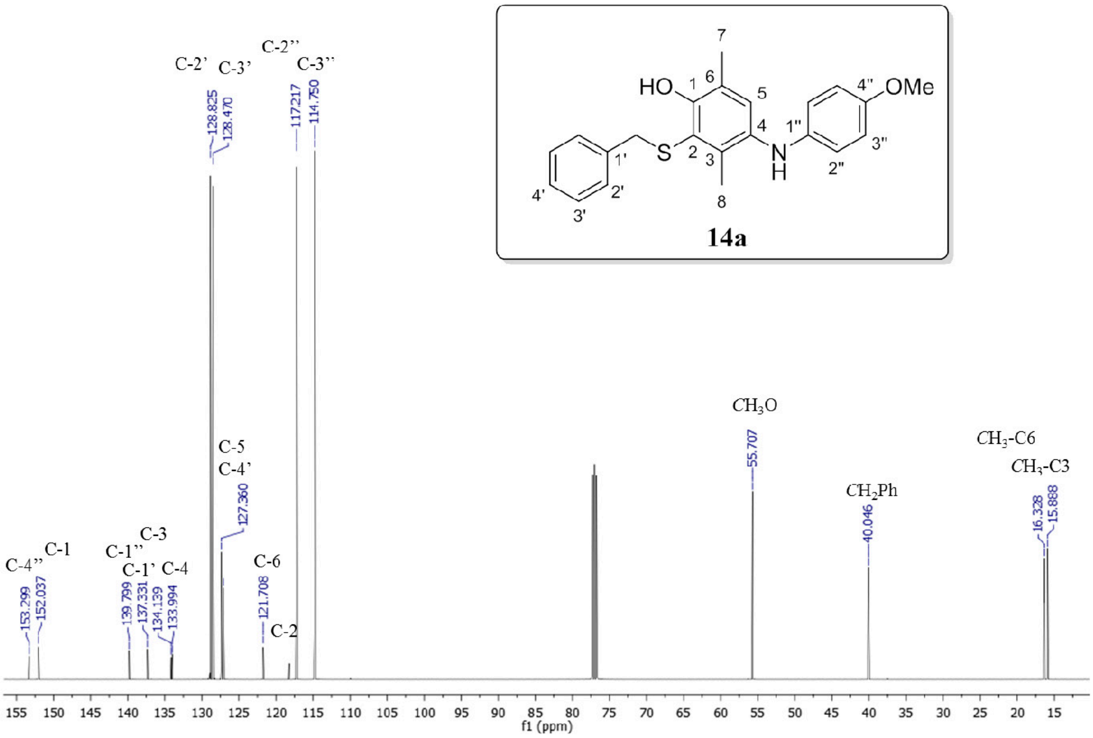

^{13}C-NMR ($CDCl_3$, 125 MHz) spectrum of **14a**

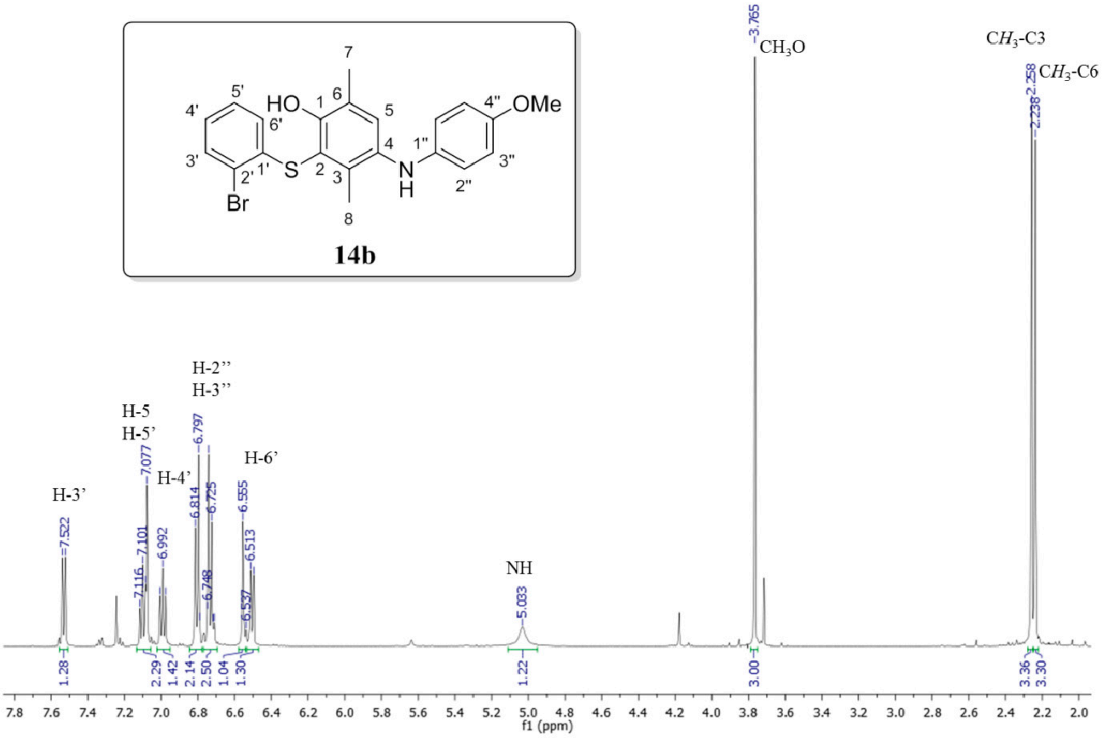

^{1}H-NMR ($CDCl_3$, 500 MHz) spectrum of **14b**

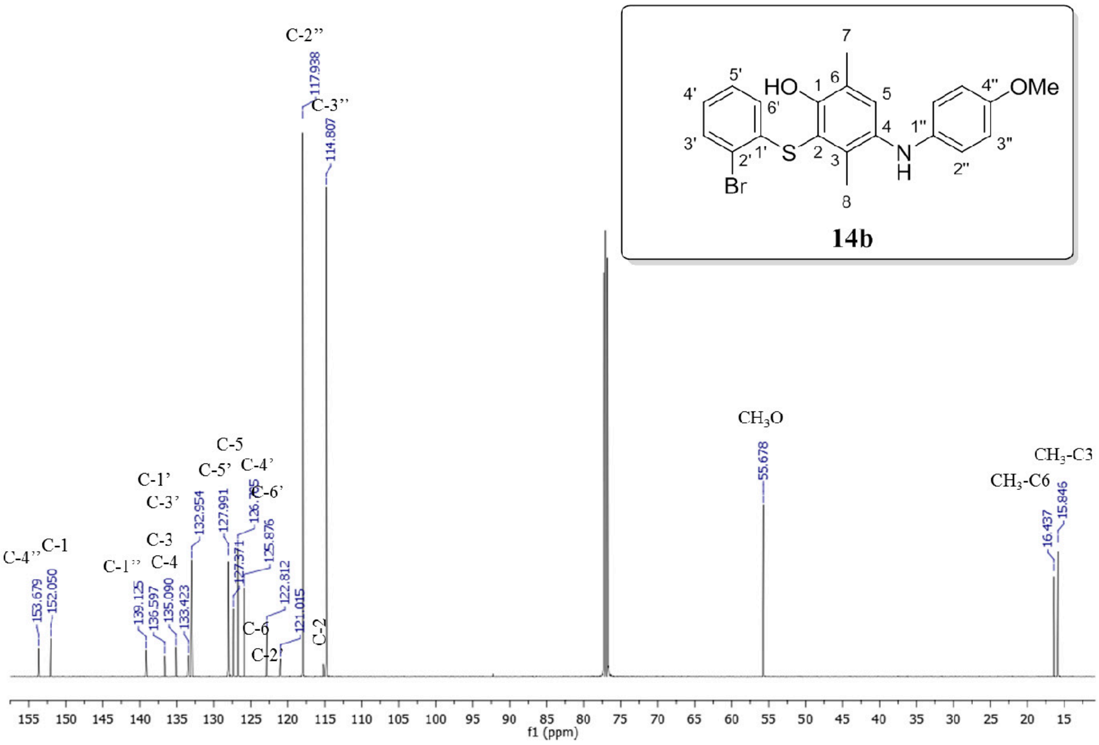

^{13}C-NMR ($CDCl_3$, 125 MHz) spectrum of **14b**

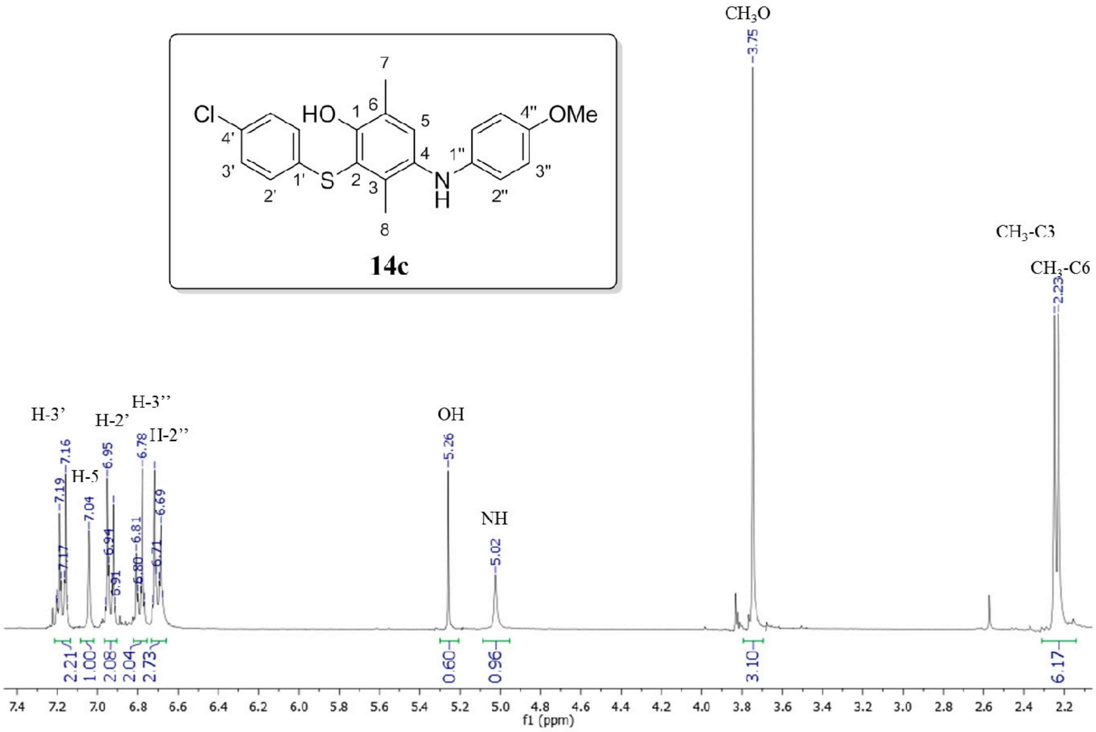

^{1}H-NMR ($CDCl_3$, 300 MHz) spectrum of **14c**

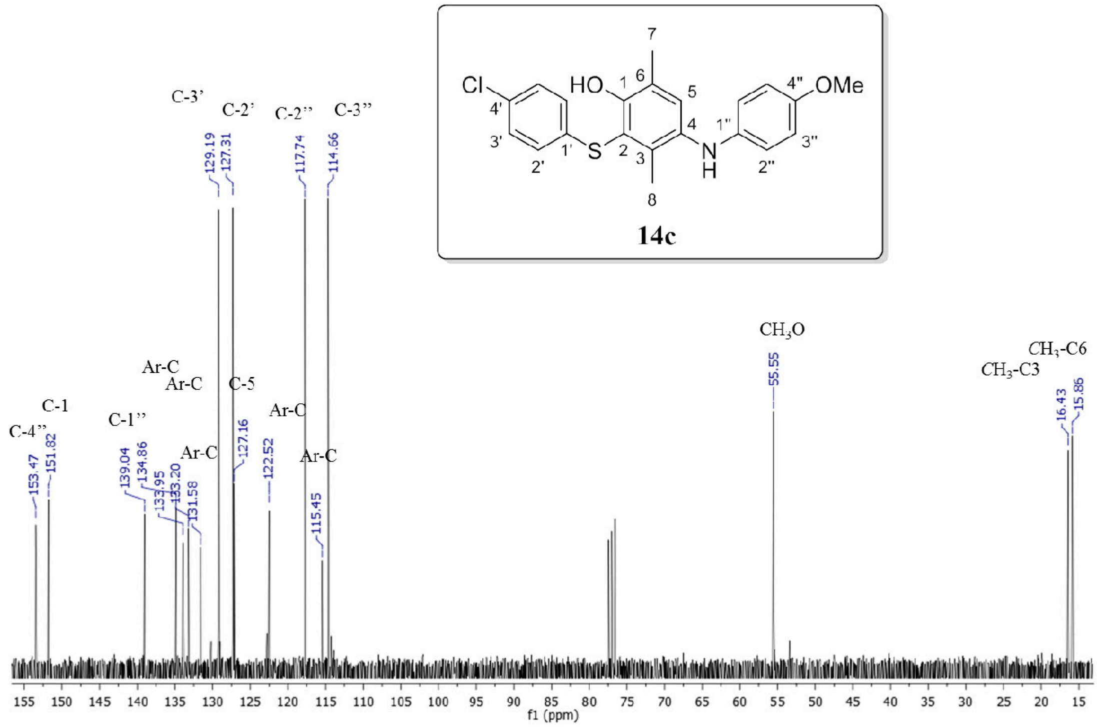

^{13}C-NMR ($CDCl_3$, 75 MHz) spectrum of **14c**

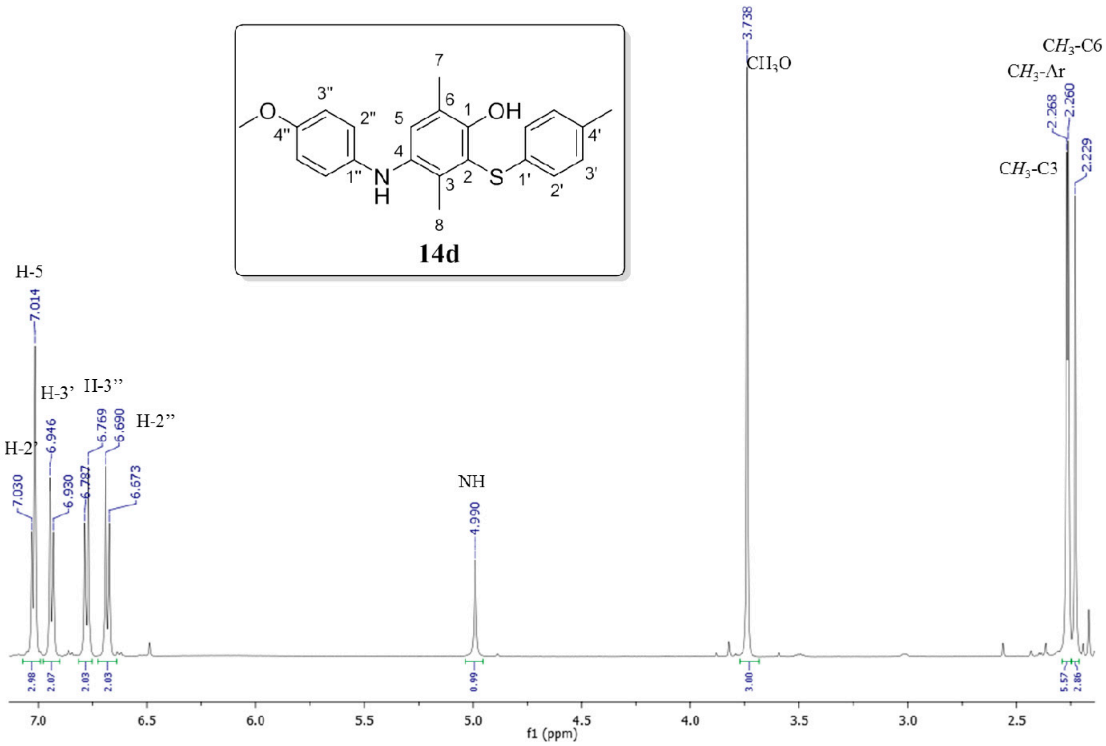

^{1}H-NMR ($CDCl_3$, 500 MHz) spectrum of **14d**.

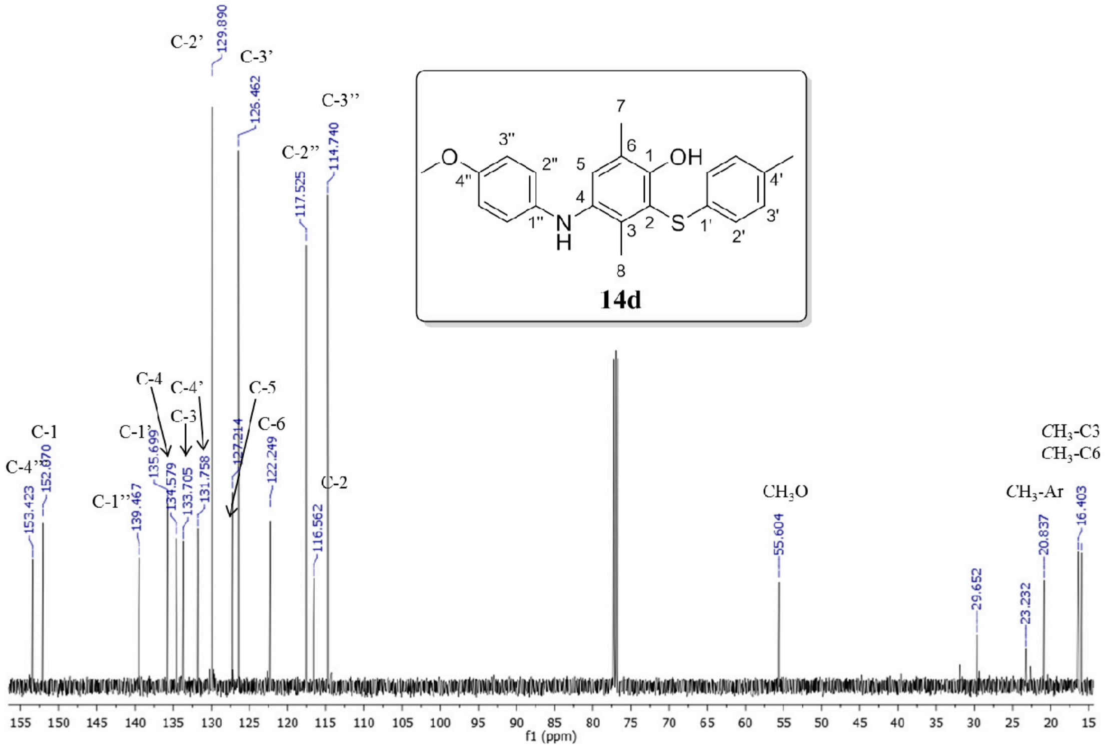

^{13}C-NMR ($CDCl_3$, 125 MHz) spectrum of **14d**.

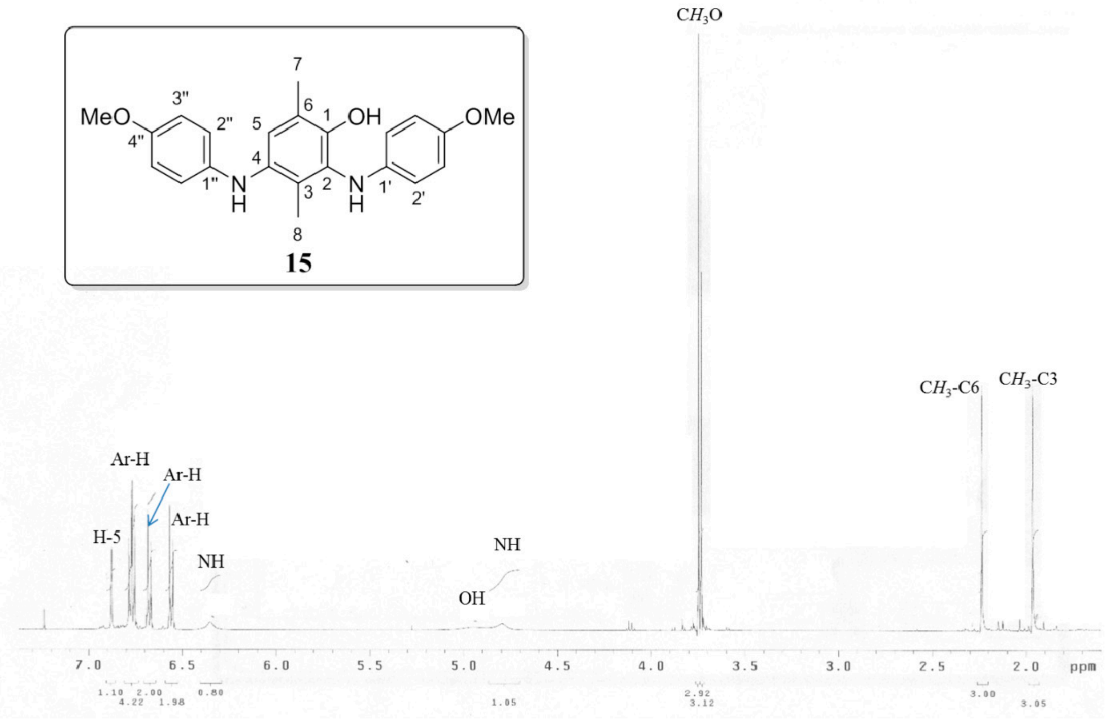

^{1}H-NMR (CDCl$_3$, 500 MHz) spectrum of **15**.

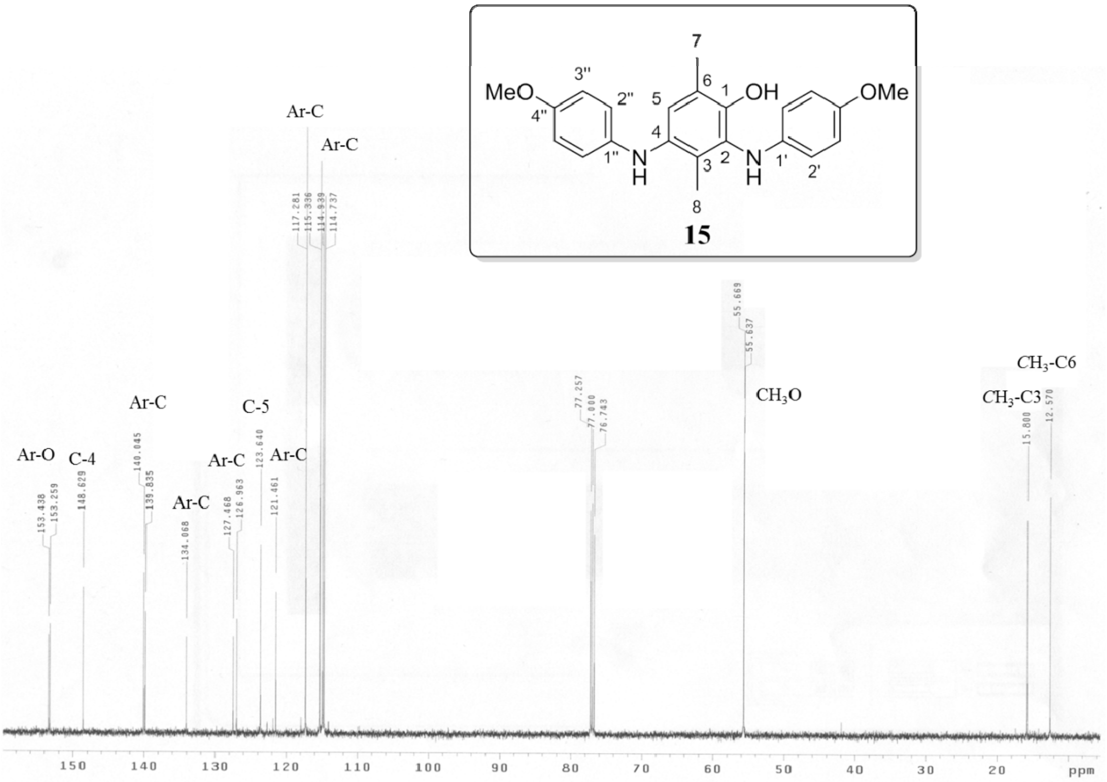

^{13}C-NMR (CDCl$_3$, 125 MHz) spectrum of **15**.

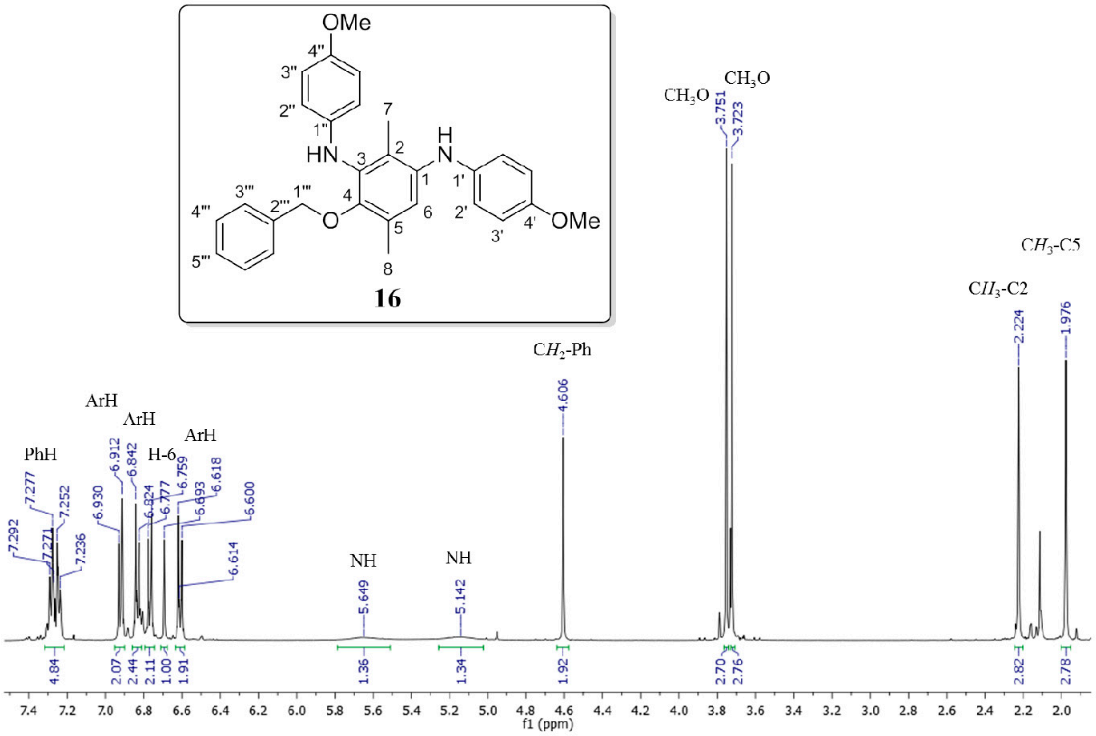

^{1}H-NMR ($CDCl_3$, 300 MHz) spectrum of **16**

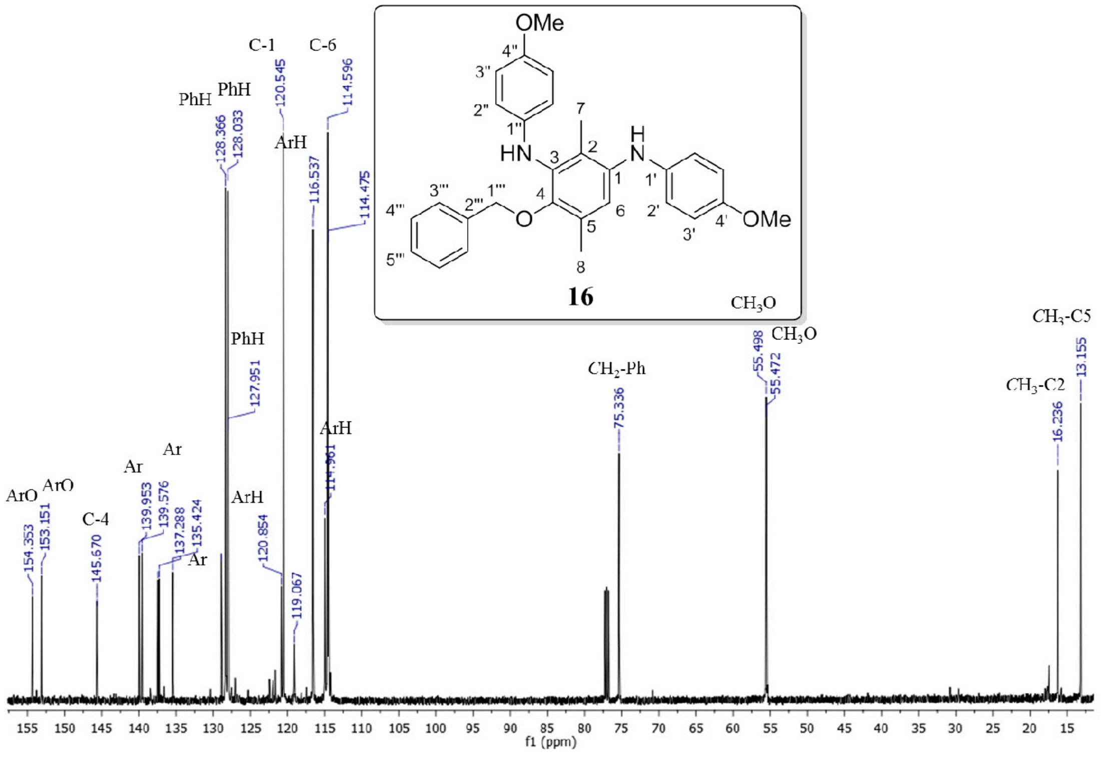

^{13}C-NMR ($CDCl_3$, 75 MHz) spectrum of **16**

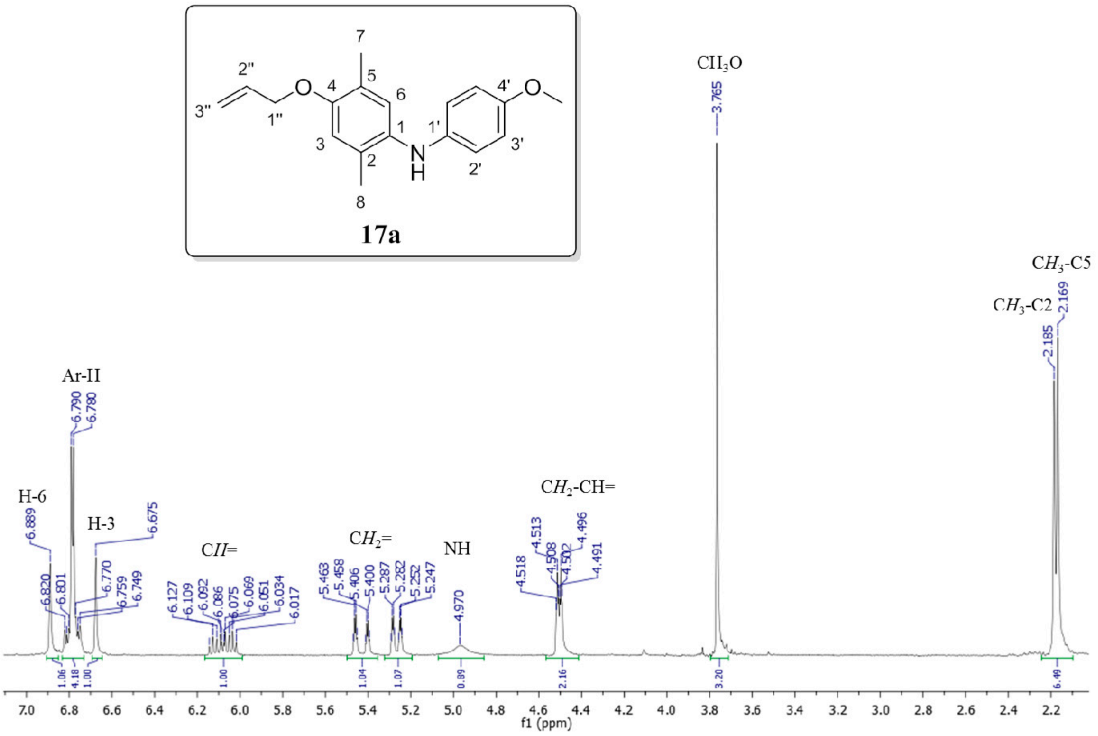

^{1}H-NMR ($CDCl_3$, 500 MHz) spectrum of **17a**.

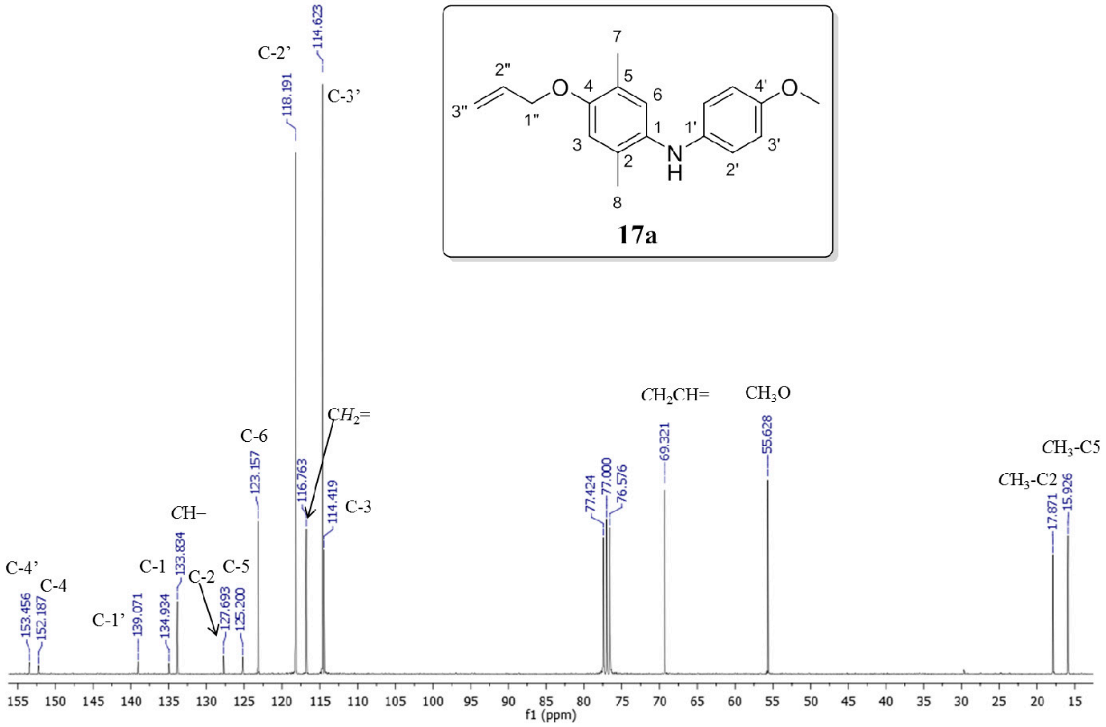

^{13}C-NMR ($CDCl_3$, 500 MHz) spectrum of **17a**.

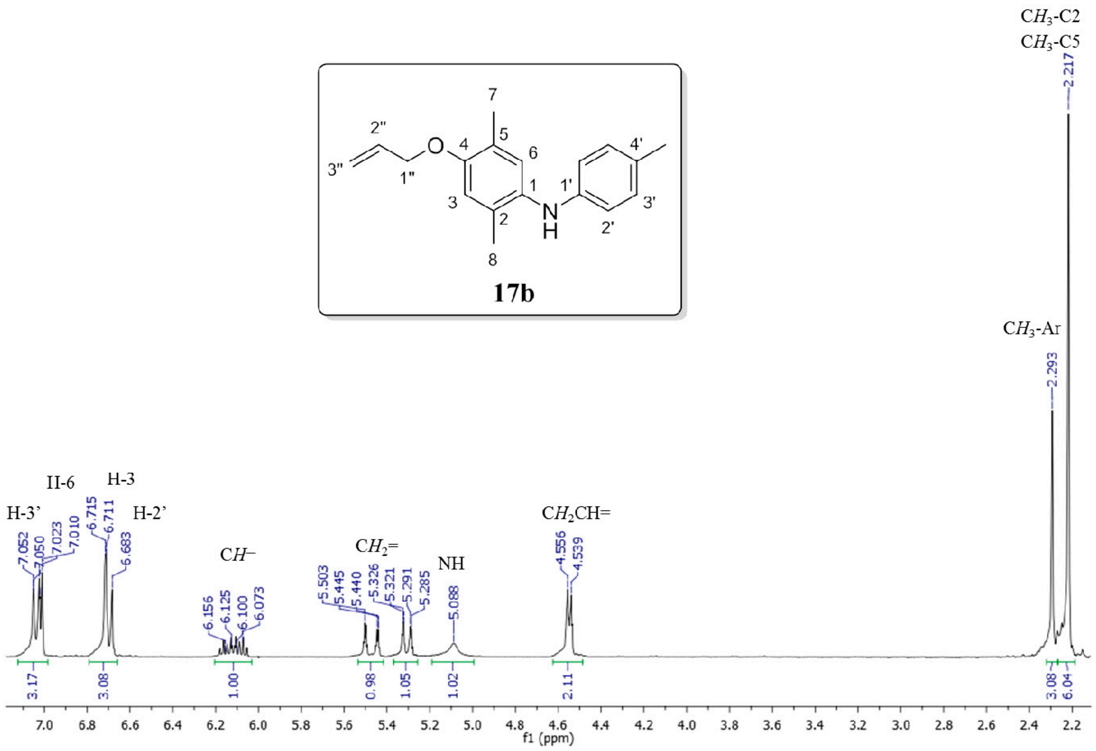

^{1}H-NMR ($CDCl_3$, 300 MHz) spectrum of **17b**.

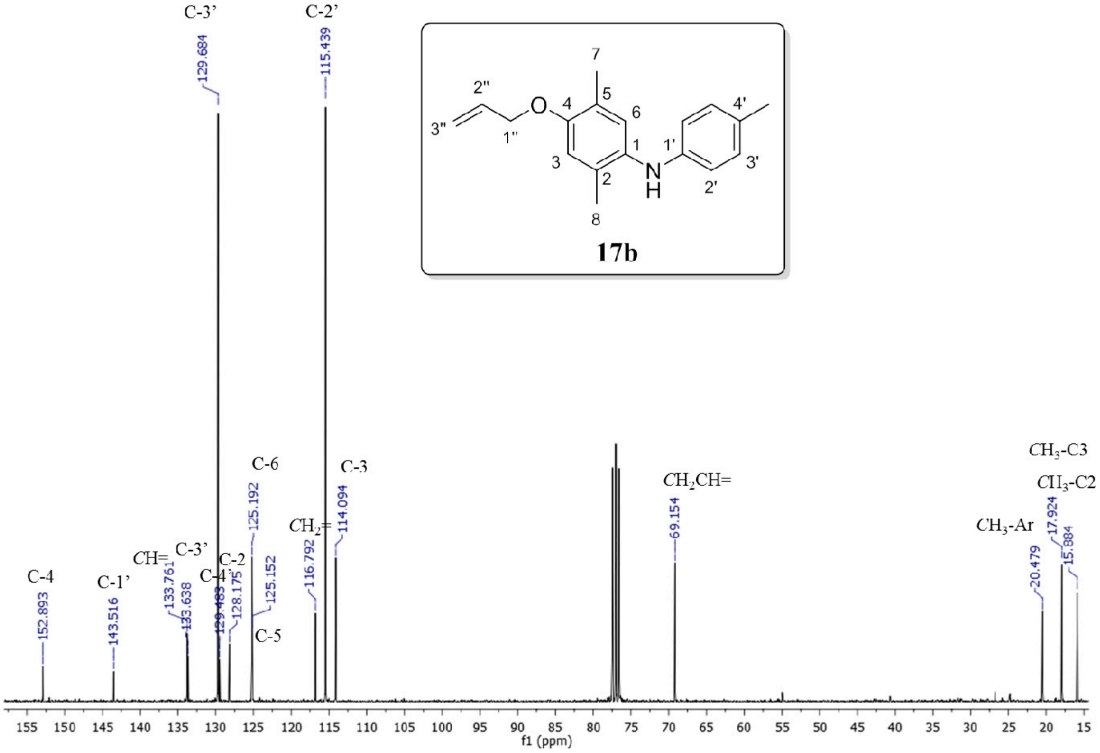

^{13}C-NMR ($CDCl_3$, 75 MHz) spectrum of **17b**.

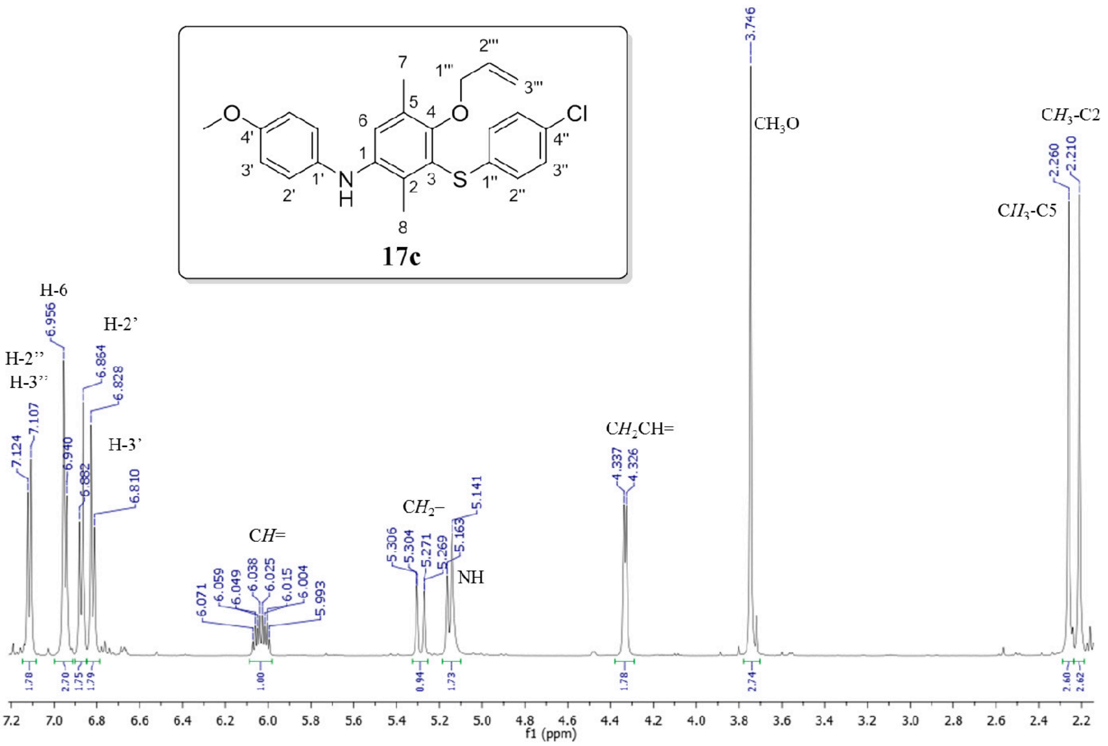

^{1}H-NMR (CDCl$_3$, 500 MHz) spectrum of **17c**.

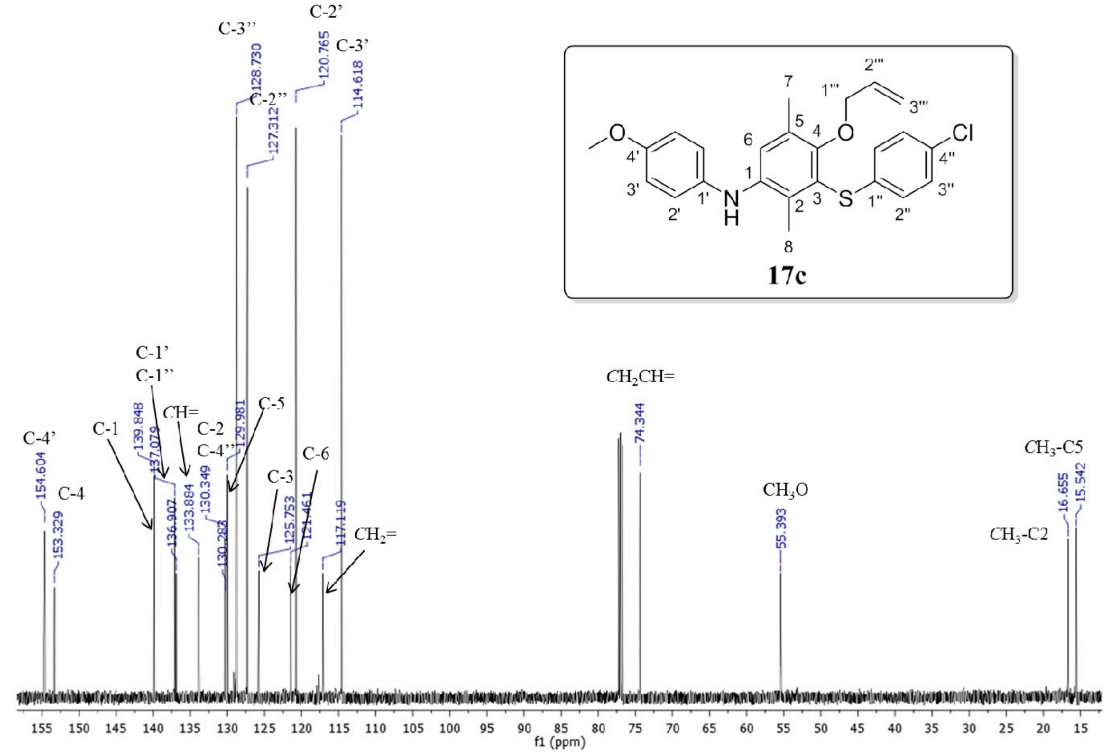

^{13}C-NMR (CDCl$_3$, 125 MHz) spectrum of **17c**.

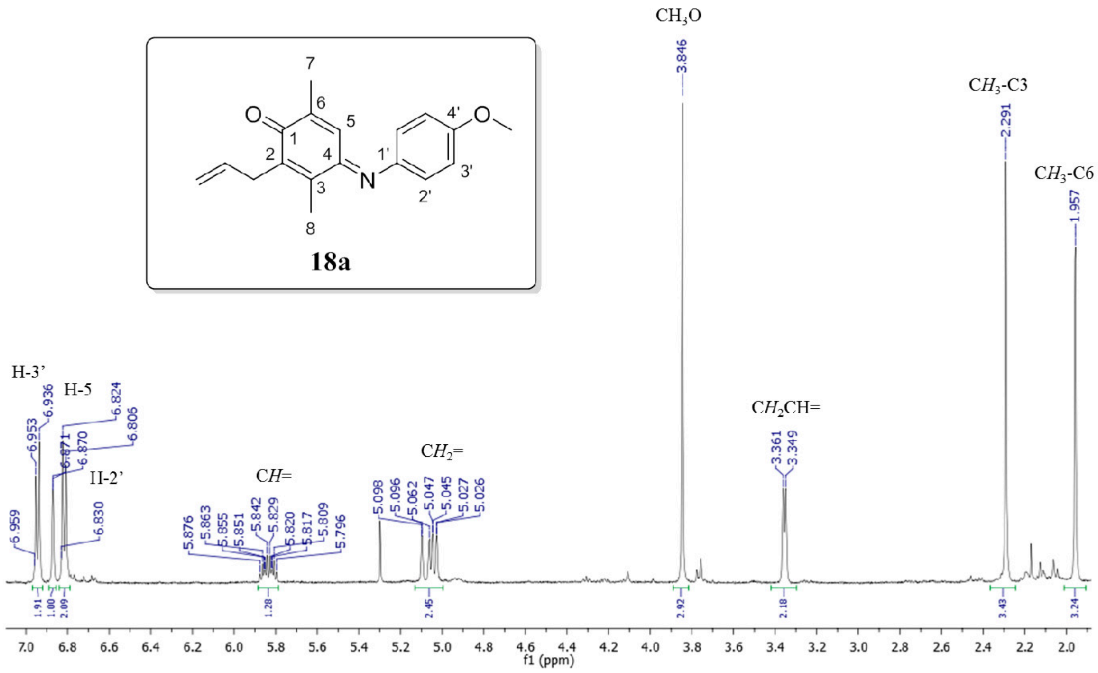

^{1}H-NMR ($CDCl_3$, 500 MHz) spectrum of **18a**.

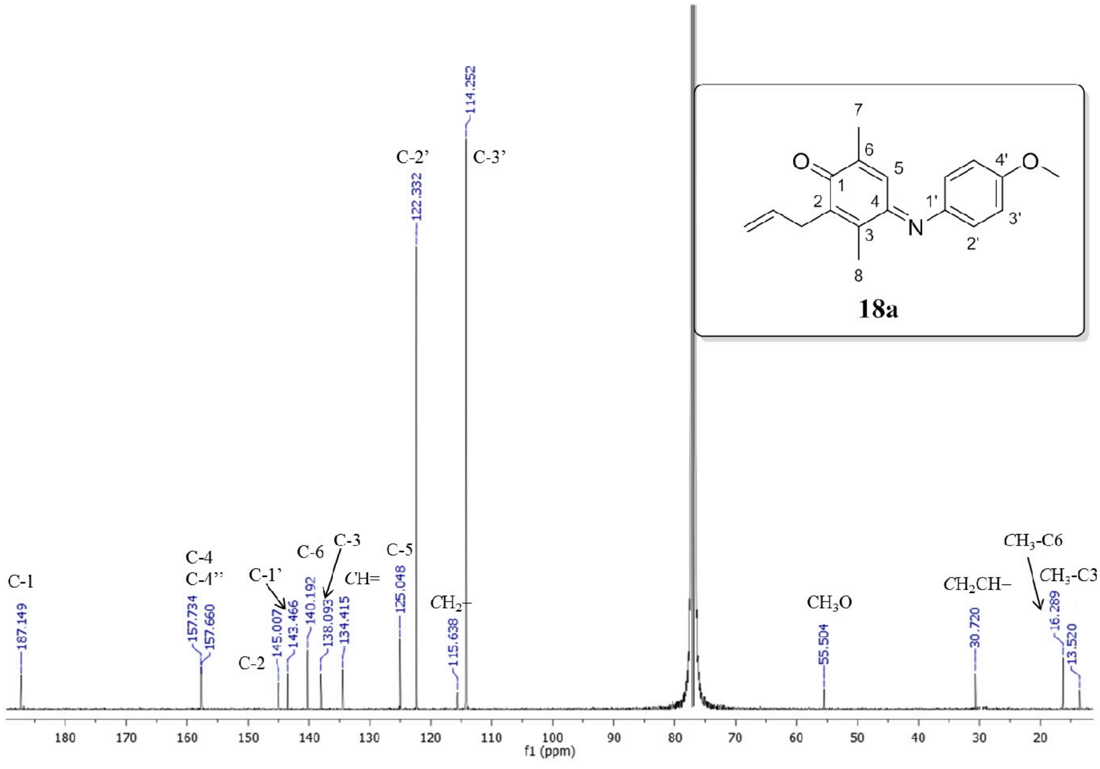

^{13}C-NMR ($CDCl_3$, 500 MHz) spectrum of **18a**.

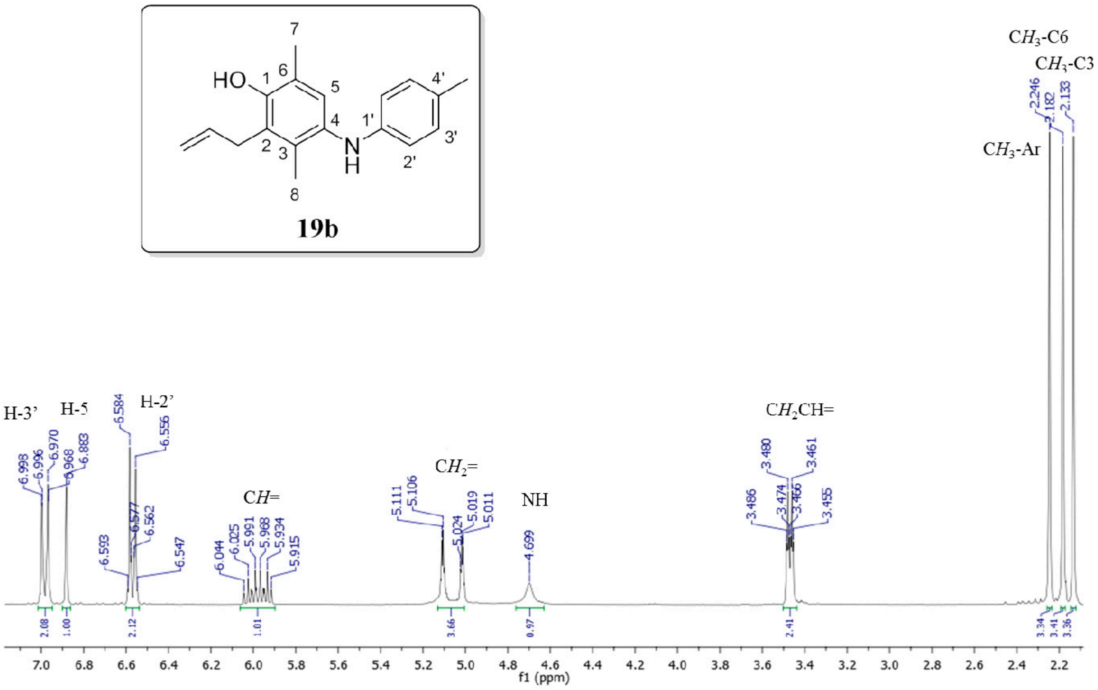

^{1}H-NMR ($CDCl_3$, 300 MHz) spectrum of **19b**.

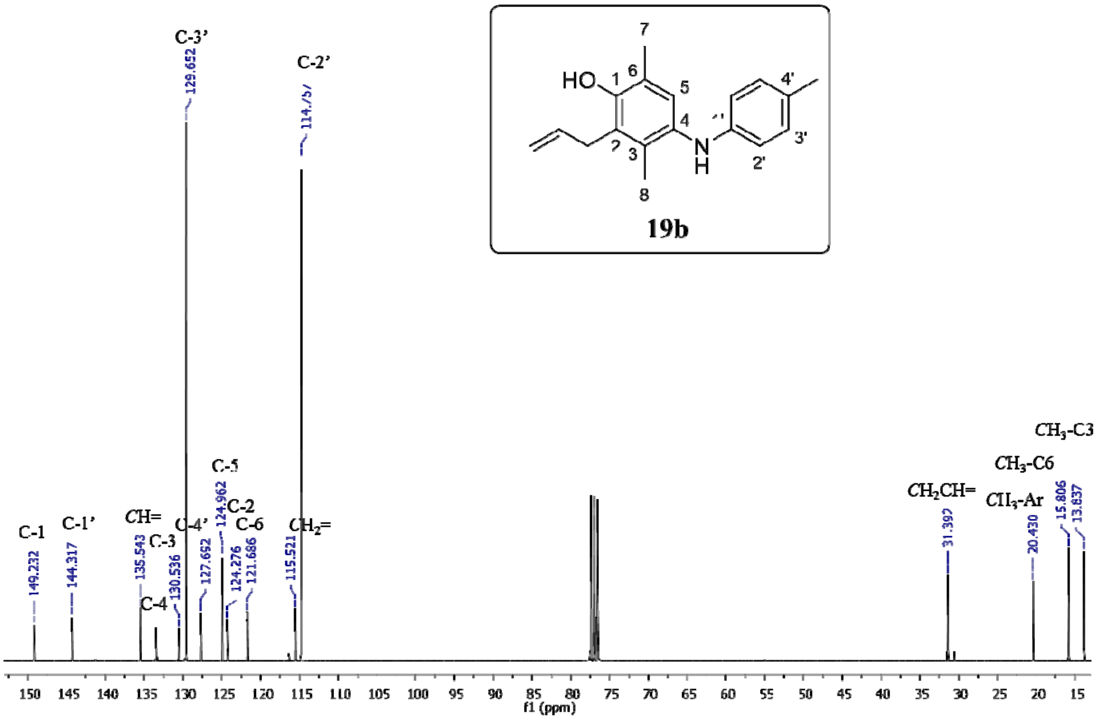

^{13}C-NMR ($CDCl_3$, 75 MHz) spectrum of **19b**.

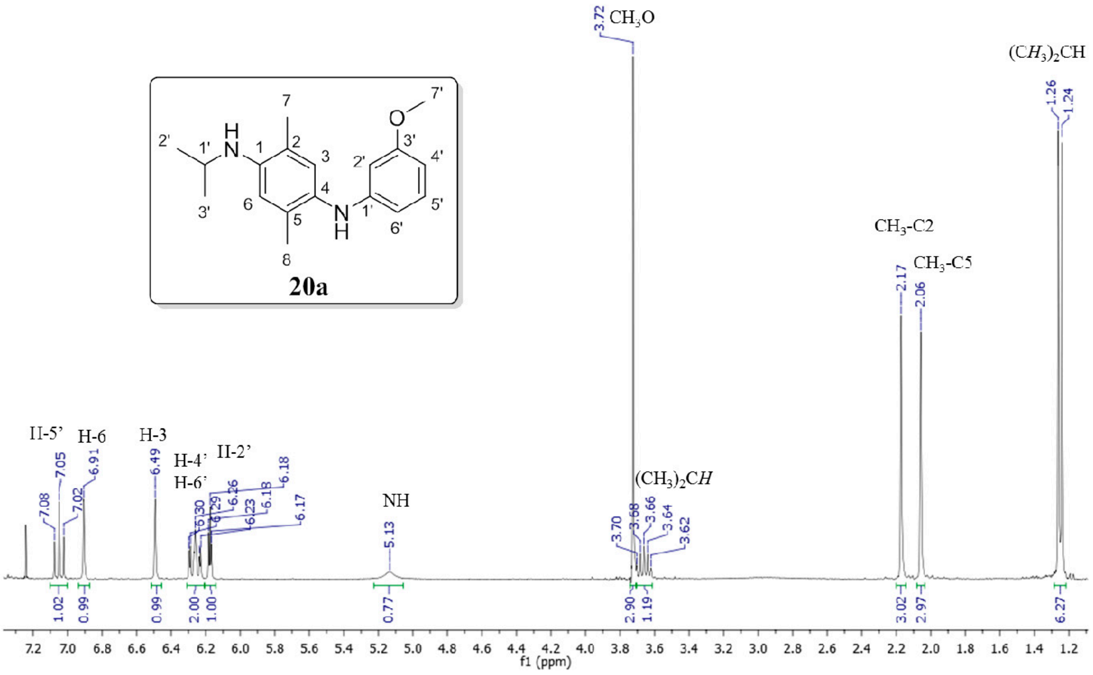

^{1}H-NMR ($CDCl_3$, 300 MHz) spectrum of **20a**

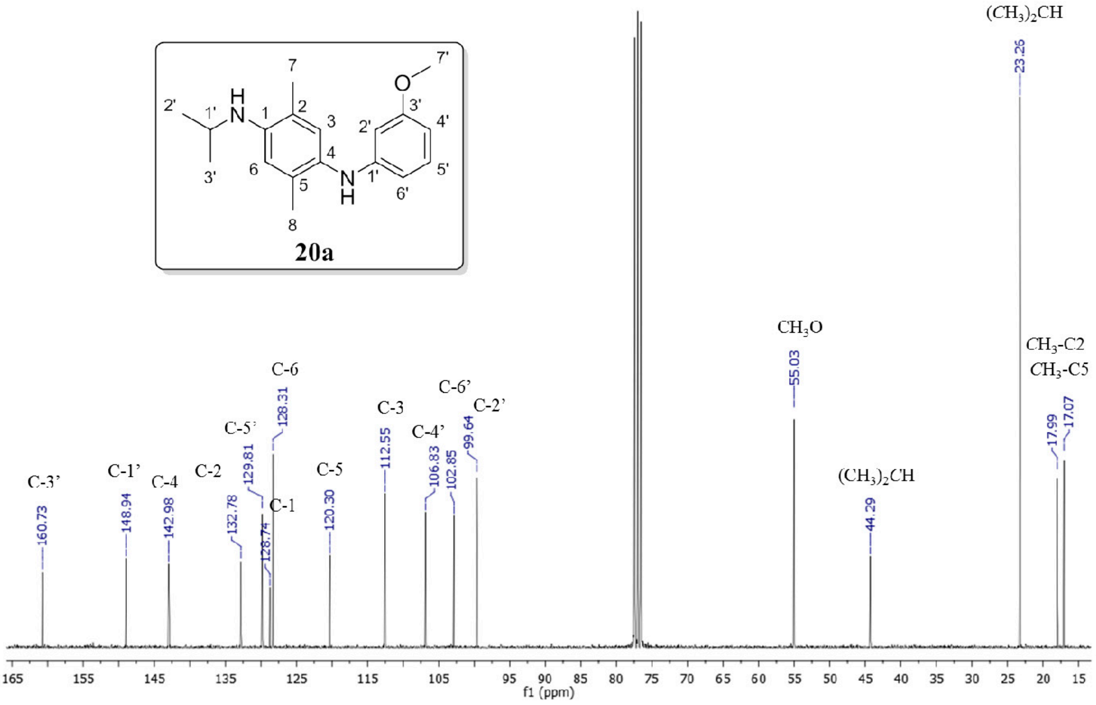

^{13}C-NMR ($CDCl_3$, 75 MHz) spectrum of **20a**

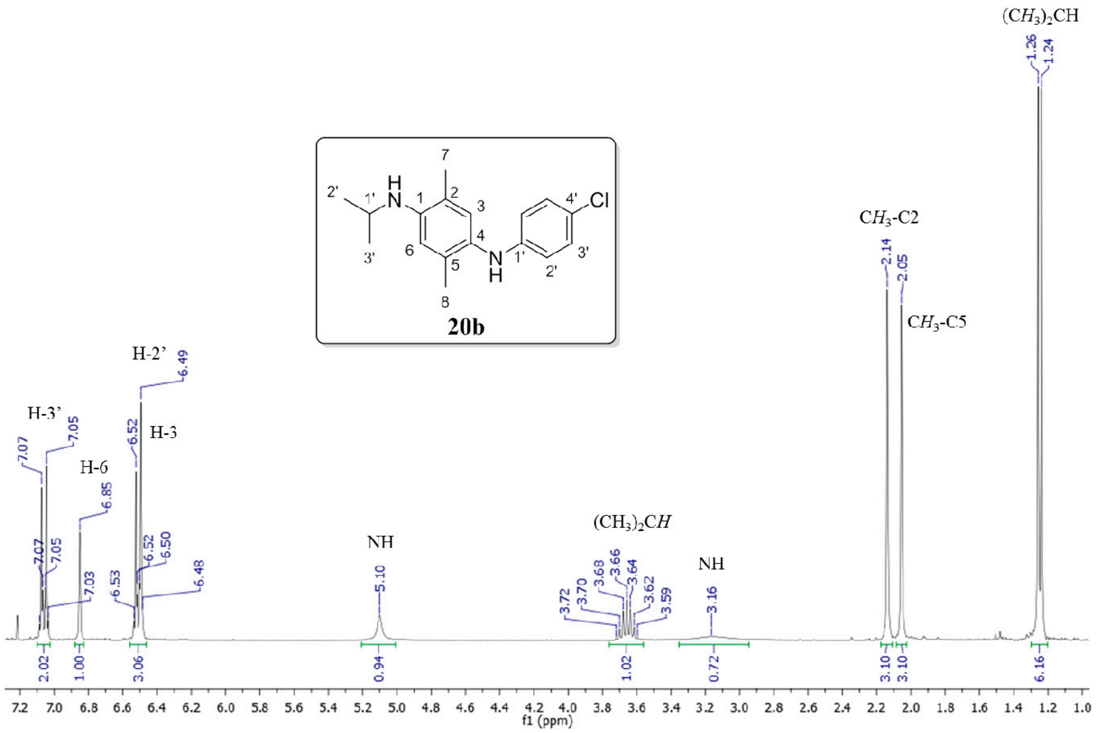

^{1}H-NMR ($CDCl_3$, 300 MHz) spectrum of **20b**

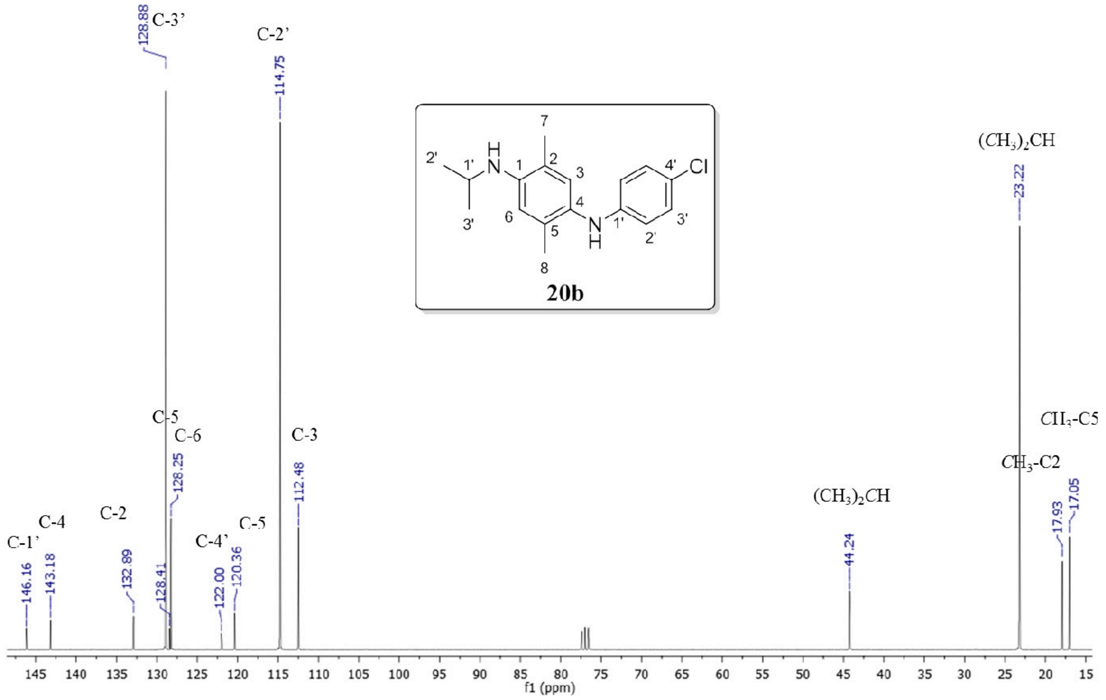

^{13}C-NMR ($CDCl_3$, 75 MHz) spectrum of **20b**

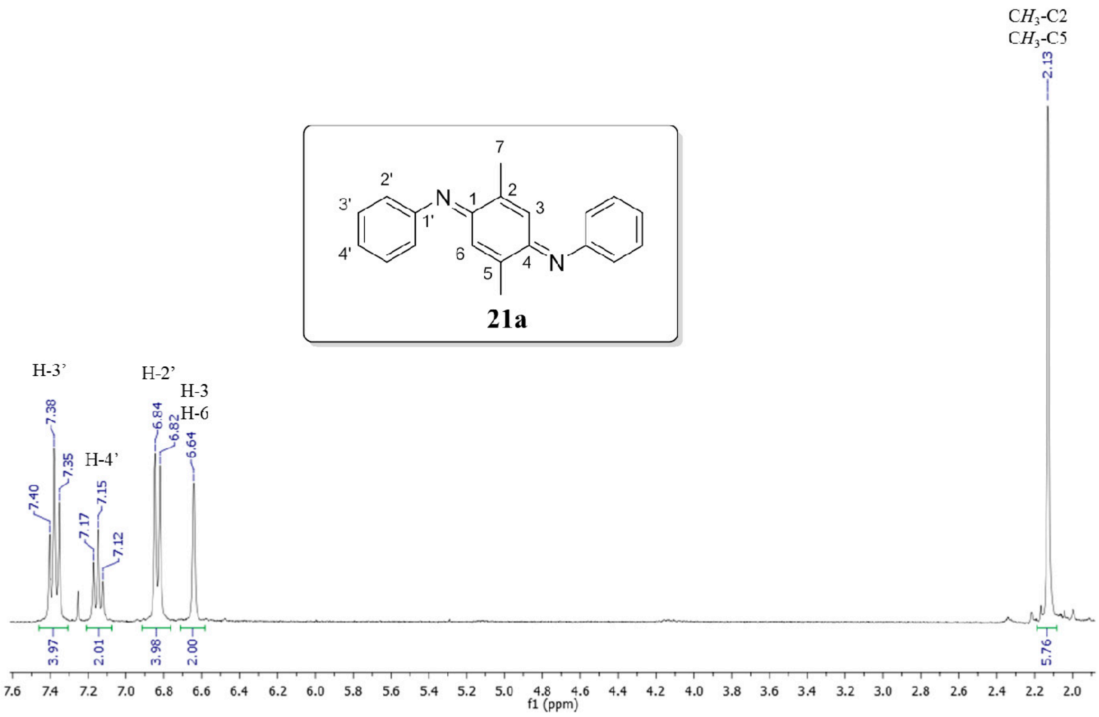

^{1}H-NMR ($CDCl_3$, 300 MHz) spectrum of **21a**

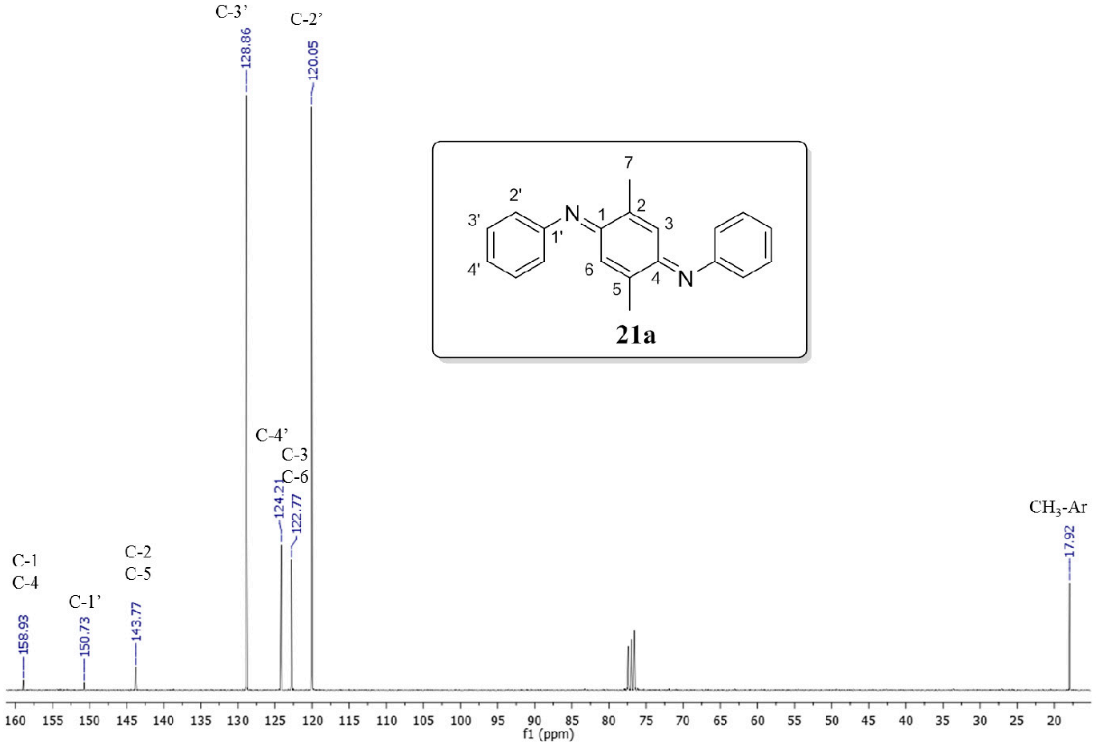

^{13}C-NMR ($CDCl_3$, 75 MHz) spectrum of **21a**

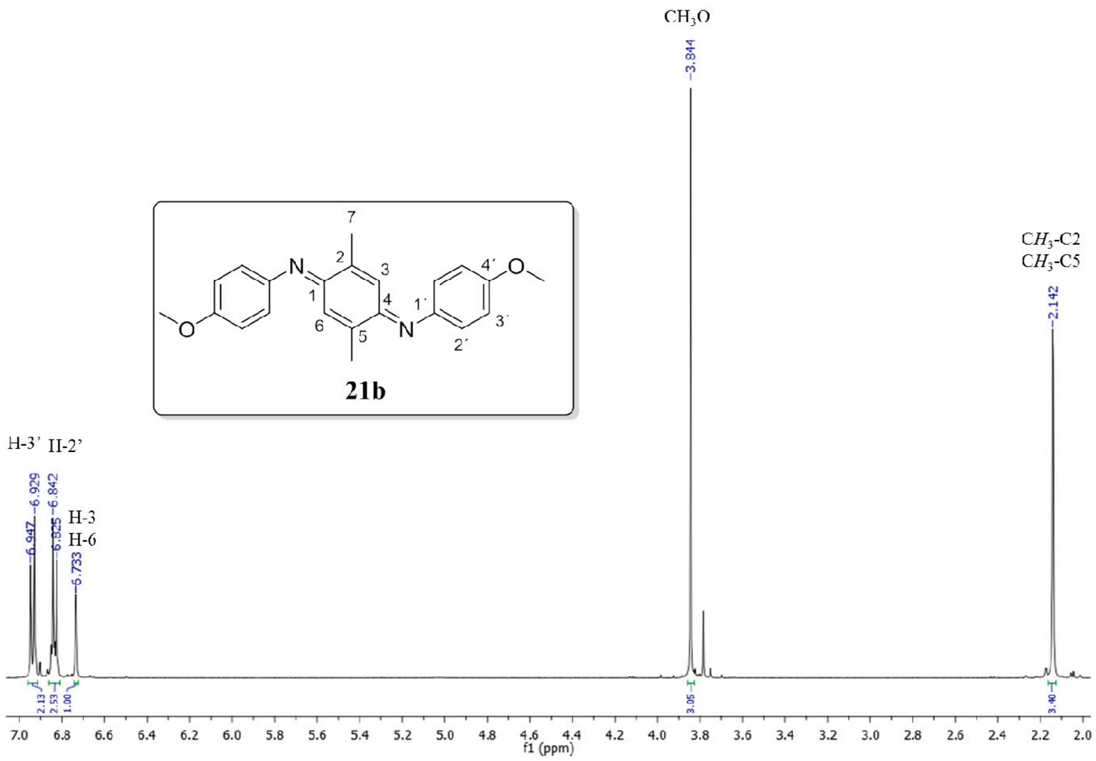

^{1}H-NMR ($CDCl_3$, 500 MHz) spectrum of **21b**

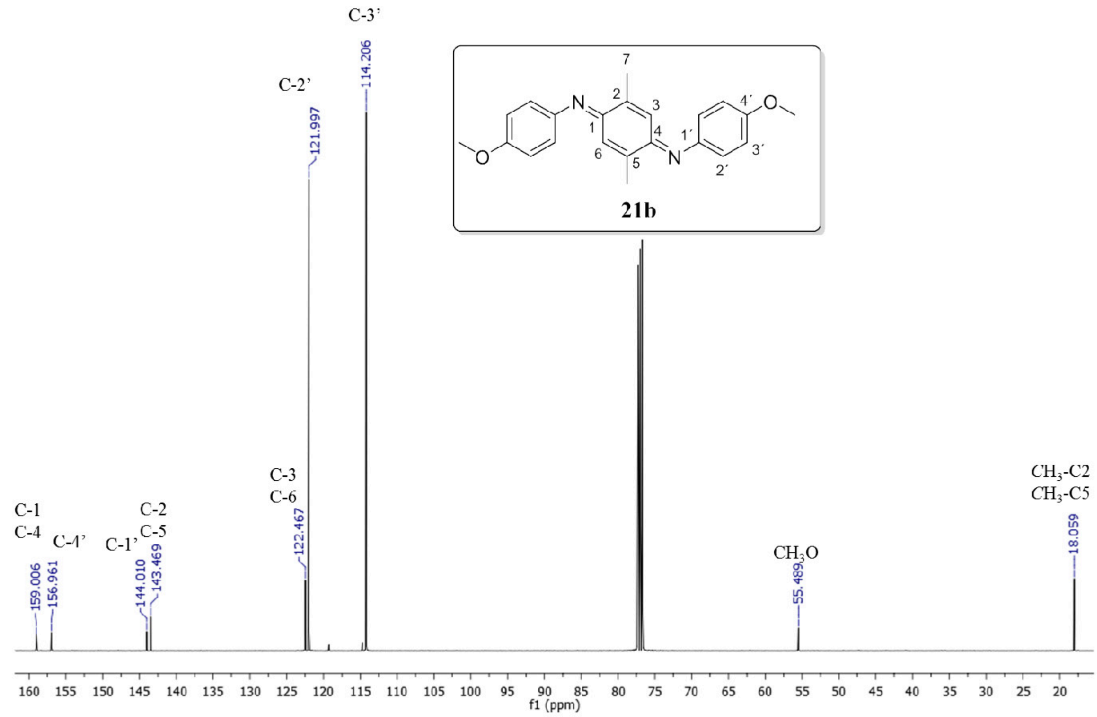

^{13}C-NMR ($CDCl_3$, 125 MHz) spectrum of **21b**

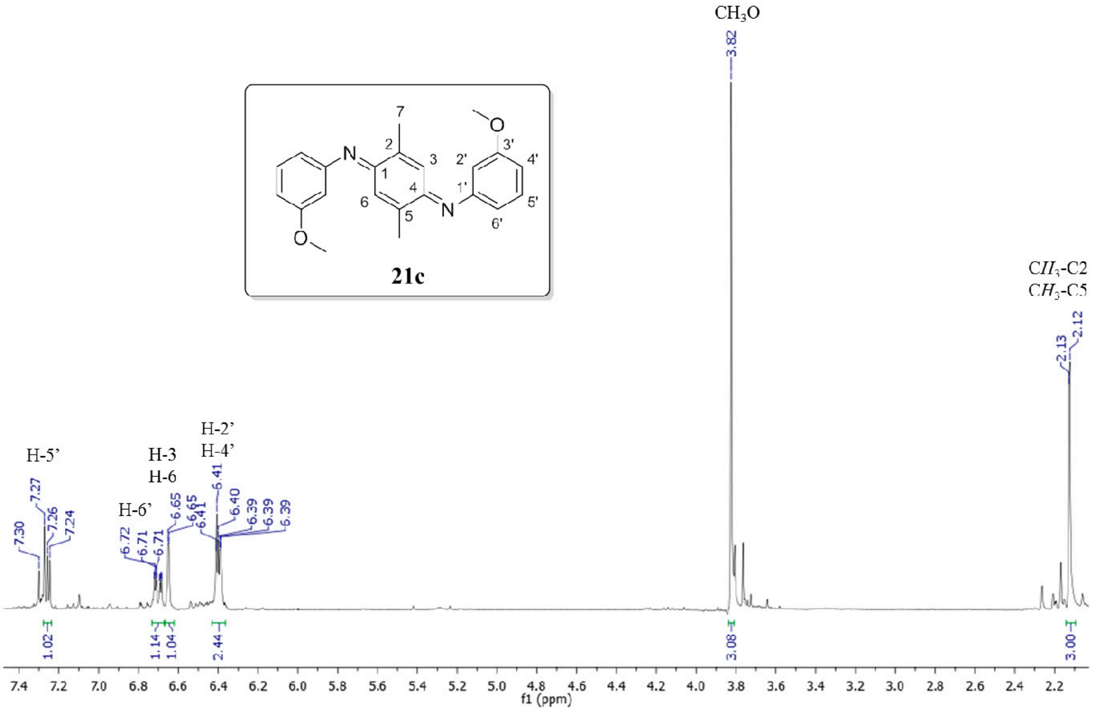

^{1}H-NMR ($CDCl_3$, 300 MHz) spectrum of **21c**

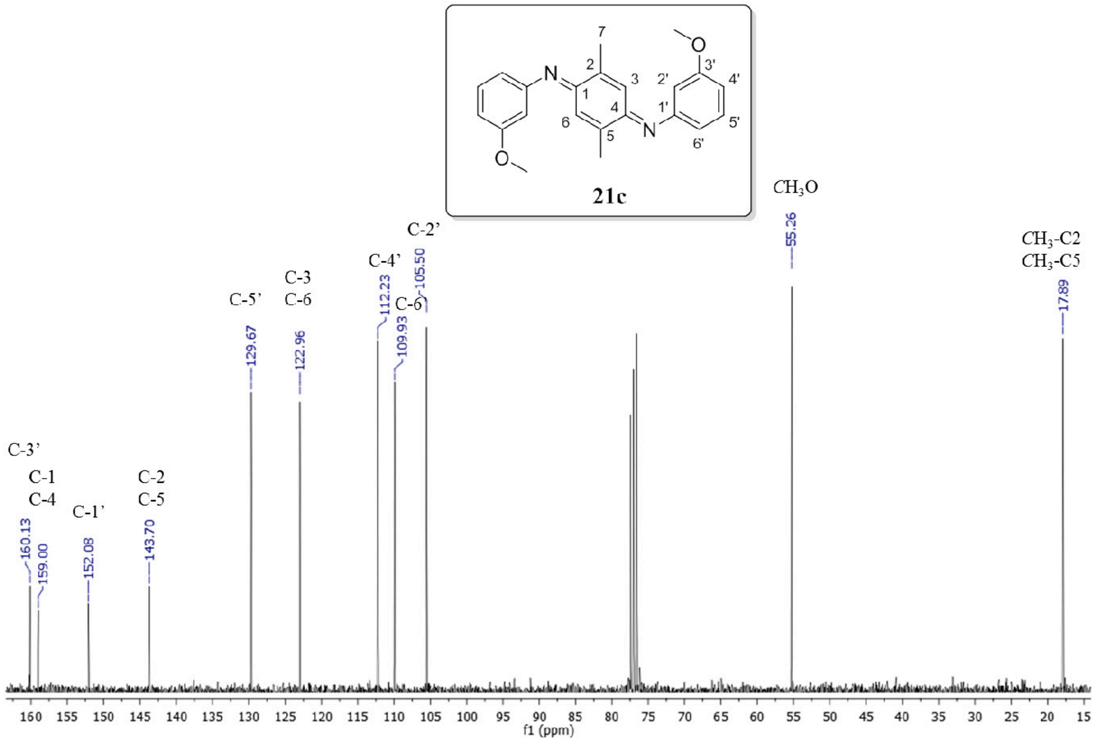

^{13}C-NMR ($CDCl_3$, 75 MHz) spectrum of **21c**

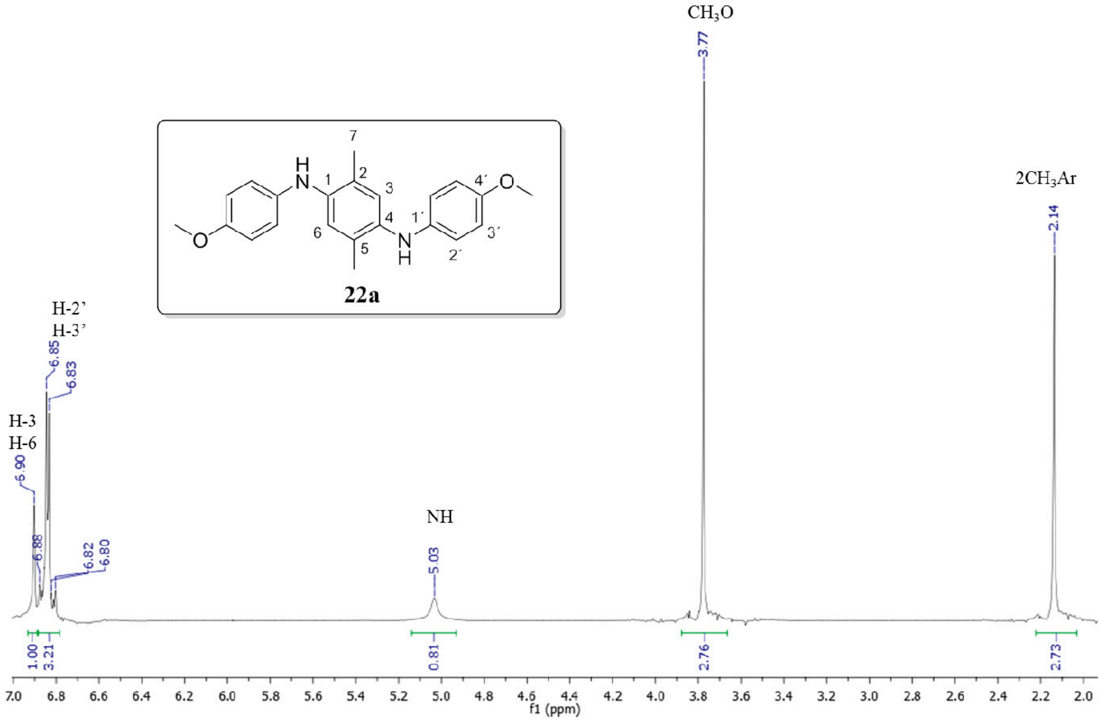

^{1}H-NMR ($CDCl_3$, 500 MHz) spectrum of **22a**

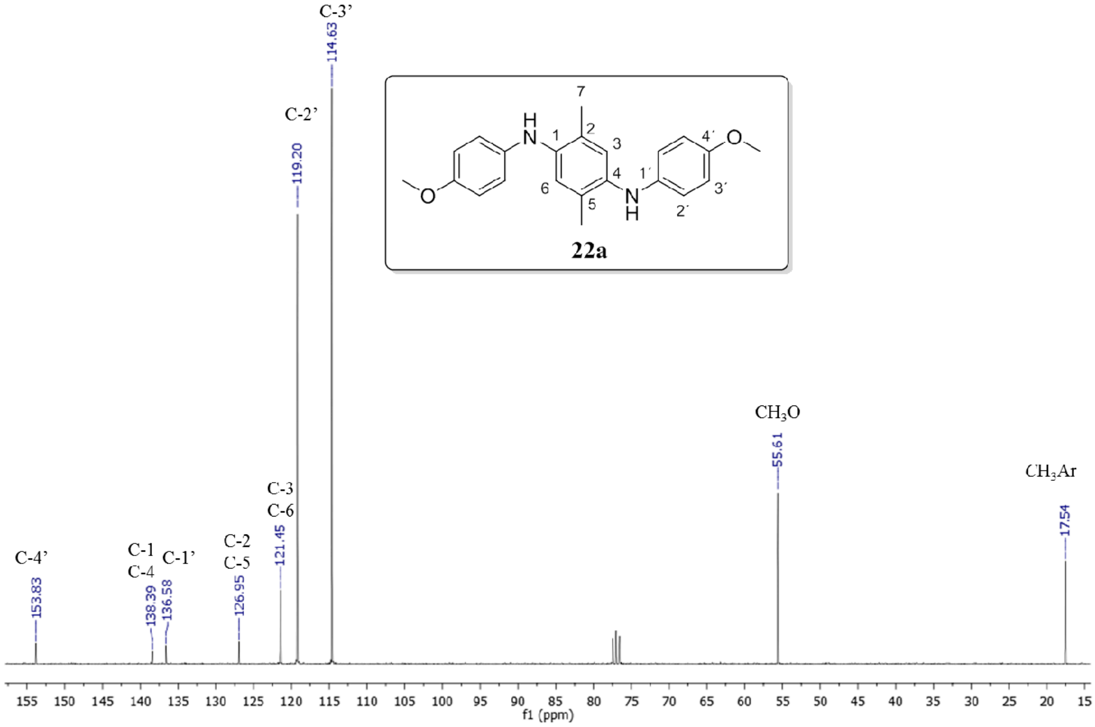

^{13}C-NMR ($CDCl_3$, 125 MHz) spectrum of **22a**

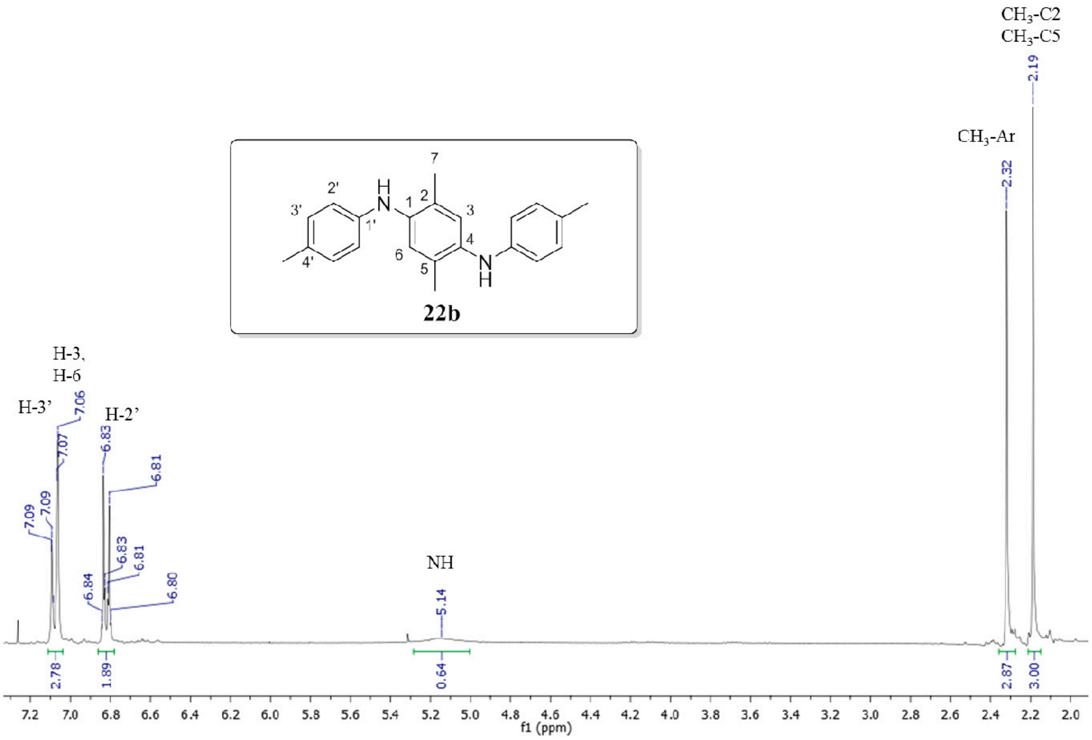

^{1}H-NMR ($CDCl_3$, 300 MHz) spectrum of **22b**

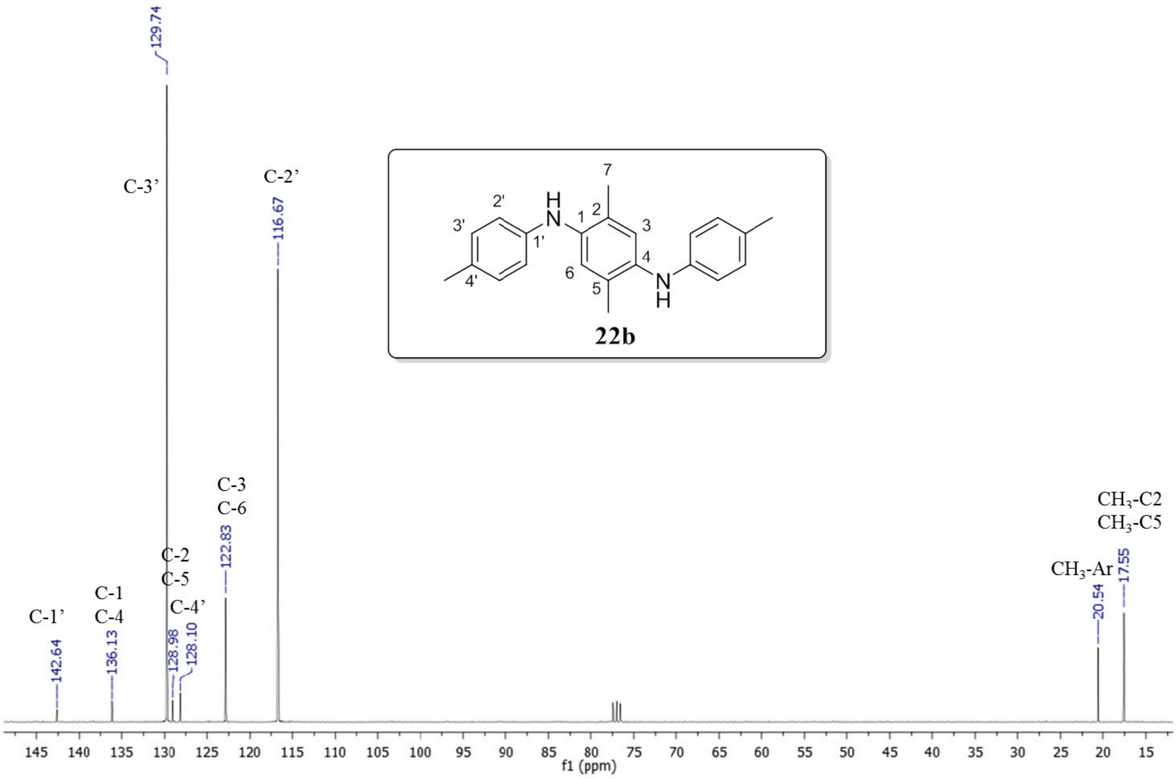

^{13}C-NMR ($CDCl_3$, 75 MHz) spectrum of **22b**

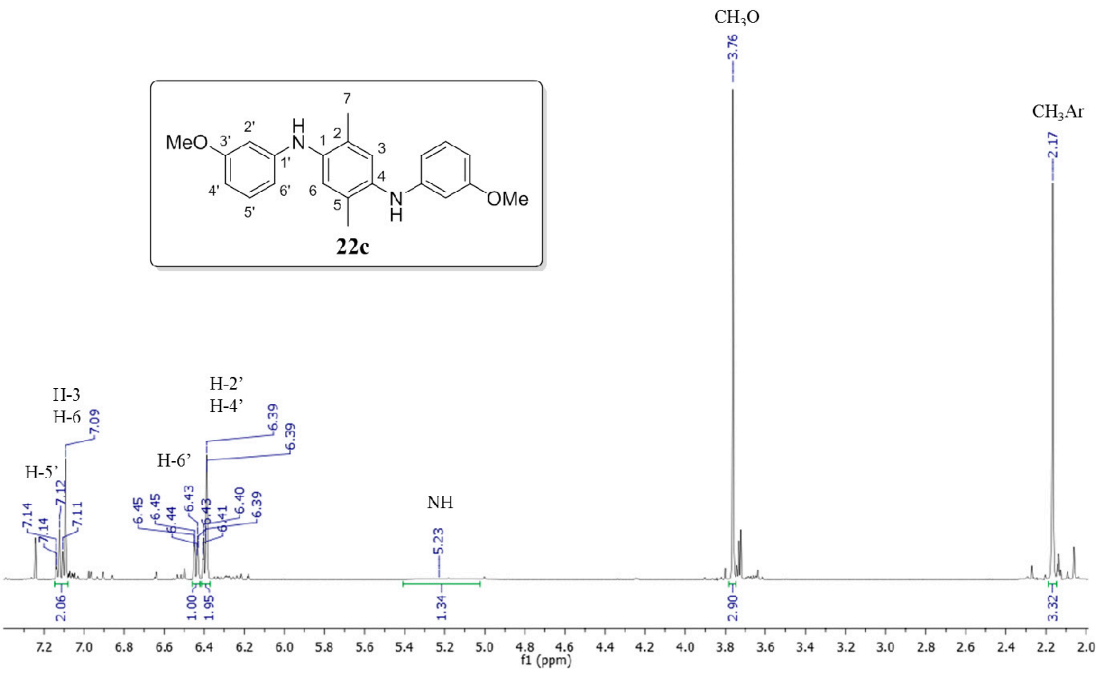

^{1}H-NMR (CDCl$_3$, 500 MHz) spectrum of **22c**

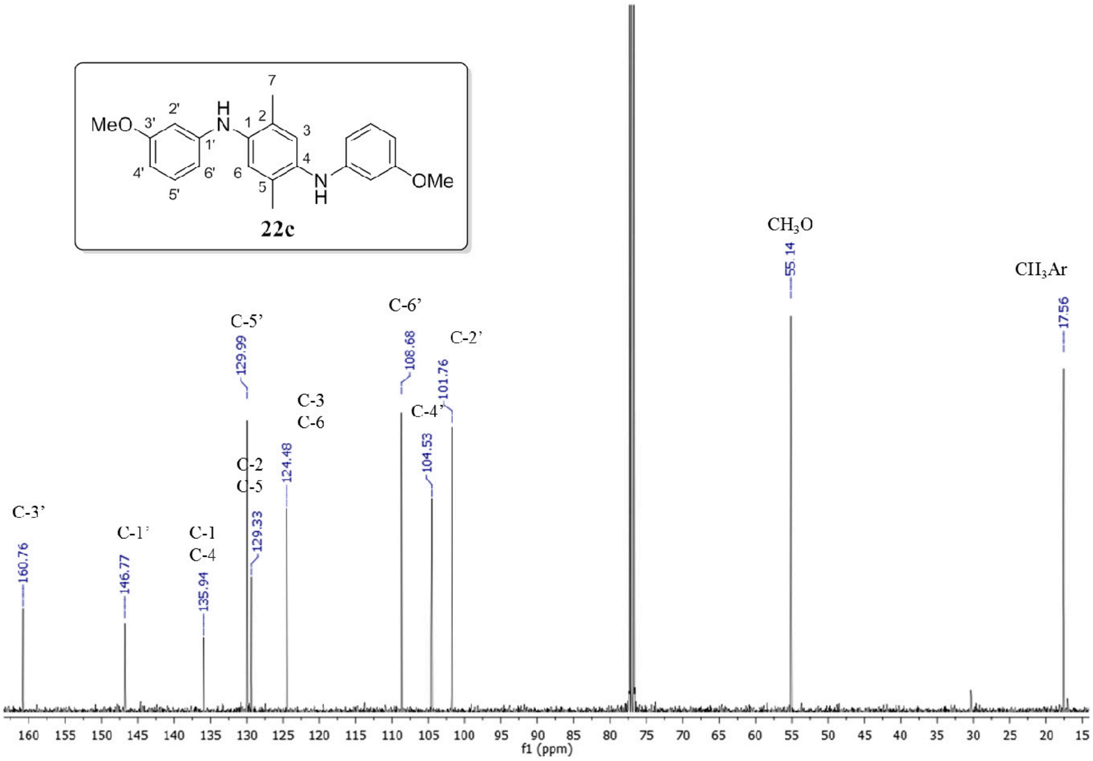

^{13}C-NMR (CDCl$_3$, 125 MHz) spectrum of **22c**

2. X-ray tables and crystallographic data of 8a

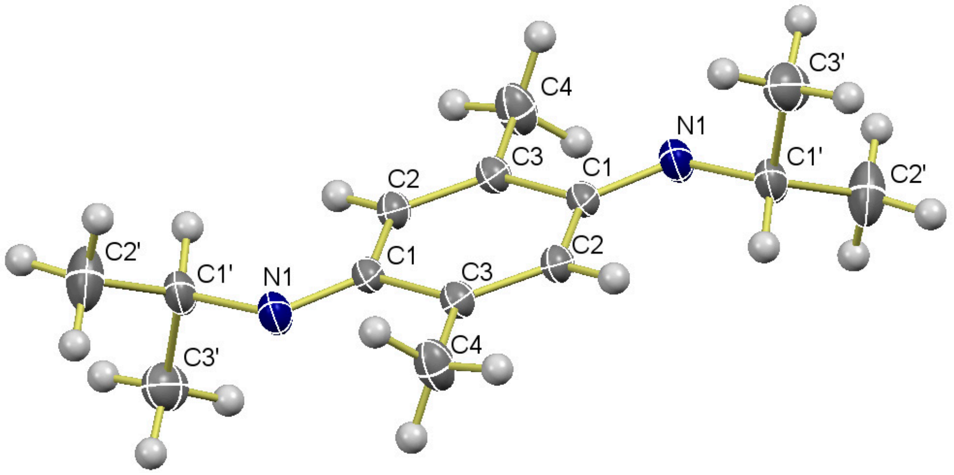

Table S1. Crystal data and structure refinement for **8a** (**CCDC 1429959**).

Identification code	0117-jt	
Empirical formula	C9.33 H14.67 N1.33	
Formula weight	24/May/1900	
Temperature	292(2) K	
Wavelength	0.71073 Å	
Crystal system	monoclinic	
Space group	P 1 21/a 1	
Unit cell dimensions	a = 9.3369(19) Å	a = 90°
	b = 7.7080(4) Å	b = 142.07 (4)°
	c = 15.920(3) Å	g = 90°
Volume	704.3(2) Å3	
Z	3/Jan/1900	
Density (calculated)	1.030 Mg/m^3	
Absorption coefficient	0.061 mm^{-1}	
F(000)	27/Aug/1900	
Crystal size	0.57 × 0.55 × 0.51 mm^3	
Theta range for data collection	3.36 to 32.79°.	
Index ranges	−13 ≤ h ≤ 13, −11 ≤ k ≤ 11, −22 ≤ l ≤ 24	
Reflections collected	14/Jan/1921	
Independent reflections	2391 [R(int) = 0.0188]	
Completeness to theta = 27.50°	0/Jan/1900	
Max. and min. transmission	0.9697 and 0.9662	
Refinement method	Full-matrix least-squares on F^2	
Data/restraints/parameters	2391/0/117	
Goodness-of-fit on F^2	1/Jan/1900	
Final R indices [I > 2sigma(I)]	R^1 = 0.0590, wR^2 = 0.1484	
R indices (all data)	R^1 = 0.0817, wR^2 = 0.1652	
Largest diff. peak and hole	0.294 and −0.136 e·$Å^{-3}$	

Table S2. Atomic coordinates (×10^4^) and equivalent isotropic displacement parameters ($Å^2 \times 10^3$) for **8a**. U(eq) is defined as one third of the trace of the orthogonalized U^{ij} tensor.

	x	y	z	U(eq)
C(1)	1262(2)	9300(1)	1287(1)	37(1)
C(2)	1573(2)	8640(1)	579(1)	39(1)
C(3)	405(2)	9262(1)	-628(1)	39(1)
N(1)	2291(2)	8739(1)	2432(1)	48(1)
C(4)	759(3)	8542(2)	-1328(2)	59(1)
C(1′)	3999(2)	7330(2)	3183(1)	51(1)
C(3′)	2789(3)	5649(2)	2820(2)	68(1)
C(2′)	5890(4)	7746(3)	4697(2)	85(1)

Table S3. Bond lengths [Å] and angles [°] for **8a**.

C(1)-N(1)	1.2918(15)
C(1)-C(2)	1.4594(16)
C(1)-C(3)#1	1.4756(16)
C(2)-C(3)	1.3428(17)
C(2)-H(11)	0.945(15)
C(3)-C(1)#1	1.4756(16)
C(3)-C(4)	1.4969(18)
N(1)-C(1')	1.4644(17)
C(4)-H(10)	0.97(2)
C(4)-H(8)	0.99(2)
C(4)-H(9)	0.97(3)
C(1′)-C(3′)	1.504(2)
C(1′)-C(2')	1.522(3)
C(1′)-H(1′)	0.990(15)
C(3′)-H(4)	1.01(2)
C(3′)-H(2)	0.95(2)
C(3′)-H(3)	0.98(2)
C(2′)-H(5)	1.00(3)
C(2′)-H(6)	0.97(2)
C(2′)-H(7)	1.00(3)
N(1)-C(1)-C(2)	126.43(10)
N(1)-C(1)-C(3)#1	116.60(11)
C(2)-C(1)-C(3)#1	116.97(9)
C(3)-C(2)-C(1)	123.26(10)
C(3)-C(2)-H(11)	119.0(8)
C(1)-C(2)-H(11)	117.7(8)
C(2)-C(3)-C(1)#1	119.76(11)
C(2)-C(3)-C(4)	121.69(11)
C(1)#1-C(3)-C(4)	118.54(10)
C(1)-N(1)-C(1′)	121.24(11)
C(3)-C(4)-H(10)	111.9(11)
C(3)-C(4)-H(8)	109.9(12)
H(10)-C(4)-H(8)	108.1(17)
C(3)-C(4)-H(9)	111.0(15)
H(10)-C(4)-H(9)	109.5(18)
H(8)-C(4)-H(9)	106.3(19)
N(1)-C(1′)-C(3′)	108.72(12)

N(1)-C(1′)-C(2′)	107.00(14)
C(3')-C(1′)-C(2′)	111.80(15)
N(1)-C(1′)-H(1′)	112.8(9)
C(3′)-C(1′)-H(1′)	107.7(9)
C(2′)-C(1′)-H(1′)	108.9(9)
C(1′)-C(3′)-H(4)	108.1(11)
C(1′)-C(3′)-H(2)	110.4(11)
H(4)-C(3′)-H(2)	106.8(15)
C(1')-C(3′)-H(3)	112.8(12)
H(4)-C(3′)-H(3)	108.7(16)
H(2)-C(3′)-H(3)	109.8(16)
C(1′)-C(2′)-H(5)	110.5(16)
C(1′)-C(2′)-H(6)	110.3(14)
H(5)-C(2′)-H(6)	105.8(19)
C(1')-C(2′)-H(7)	111.0(17)
H(5)-C(2′)-H(7)	106(2)
H(6)-C(2′)-H(7)	113(2)

Symmetry transformations used to generate equivalent atoms: #1 − x, −y + 2, −z.

Table S4. Anisotropic displacement parameters ($Å^2 \times 10^3$) for **8a**. The anisotropic displacement factor exponent takes the form: $-2 \times \pi^2[h^2a^{*2}U^{11} + ... + 2\ h\ k\ a^*\ b^*\ U^{12}]$.

	U11	U22	U33	U23	U13	U12
C(1)	35(1)	35(1)	36(1)	7(1)	27(1)	1(1)
C(2)	38(1)	34(1)	42(1)	9(1)	31(1)	6(1)
C(3)	41(1)	34(1)	41(1)	5(1)	32(1)	2(1)
N(1)	49(1)	49(1)	42(1)	14(1)	35(1)	10(1)
C(4)	75(1)	54(1)	60(1)	14(1)	57(1)	19(1)
C(1′)	48(1)	57(1)	44(1)	21(1)	35(1)	16(1)
C(3′)	70(1)	53(1)	85(1)	21(1)	62(1)	17(1)

Table S5. Hydrogen coordinates ($\times 10^4$) and isotropic displacement parameters ($Å^2 \times 10^3$) for**8a**.

	x	y	z	U(eq)
H(11)	2600(30)	7698(19)	978(14)	51(4)
H(1′)	4720(30)	7213(19)	2956(15)	54(4)
H(4)	2170(30)	5720(20)	3127(19)	89(6)
H(10)	1740(30)	7510(30)	-867(19)	87(6)

Table S6. Torsion angles [°] for **8a**.

N(1)-C(1)-C(2)-C(3)	178.54(11)
C(3)#1-C(1)-C(2)-C(3)	−0.88(18)
C(1)-C(2)-C(3)-C(1)#1	0.90(19)
C(1)-C(2)-C(3)-C(4)	−179.53(12)
C(2)-C(1)-N(1)-C(1′)	1.28(19)
C(3)#1-C(1)-N(1)-C(1′)	−179.29(10)
C(1)-N(1)-C(1')-C(3′)	−95.84(16)
C(1)-N(1)-C(1')-C(2′)	143.26(16)

Symmetry transformations used to generate equivalent atoms: #1 − x, −y + 2, −z.
